# Genome Sequencing and analyses of Two Marine Fungi from the North Sea Unraveled a Plethora of Novel Biosynthetic Gene Clusters

**DOI:** 10.1038/s41598-018-28473-z

**Published:** 2018-07-05

**Authors:** Abhishek Kumar, Jens Laurids Sørensen, Frederik Teilfeldt Hansen, Mikko Arvas, Muhammad Fahad Syed, Lara Hassan, J. Philipp Benz, Eric Record, Bernard Henrissat, Stefanie Pöggeler, Frank Kempken

**Affiliations:** 1 Department of Genetics & Molecular Biology in Botany, Institute of Botany, Christian-Albrechts-University at Kiel, Kiel, Germany; 2 Department of Chemistry and Bioscience, Aalborg University, Niels Bohrs Vej 8, DK-6700 Esbjerg, Denmark; 3 Department of Biochemistry, McGill University, Francesco Bellini Life Sciences Building, 3649 Promenade Sir William Osler, Montreal, QC H3G 0B1 Canada; 4 VTT Technical Research Centre of Finland Ltd, Tietotie 2, FI-02044 VTT Espoo, Finland; 5 Biocomputing Platforms Ltd, Tekniikantie 14, FI-02150 Espoo, Finland; 6 Holzforschung München, TUM School of Life Sciences Weihenstephan, Technische Universität München, Hans-Carl-von-Carlowitz-Platz 2, Freising, Germany; 7 INRA, Aix-Marseille Université, UMR1163, Biodiversité et Biotechnologie Fongiques, Marseille, France; 8 Centre National de la Recherche Scientifique (CNRS), UMR7257, Université Aix-Marseille, Marseille, 13288 France; 9 INRA, USC 1408 AFMB, F-13288 Marseille, France; 10 Department of Biological Sciences, King Abdulaziz University, Jeddah, Saudi Arabia; 11 Institute of Microbiology and Genetics, Department of Genetics of Eukaryotic Microorganisms, Georg-August University, Göttingen, Germany

## Abstract

Marine Fungi are potent secondary metabolite producers. However, limited genetic information are available their biosynthetic gene clusters (BGCs) and their biotechnological applications. To overcome this lack of information, herein, we used next-generation sequencing methods for genome sequencing of two marine fungi, isolated from the German Wadden Sea, namely *Calcarisporium* sp. KF525 and *Pestalotiopsis* sp. KF079. The assembled genome size of the marine isolate *Calcarisporium* sp. KF525 is about 36.8 Mb with 60 BGCs, while *Pestalotiopsis s*p. KF079 has a genome size of 47.5 Mb harboring 67 BGCs. Of all BGCs, 98% and 97% are novel clusters of *Calcarisporium sp*. and *Pestalotiopsis sp*., respectively. Only few of the BGCs were found to be expressed under laboratory conditions by RNA-seq analysis. The vast majority of all BGCs were found to be novel and unique for these two marine fungi. Along with a description of the identified gene clusters, we furthermore present important genomic features and life-style properties of these two fungi. The two novel fungal genomes provide a plethora of new BGCs, which may have biotechnological applications in the future, for example as novel drugs. The genomic characterizations will provide assistance in future genetics and genomic analyses of marine fungi.

## Introduction

Marine fungi have been much neglected for a long time, although first studies on marine fungi were published decades ago^1^. More recently, the phylogeny of some marine-derived fungi was elucidated by analysis of their small ribosomal RNA subunit sequences^2^. Thirty-six new marine lineages were isolated from six European near-shore sites. The isolates were dominated by chytrids, but also a few filamentous fungi and many ascomycetous and basidiomycetous yeasts were described^2^. Additionally, there is an effort to identify and catalog marine species in European marine environments^3^. Nevertheless, the number of cultivated marine fungi merely comprises some 470 species^4^ belonging to 244 genera. This would be less than one percent of all known fungi. Molecular analysis of rDNA sequences have contributed to a somewhat higher count of fungal species, but they still constitute a surprisingly low number of taxonomic units found in marine environments^2^.

Currently the estimated number of marine fungal species is over 10,000^5^, but may be much higher^6^. Factors that influence whether or not marine fungi are present in any particular location include the water temperature, water salinity, the water movement, the presence of suitable substrates for colonization, the presence of propagates in the water, interspecific competition, pollution and the oxygen content of the water^7^.

The biodiversity of marine fungal isolates is mirrored by the molecular diversity of their secondary metabolites^8–12^. Yet, these studies are mainly chemistry based, and marine fungi remain tremendously underexplored with regard to species, distribution and applications^12,13^. As such marine-derived fungi contain a treasure chest of secondary metabolites, of which a considerable number have promising biological or pharmaceutical properties^14^. However, so far, most studies did not use whole genome sequencing to discover the enormous potential of fungi for secondary metabolite production. A study on a marine fungus isolated from a sponge revealed the entire secondary metabolite cluster for scopularide A and B, which has anti-cancer activities^15^. Secondary metabolites are low-molecular-mass organic compounds that, unlike primary metabolites, are not directly involved in growth, development or reproduction of the producing organism. Up until 2014 about 170,000 natural products have been characterized from both marine and terrestrial organisms^16^. Fungal secondary metabolites can be divided into four main chemical classes: polyketides, terpenoids, shikimic acid-derived compounds, and non-ribosomal peptides. The majority of fungal secondary metabolites derives from either non-ribosomal peptide synthetases (NRPSs) or polyketide synthases (PKSs), A few compounds represent mixed polyketide–non-ribosomal peptide compounds called NRPS-PKS hybrids^17^. Furthermore, secondary metabolites can be found in microbes of diverse environments and even chemical biogeographic distribution maps for biomedically valuable families of natural products in the environment have been created^18^. A number of these compounds have important pharmacological applications and are used as antibiotics/antibacterial drugs^19^. Genome-mining efforts indicate that the capability of fungi to produce secondary metabolites has been substantially underestimated, because many of these gene clusters are silent under standard cultivation conditions^19^. This indicated a plethora of natural products remains to be discovered.

Here we report on the genomic sequences of two North Sea-derived fungal isolates, *Calcarisporium* sp. KF525 and *Pestalotiopsis* sp. KF079 by the use of two next-generation sequencing methods. The marine-derived *Calcarisporium sp*. KF525 has a 36.8 Mb genome with 60 biosynthetic gene clusters (BGCs) and *Pestalotiopsis sp*. KF079 has a genome size of ~47.5 Mb and harbors 67 BGCs. The majority of these clusters has not been previously identified and hence provide a great source of new potential natural products. Here we make a first attempt to characterize the genome content of these two marine fungi and their BGCs using high-throughput methods.

## Results

### Overview of genomic features and RNA-Seq statistics

We generated hybrid genome assemblies of two marine fungi using two different small read sequencing methods, Roche 454 and Illumina HiSeq. 2000. The estimated genome sizes for these marine strains of *Calcarisporium* KF525 and *Pestalotiopsis* KF079 are about 36.8 Mb and about 47.5 Mb, respectively (Table 1). Genomic coverages for Calcarisporium sp. KF0525 and Pestalotiopsis KF079 and are 11.5× and 9.3× using Roche 454 reads and 219× and 169× using Illumina HiSeq 2000 reads, respectively (Table 1). The estimated numbers of genes are 15,459 and 22,626, respectively (Table 1 and Figure S1**)**. These data are within the range of genome sizes and corresponding gene contents of ascomycetes, which are rather variable in both land and marine fungi (Figure S1). The genome assembly is simplified by the fact that both *Calcarisporium* sp. KF525 and *Pestalotiopsis* sp. KF079 possess a limited number of repeated DNA sequences, which account for 1.28% and 0.97% of their respective genome sizes only (Table S1). The genomes of *Calcarisporium* KF525 and *Pestalotiopsis* KF079 contain tandem repeats with total genome size about 0.89% and 0.65% respectively. Low-complexity regions are comprised of biased composition with imperfect direct and inverted repeats (Table S1). Transposable elements make up 0.24% and 0.23%, in corresponding genomes of these two marine fungi. Among these transposable elements, retroelements are more frequent with 0.19% and 0.22% of genome size in the genomes of *Calcarisporium* KF525 and *Pestalotiopsis* KF079, respectively. Retrotransposons with long terminal repeats are the major component. Class II DNA transposons comprise only 0.05% of *Calcarisporium* genome with the Tc1-IS630-Pogo family as the major stakeholder. In contrast, *Pestalotiopsis* KF079 has a negligible number of *c*lass II DNA transposons. Most ascomycetic terrestrial fungi have a transposon content of 1–4% of fungal genome size^20^. By combing current data from terrestrial and two other marine fungi (*S*. *brevicaulis* LF580^15^ and *Cadophora malorum* Mo12^21^), it appears that marine fungi have a similar content of transposable elements.

**Table 1:** Summary of hybrid genome assemblies of two marine fungi and statistical summary of reads from Roche 454 and Illumina.

		Calcarisporium sp. KF0525	Pestalotiopsis sp. KF079
Genome size		36.8 Mb	47.5 Mb
Scaffolds		2274	318
N25^*^ (#scaffolds)		162.8 kb (43)	821.7 kb (11)
N50^*^ (#scaffolds)		95.7 kb (115)	429.3 kb (32)
N75^*^ (#scaffolds)		51.3 kb (263)	249.3 kb (67)
GC content (%)		50.6	52.1
No. of Genes		15,459	22,626
**Read Statistics**			
Roche 454	No of reads	773,371	824,828
	Total length	441,946,919	424,413,772
	Average read length	571.5	514.5
	Genomic coverage	9.3×	11.5×
Illumina	No of reads	77,557,838	79, 803, 010
	Total length	7, 833,341,638	8, 060,104,010
	Average read length	101	101
	Genomic coverage	169×	219×

^*^Length of the scaffold until which sum of lengths of scaffolds are reached to 25%, 50% and 75% of total assembled genome size are called N25, N50 and N75 respectively.

^#^Scaffolds – Number of scaffolds in the assembled genome that constitute particular N25 or N50 or N75.

Using homology-based genome annotation analyses, we were able to annotate 72% (Table S2) and 68.9% (Table S3) of encoded proteins from assembled genomes of *Calcarisporium* KF525 and *Pestalotiopsis* KF079, respectively.

We performed RNA-Seq with wild-type strains of *Calcarisporium* sp. KF525 and *Pestalotiopsis* sp. KF079 and we found a low number of only 4,401 and 6,658 genes, being expressed under laboratory conditions. This accounts for 28.5% and 29.43% of total genes in the corresponding genomes (Tables 2, S4–S5). Based on the reads per kilobase of transcript per million mapped reads (RPKM) values, we divided the expressed genes into nine tier (Tables 2, S4–S5).

**Table 2:** Overview of RNA-Seq data of wild type of two marine fungi.

Tiers	RPKM value	*Calcarisporium* sp. KF525			*Pestalotiopsis* sp. KF079		
		No. of expressed genes	% age of expressed genes	% age of all genes	No. of expressed genes	% age of expressed genes	% age of all genes
Tier #1	>3000	73	1.7	0.5	82	1.2	0.4
Tier #2	>1000 to <3000	90	2.1	0.6	112	1.7	0.5
Tier #3	>500 to <1000	83	1.9	0.5	97	1.5	0.4
Tier #4	>250 to <500	91	2.1	0.6	159	2.4	0.7
Tier #5	>100 to <250	172	3.9	1.1	304	4.6	1.2
Tier #6	>50 to <100	540	12.3	3.5	304	4.6	1.2
Tier #7	>10 to <50	654	14.9	4.2	1125	16.9	3.1
Tier #8	>1 to <10	2028	46.1	13.1	2063	31	9.1
Tier #9	>0 to <1	1000	22.7	6.5	2412	36.2	10.7
		**Non-expressed genes**	**% age of all genes**		**Non-expressed genes**		**% age of all genes**
Tier #10	0	11058	NA	71.5	15968	NA	70.6

### Phylogenomic relationships of the two marine fungi

Genome-wide annotations of *Calcarisporium sp*. and *Pestalotiopsis sp*. depicted that both marine fungi are members of the Sordariomycetes, and they are most closely related to Nectria and Fusarium species (Figure S2A). We also performed a phylogenomic analysis using the CVtree^22^, which revealed that *Calcarisporium* sp. is close to Metarhizium and Trichoderma. In contrast, *Pestalotiopsis sp*. has diverged early from other representative sordariomycetic fungi (Figure S2B) and is close to another marine fungus, *S*. *brevicaulis*^15^.

### Overview of protein domains in the two marine fungi

**Figure 1:**
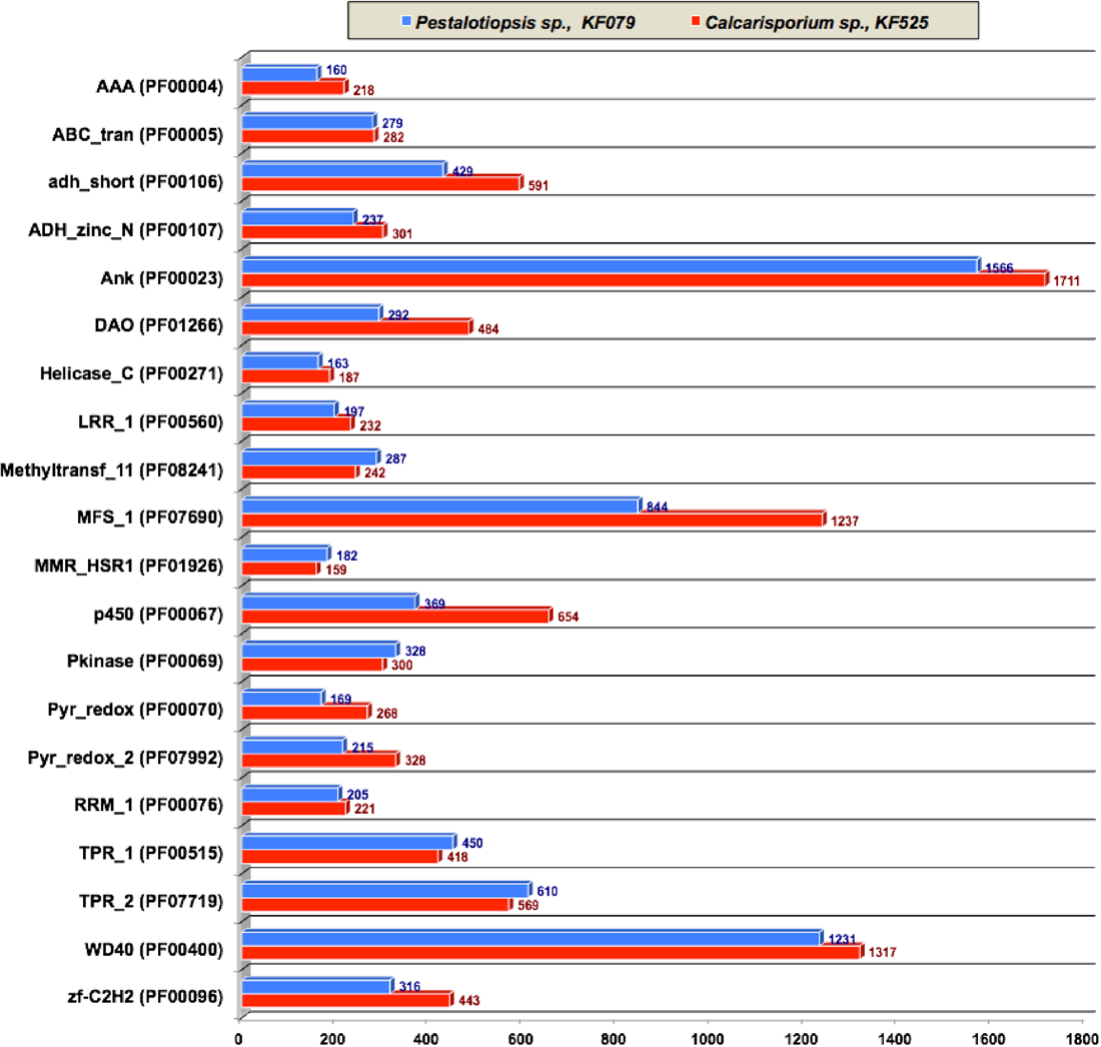
Overview of protein domains in the two marine fungal genomes. AAA (PF00004) - ATPase family associated with various cellular activities (AAA); ABC_tran (PF00005) - ABC transporter; adh_short (PF00106)-short chain dehydrogenases; ADH_zinc_N (PF00107) - Zinc-binding dehydrogenase; Ank (PF00023)- Ankyrin repeats;DAO (PF01266) - FAD dependent oxidoreductases; Helicase_C (PF00271) - Helicase conserved C-terminal domain; LRR_1 (PF00560) - Leucine Rich Repeat; Methyltransf_11 (PF08241) - methyltransferase domains; MFS_1 (PF07690) - Major Facilitator Superfamily; MMR_HSR1 (PF01926) - GTPase of unknown function; p450 (PF00067) - Cytochrome P450; Pkinase (PF00069) - Protein kinase domain; Pyr_redox (PF00070) - Pyridine nucleotide-disulphide oxidoreductase; Pyr_redox_2 (PF07992) - Pyridine nucleotide-disulphide oxidoreductase 2; RRM_1 (PF00076) - RNA recognition motif. (a.k.a. RRM, RBD, or RNP domain); TPR_1 (PF00515) - tetratricopeptide-repeat-carrying domain 1; TPR_2 (PF07719) - tetratricopeptide-repeat-carrying domain 2;WD40 (PF00400) - WD domain, G-beta repeats; zf-C2H2 (PF00096) - C2H2 type zinc finger proteins.

Independently foldable structural protein units serve as protein domains, which share common functions that have been conserved during evolution. Generally, these protein domains are surveyed in newly sequenced genomes using two databases - Pfam (version 27^23^) and Interpro (version 43^24^). *Calcarisporium sp*. and *Pestalotiopsis sp*. genomes encode 2,599 and 3,613 different protein domains localized in 7,272 (Table S6) and 10,383 proteins (Table S7), respectively. The top 20 Pfam domains are summarized in Fig. 1. We identified 1,317 and 1,231 WD domain, G-beta repeat (WD40, Pfam ID - PF00400) in *Calcarisporium sp*. and *Pestalotiopsis sp*. genomes, respectively. WD40 domains are involved in the signal transduction and regulate fungal cell differentiation processes^25^. There were furthermore a total of 1,711 and 1,566 Ankyrin repeats (Ank, PF00023) in *Calcarisporium sp*. and *Pestalotiopsis sp*. genomes, respectively. Ankyrin repeats consists of 30–34 amino acid residues long protein motifs and assist protein-protein interactions^26^. Moreover, *Calcarisporium sp*. genome encodes two tetratricopeptide-repeat-carrying domains (TPR_1, PF00515 and TPR_2, PF07719), which are found in 418 TPR_1 and 569 TPR_2 proteins, while the *Pestalotiopsis sp*. genome contains 450 TPR_1 and 610 TPR_2 proteins. These TPR motifs function as protein interaction modules^27^. Additionally, *Calcarisporium* and *Pestalotiopsis* genomes encodes 316 and 443 zinc finger proteins of the C2H2 type (zf-C2H2, PF00096), 429 and 591 short chain dehydrogenases (adh_short, PF00106), 292 and 484 FAD dependent oxidoreductases (DAO, PF01266), 328 and 300 protein kinase domains (Pkinase, PF00069) and 287 and 242 methyltransferase domains (methyltransf_11, PF08241), respectively. The entire protein domain annotations are available in Tables S6 and S7 for *Calcarisporium* and *Pestalotiopsis* genomes, respectively.

### Biosynthetic gene clusters in Calcarisporium sp

*Calcarisporium* sp. has 60 BGCs named as mCaBGC1 to mCaBGC60 (Table S8), including 24 type I PKS (T1pks) clusters, 17 nonribosomal peptide synthetase (NRPS), seven terpene and two heterocyst-type glycolipid ketosynthase (Hglks) clusters. In addition, there are four hybrid clusters and seven putative clusters. Hybrid clusters include two NRPS-t1PKS and one each of Hglks-t1PKS and T1PKS-NRPS (Table S8). 59 BGCs are unique to *Calcarisporium sp*., as these BGCs have no homologs clusters in the MiBiG database (^28^ a database of known BGCs). Hence, more than 98% of detected BGCs of *Calcarisporium* sp. are by and large novel with some identities to uncharacterized BGCs in different species. Only one cluster, mCaBGC19, is known and has a homolog in the trichotecene gene cluster from Fusarium in the MiBiG database^28^, which is the database of known and characterized BGC.

### PKS clusters in Calcarisporium sp

**Figure 2:**
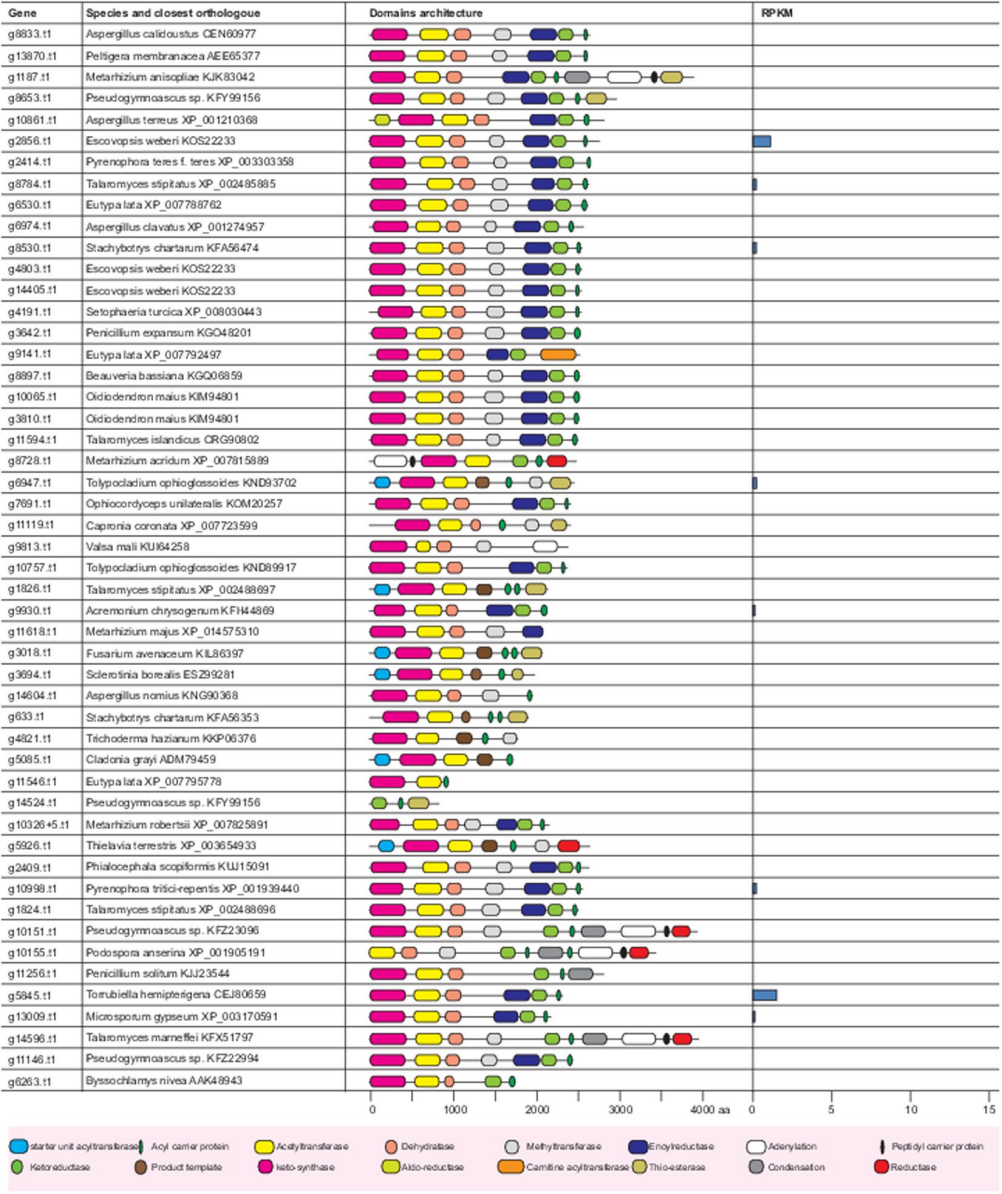
Overview of PKS genes and their protein domains in genome of marine Calcariosporium genome. Only few genes are expressed in low quality with tiers 8–9.

All the PKS genes and their expression based (on RNA-seq data) in standard growth media are shown in Fig. 2A comparison of 23 PKS gene clusters from *Calcarisporium sp*. with those from other fungi and bacteria is presented (Figure S3), while one cluster has no homologous clusters in any organisms. The PKS cluster mCaBGC21 (contig_169/39.7 kb) has 36% sequence identity with the previously uncharacterized BGC AM270194.1 c1 (localized on the contig An09c0050) from *Aspergillus niger* (Figure S3). The 43.8 kb long PKS cluster mCaBGC24 (contig178) shares 46% similarity with clusters NC015711.1 c13 from *Myxococcus fulvus* HW-1 and NC015953.1 c16 from *Streptomyces sp*. SirexAA-E (Figure S3). The PKS cluster mCaBGC25 (contig189/48.1 kb) has similarity to clusters in *Streptomyces sp*. and *Kitasatospora setae* KM-6054.

The PKS cluster mCaBGC34 (contig_280/33.6 kb) has a counterpart in several fungal genomes such as Aspergillus, Metarhizium and Trichophyton (Figure S3). The cluster mCaBGC35 (contig282/48.7 kb) shares 45% of similarity with a bacterial cluster NC_012718.1_c1 (*Burkholderia glumae* BGR1) and it is also found in genomes of *Cordyceps militaris* and *Glomerella graminicola* (Figure S3). In a similar fashion, we found that mCaBGC40 (contig_358/28 kb) has homologous clusters in several strains of *Streptomyces sp*. (Figure S3) Likewise, mCaBGC41 (contig_367/32.7 kb) as similarities with clusters in Metarhizium strains, but at the same time, it has 30% similarity with a cluster from *Nostoc punctiforme*.

### NRPS clusters in *Calcarisporium*

**Figure 3:**
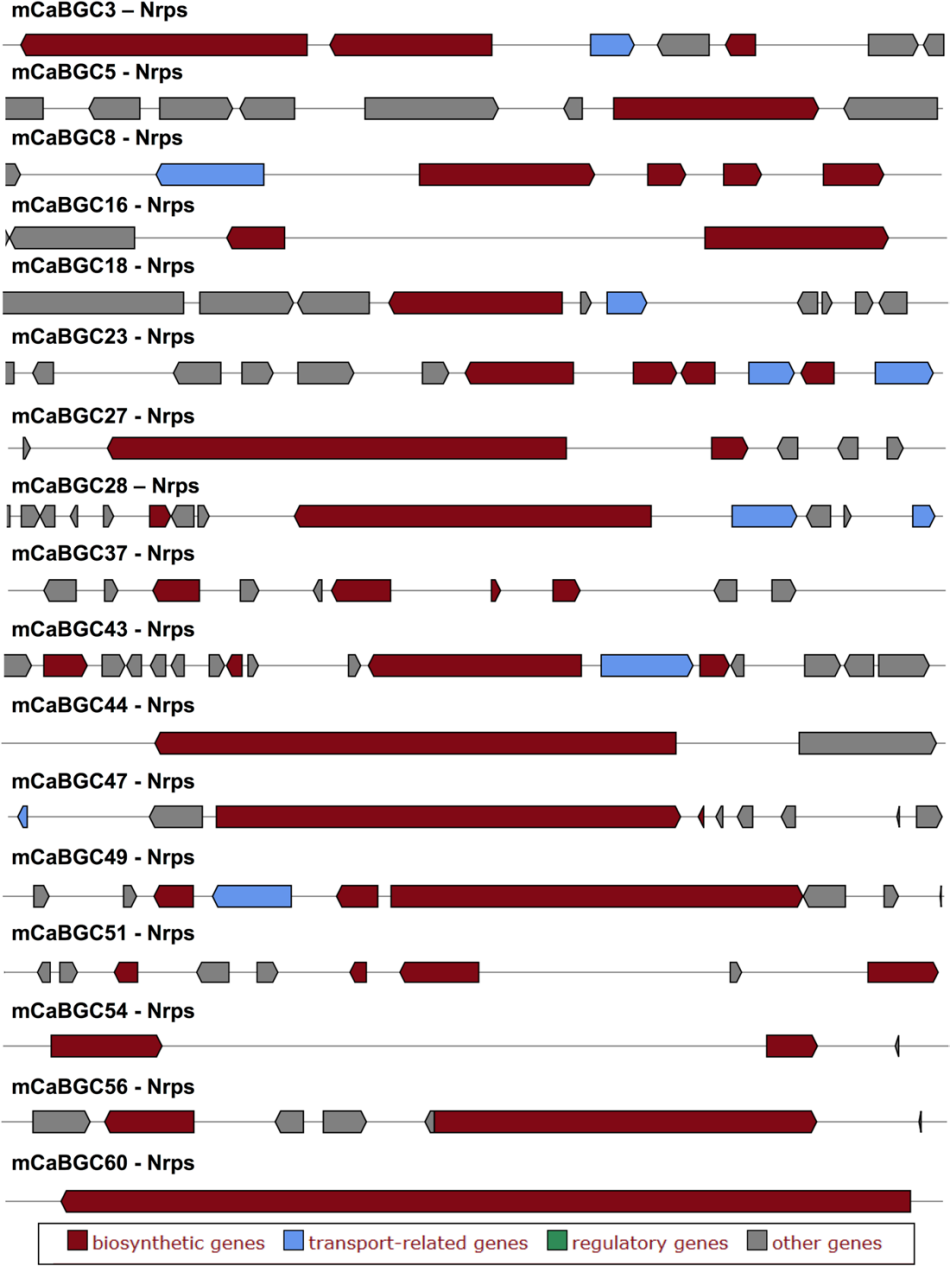
Biosynthetic gene clusters encoding NRPS in *Calcarisporium* sp. KF525 genome.

There are seventeen BGCs, representing NRPS clusters in the *Calcarisporium* genome (Fig. 3, Table S6 and Figure S3). Fifteen of these NRPS clusters have very low sequence identities (with any known clusters), while two NRPS clusters, mCaBGC44 (contig400/13.4 kb) and mCaBGC60 (contig1101/12.9 kb) have no homologs in the databases (Figure S3).

### Terpene clusters in *Calcarisporium sp*

**Figure 4:**
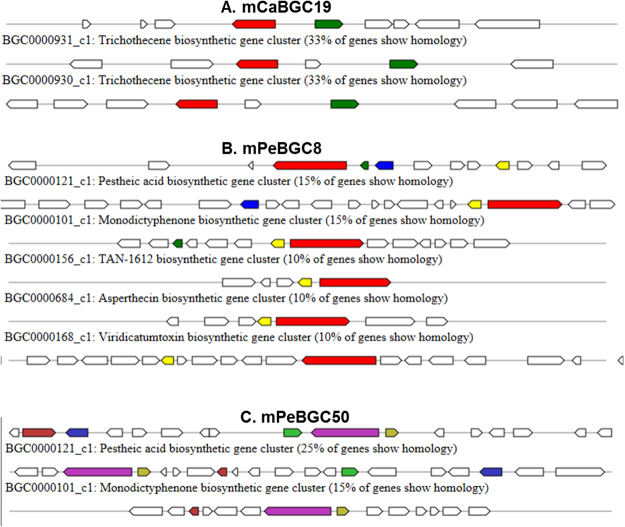
Overview of known biosynthetic gene clusters in MiBiG database **(A)** mCaBGC19 from *Calcarisporium* sp. KF525 **(B)** mPeBGC8 from *Pestalotiopsis* sp. KF079 **(C)** mPeBGC50 from *Pestalotiopsis* sp. KF079.

A trichotecene gene cluster (mCaBGC19, 21.2 kb) is localized on contig165 and it has homologs in several Fusarium strains (Fig. 4 and Figure S3). This cluster shares 33% of similarity with a trichothecene BGC from *Fusarium graminearum* (MIBiG BGC-ID - BGC0000931_c1, Table S8).

### Hybrid clusters in *Calcarisporium* sp

**Figure 5:**
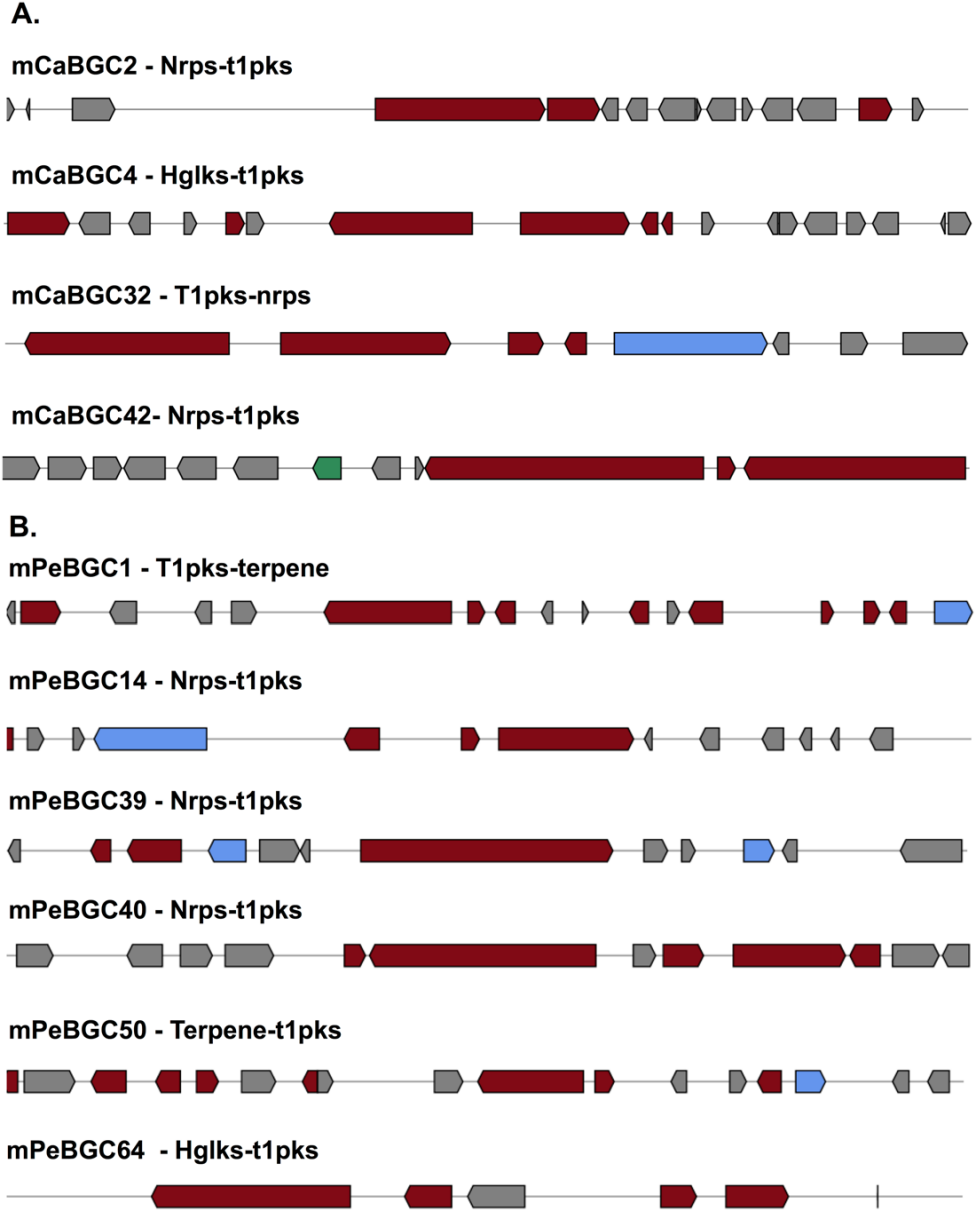
Genomic locations of hybrid biosynthetic gene clusters encoding genes in marine fungal genomes. **(A)** *Calcarisporium* sp. KF525 **(B)** *Pestalotiopsis* sp. KF079.

There are two heterocyst-type glycolipid ketosynthase (Hglks) clusters namely mCaBGC33 (contig274, size 27.4 kb) and mCaBGC46 (contig422, size 39.6 kb) in the Calcarisporium genome (Fig. 5). A cluster similar to mCaBGC33 is found in *Saccharopolyspora spinosa* (AY007564.1_c1) and Mycobacterium sp. MOTT36Y (NC_017904.1_c10), while the Hglks cluster mCaBGC46 has the top two hits in *Streptomyces longisporoflavus* (FJ462704.1_c1) and *Amycolatopsis orientalis* (DQ88475.1_c1, Figure S3).

mCaBGC2 is a 52.2 kb long hybrid NRPS-t1PKS cluster (Fig. 5), which shares similarities with clusters in Metarhizium strains (GL698718.1_c1 and GL698473.1_c1), *Mycobacterium thermoresistibils* (NZ_AGVE01000050.1_c1) and *Hydrogenophaga sp*. PBC (NZ_AJWL01000058.1_c1). The hybrid Hglks-t1PKS cluster mCaBGC4 (size 54.8 kb) shares similarities with clusters in *Stigmatella aurantiaca* and *Streptomyces viridochromogenes* (Figure S3). Cluster mCaBGC32 is a 37.7 kb long hybrid NRPS-t1PKS cluster, which is similar to clusters in *Vibrio mimicus* and *V*. *coralliilyticus*. The 45.8 kb long cluster mCaBGC42 represents a hybrid NRPS-t1PKS with similarity to cluster CACQ02000519.1 c1 from *Colletotrichum higginsianum* (Figure S3).

### Unknown types of BGCs in *Calcarisporium sp*

Unknown types of BGCs are putative BGCs, which were identified by using antiSMASH 3.0^29^ under the category ‘others’^29^. We found seven unknown types of BGCs in the *Calcarisporium* sp. KF525 genome. One of them, mCaBGC1, is a 42.1 kb long cluster, sharing an overall sequence identity of 33% with AP007151.1 c1 from *A*. *oryzae* (RIB40) (Figure S3). The remaining unknown-type BGCs have no significant sequence identities with any known clusters in the databases (Figure S3).

### Biosynthetic gene clusters in Pestalotiopsis sp

The 46 Mb assembled genome of *Pestalotiopsis* sp. KF079 contains 67 BGCs, which are named mPeBGC1 to mPeBGC67 (Table S9 and Figure S4). These clusters include 22 T1 PKS clusters, 12 nonribosomal peptide synthetase (NRPS) clusters, 9 terpene clusters and one each of type III PKS (T3pks) and lantipeptide clusters. Additionally, *Pestalotiopsis* KF079 has six clusters of hybrid nature such as three Nrps-t1pks and one each of Hglks-t1pks, T1pks-terpene and terpene-t1pks. On top of that sixteen clusters were detected with unknown types of biosynthetic genes (Table S9). Of all these BGCs only two are represented in the MiBiG database^28^ namely, mPeBGC8 and mPeBGC50. The remaining 65 BGCs are unique to *Pestalotiopsis*. *sp*., as there are no homologous clusters known or else similarity is too low. Hence, 65 out of 67 BGCs of *Pestalotiopsis* sp are previously uncharacterized, hence not found in MiBiG database^28^, a database of known BGCs.

### PKS clusters in Pestalotiopsis sp

The single T3pks cluster found in the genome of *Pestalotiopsis sp*., mPeBGC2, is localized on the scaffold00001 within a fragment of 41.4 kb, and this cluster has similarities with clusters in *Cordyceps militaris* and *Metarhizium anisopliae* (Figure S4). There are further 22 BGCs encoding PKS gene products in the *Pestalotiopsis sp*. genome. Cluster mPeBGC4 is localized in the scaffold00002, spanning an about 45.8 kb fragment without significant homologies with any other known cluster (Figure S4). Spanning about 45.6 kb on the scaffold00007, cluster mPeBGC8 is known in several ascomycetes and also in different *Streptomyces* strains. The cluster mPeBGC8 has no homolog, however it has very limited similarities with four known clusters (Fig. 4), namely pestheic acid biosynthetic gene cluster (BGC0000121_c1, 15% identities), monodictyphenone biosynthetic gene cluster (BGC0000101_c1, 15% identities), asperthecin biosynthetic gene cluster (BGC0000684_c1, 10% identities), TAN-1612 biosynthetic gene cluster (BGC0000156_c1, 10% identities) and viridicatumtoxin biosynthetic gene cluster (BGC0000168_c1, 10% identities). However, due to the low similarities of 10–15%, these similarities may be random. Remaining PKS encoding BGC have no significant identities with BGCs in any other species (Figure S4).

### NRPS clusters in *Pestalotiopsis* sp

**Figure 6:**
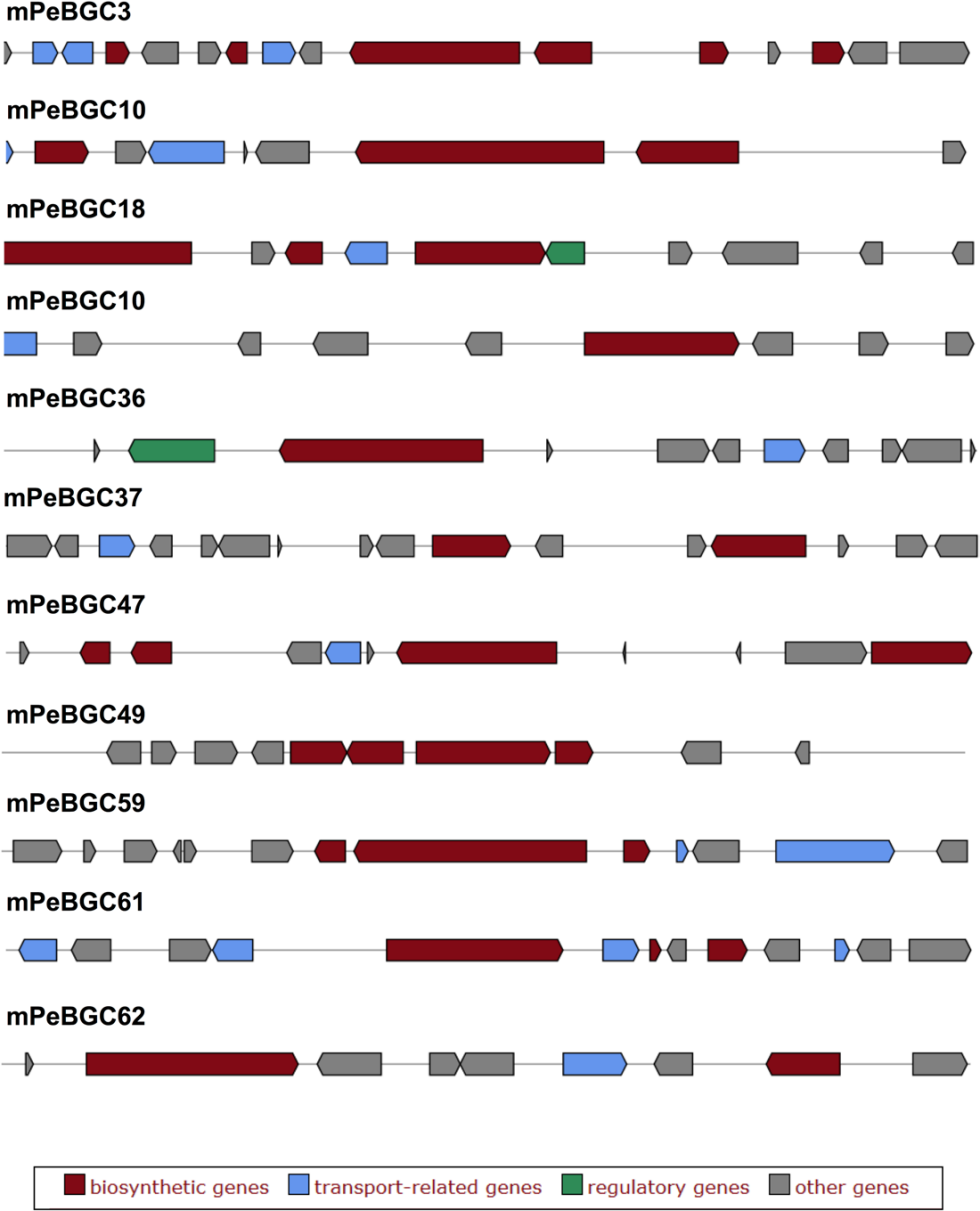
Biosynthetic gene clusters encoding NRPS genes in marine *Pestalotiopsis* genome.

There are total 11 BGCs (Fig. 6) capable of producing NRPS based compounds. The NRPS cluster mPeBGC18 (scaffold000016/46.3 kb) shares 53% homology with a bacterial cluster NC_010571.1_c1 from *Opitutus terrae* PB90–1 (Figure S4) and it is also related to lasalocid BGC (Streptomyces lasaliensis). Additionally, this cluster is also possessed by Metarhizium acridum. While mPeBGC49 (scaffold000071/46.5 kb) has top hits in *Aspergillus* and *Trichophyton* stains with 36% homology for the cluster AP007154.1_c2 from *A*. *oryzae* (Figure S4). All other NRPS clusters have no significant hits to known clusters in other organisms (Figure S4).

### Terpene clusters in *Pestalotiopsis sp*

There are nine terpene clusters in *Pestalotiopsis* genome and the first cluster mPeBGC9 is 22 kb long on the scaffold00007 (Figure S4). This terpene cluster shows roots with clusters from bacteria to fungi and is in agreement with another terpene cluster mPeBGC35 (scaffold000042/22.1 kb). The second terpene cluster mPeBGC17 is 22.1 kb long on the scaffold000016 and shares similarities with clusters in several ascomycetes, which is also the case for five other terpene clusters namely mPeBGC29 (scaffold000031/23 kb), mPeBGC31 (scaffold000036/22.6 kb), mPeBGC35 (scaffold000042/22.1 kb), mPeBGC43 (scaffold000057/23 kb) and mPeBGC63 (scaffold000156/18.3 kb). Interestingly, the mPeBGC63 cluster show similarities with trichothecene BGCs from several Fusarium strains. Two terpene clusters mPeBGC41 (scaffold000052/21.4 kb) and mPeBGC66 (scaffold000173/13.4 kb) have no significant hits in public databases (Figure S4).

### Hybrid clusters in *Pestalotiopsis sp*

There are three Nrps-t1pks hybrid clusters in the Pestalotiopsis sp. genome (Fig. 5B) namely mPeBGC14 (scaffold000013/57.1 kb), mPeBGC39 (scaffold000049/54.2 kb), and mPeBGC40 (scaffold000051/52.5 kb). The remaining three hybrid clusters in the Pestalotiopsis genome (Fig. 5B) are of the types T1pks-terpene, Terpene-t1pks and Hglks-t1pks respectively, and are called as mPeBGC1 (scaffold0000/60.4 kb), mPeBGC50 (scaffold000072/50.4 kb) and mPeBGC64 (scaffold000158/30.7 kb), respectively. None of these hybrid clusters have significant hits, even with uncharacterized clusters in other organisms (Figure S4).

### Unknown-type clusters in *Pestalotiopsis* sp

Unknown types of BGCs are putative BGCs identified by using antiSMASH 3.0^29^ and put in the category of others^29^. We found sixteen unknown-type BGCs in Pestatiopsis genome (Figure S4), without significant cluster homology with other micro-organisms

Core PKS and NRPS proteins in the two marine fungi In the *Calcarisporium KF525* genome, we identified 50 putative PKSs, including four PKS-NRPS hybrids, one PKS with an N terminal adenylation and PCP domain, six non-reducing PKSs and thirty-seven reducing PKSs (Fig. 2). The non-reducing PKS g3018 was found to be a possible orthologue of the proposed bikaverin synthase in *Fusarium avenaceum* (FAVG1_10226)^30^. Analyses of the remaining PKSs did not result in identification of orthologues with known products in other species.

#### Several bioactive compounds are identified from Calcarisporium sp

KF525^31,32^ and the most fascinating one is calcaride A, which has antibacterial properties. Calcaride A belongs to the group of macrocyclic polyesters, for which the biosynthetic routes are yet to be identified. However based on aromatic ring formation, calcaride A can be produced by two possible genes namely NR-PKS (g1826) and the HR-PKS (g1824). Nevertheless, these two genes are not expressed under laboratory conditions (Fig. 2).

**Figure 7:**
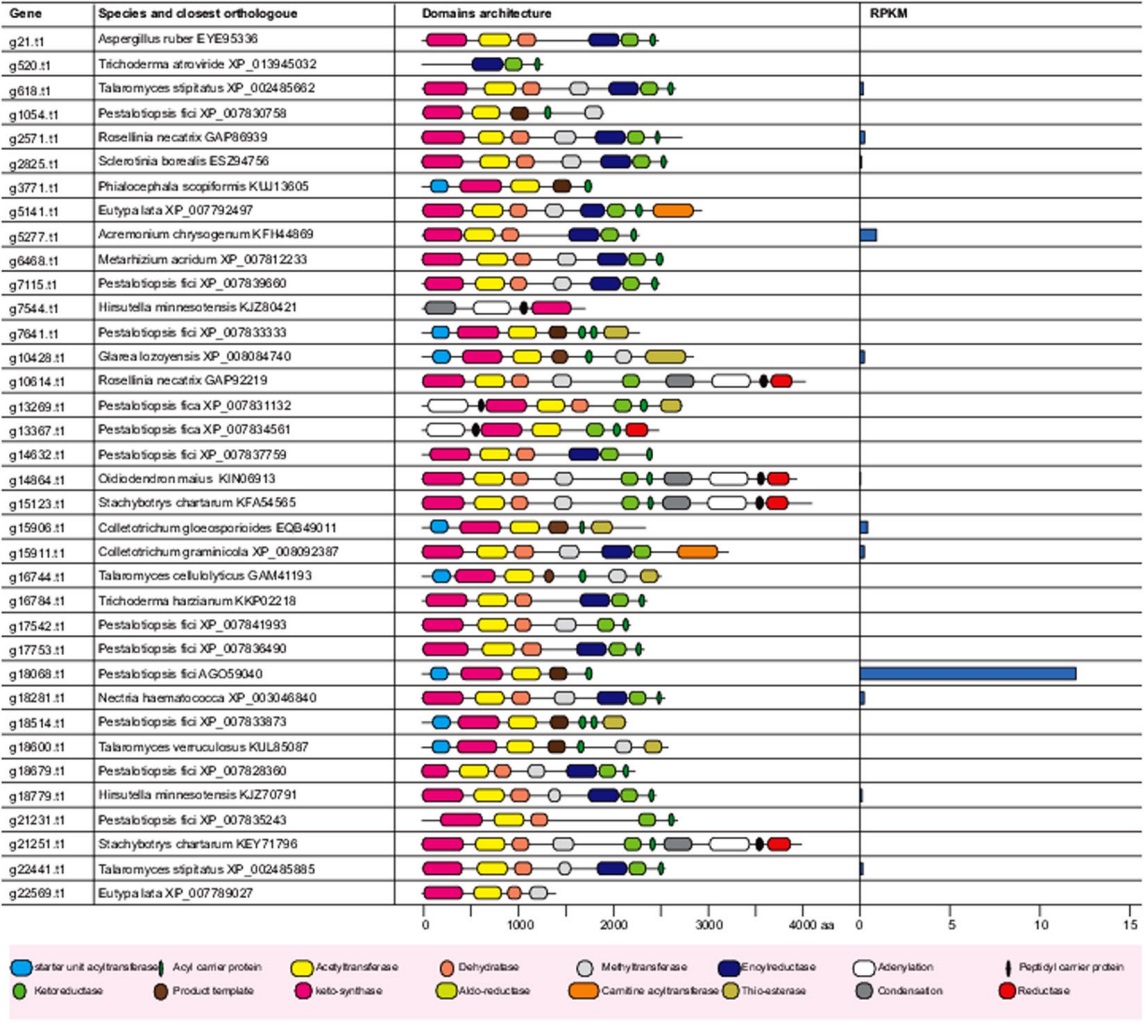
Summary of PKS genes and their protein domains in genome of marine *Pestalotiopsis* genome. Only few genes are expressed in low quality with tiers 8–9 with exception of g18068, which is in the tier 7.

In *Pestalotiopsis* sp., putative 36 PKS were identified including four PKS-NRPS hybrides, two PKSs with N terminal adenylation and PCP domains, eight non-reducing PKSs and twenty-three reducing PKSs (Fig. 7). Twelve PKSs have an orthologue in the previously sequenced *P*. *fici*^33^ including an ortholog of the *P*. *fici* pestheic acid synthase (PtaA^34^). The non-reducing PKS g18514 shares 81% homology to the melanin polyketide synthase from *Nodulisporium* sp. ATCC74245 (AAD38786.1) and an orthologue in *Pestalotiopsis microspore* has previously been shown to be required for conidial pigmentation^35^. Another likely PKS for pigmentation was also identified in g7641, which shares 60–62% identity with fusarubin synthase (*PKS3*) from members of the *Fusarium* genus^36^. The non-reducing PKS g18600 shares 42% identity to AN6448 from *Aspergillus nidulans*, which produce 3-methylorsellinic acid^37^ and it seems therefore likely that *Pestalotiopsis* sp. can produce a similar compound.

**Figure 8:**
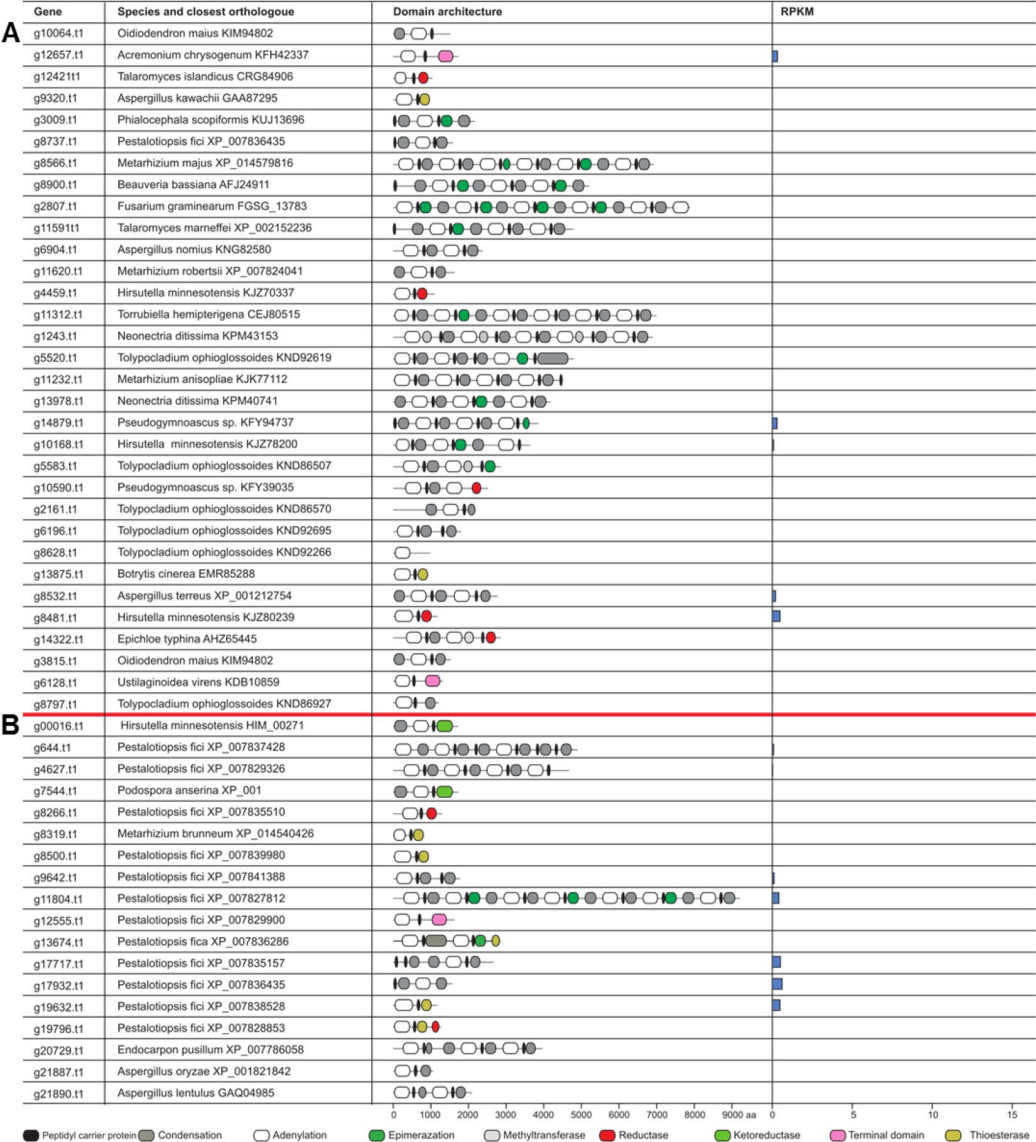
Overview of NRPS genes and their protein domains present in two marine fungal strains. **(A)** *Calcarisporium* sp. KF525 **(B)** *Pestalotiopsis* sp. KF079.

The genomes of both strains contain less NRPSs than PKSs, as we identified 32 and 18 putative NRPSs in *Calcarisporium* sp. and in *Pestalotiopsis* sp. (Fig. 8), respectively. We identified a putative iron-chelating siderophore synthetase of the ferricrocin type^38^ in both species - *Calcarisporium* sp. (g5520) and *Pestalotiopsis* sp. (g644). An additional siderophore synthetase is present in *Pestalotiopsis* sp. (g9642), as an orthologue of the SidE synthetase, which is responsible for fusarinine biosynthesis.

An orthologue of the peramine synthase from *Epichloe typhina* was identified in *Calcarisporium* sp. (g14322). Peramine is a potent insect feeding deterrent synthesized by a two-module NRPS in *Epichloë* and has been suggested to be produced in a mutualistic interaction with its host plant perennial ryegrass in order to confer protection to against insect herbivory^39^.

### Calcarisporium sp. has a MAT1–2 locus while Pestalotiopsis sp. has no detectable mating locus

**Figure 9:**
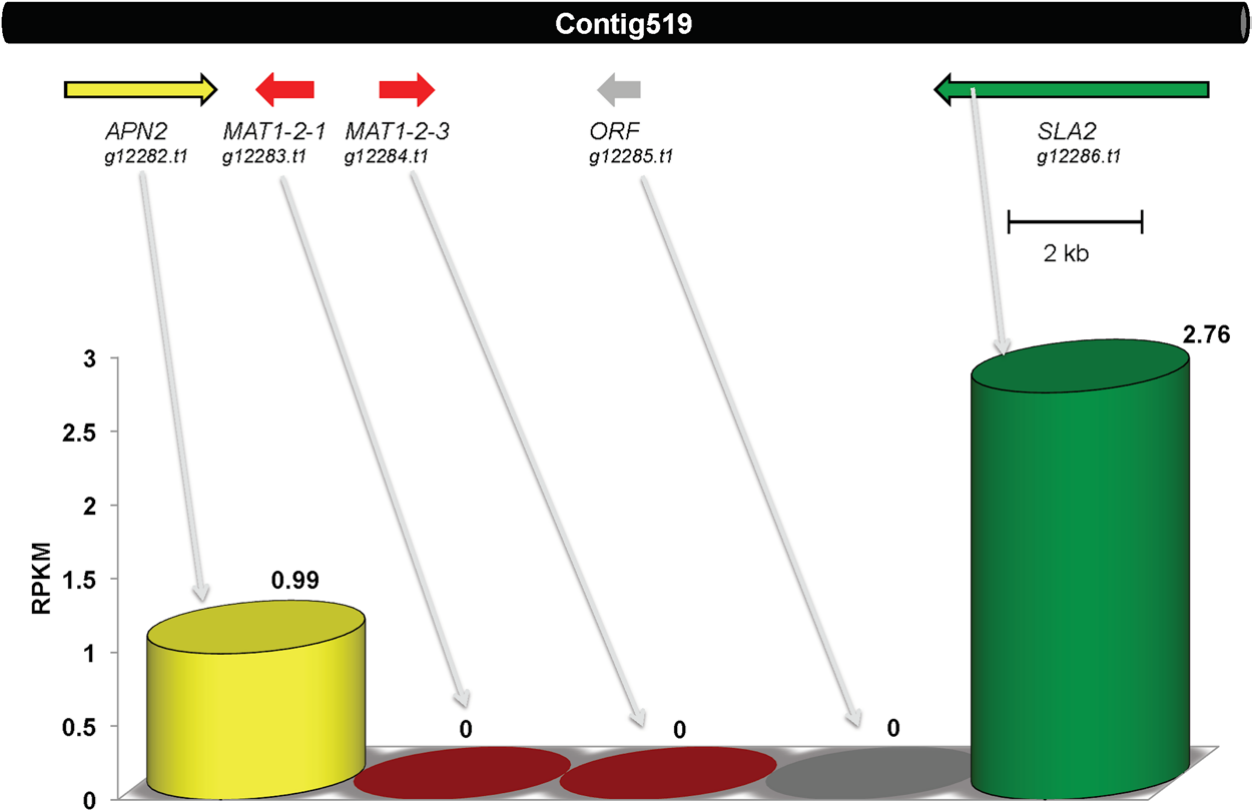
Overview of the mating-type locus MAT1–2 of *Calcarisporium sp*. KF525 on the contig519 with comparison with transcriptomic data. The positioning and transcriptional direction of the mating-type genes (red) is indicated by an arrow flanking genes *APN2* and *SLA2* are shown in yellow and green, respectively. A predicted ORF is indicated in grey.

The genomic fragments that carry the mating type genes are usually designated as the mating-type (MAT) locus, which regulates sexual reproduction in different fungi^40^. In self-sterile (heterothallic) species, mating occurs between morphologically identical partners that are only distinguished by their *MAT* locus. In filamentous ascomycetes, the *MAT* locus consists of two different DNA segments in the mating partners termed the *MAT1–1* and *MAT1–2* idiomorphs^41^. In contrast to heterothallic species, the genome of self-fertile (homothallic) filamentous ascomycetes contains genes indicative of both mating types that can be either linked or unlinked^42,43^. During genome annotation process, detection of these loci and flanking gene identification is a standard process, which aids in determination of the mode of sexual reproduction in different fungi. So far little is known about the sexual life style of marine fungi. To unravel sexual lifes of these fungi, we have performed specialized homology detections of MAT genes and corresponding loci (explained in details in Suppl. section S1). A tBLASTN search with the MAT1–2–1 HMG domain mating type protein of *S*. *macrospora* and *N*. *crassa* revealed the presence of a *MAT1–2–1* homolog (g12883.t1) in the genome sequence of *Calcarisporium sp*. The gene encodes a protein of 240 amino acids with a conserved HMG domain. Homology based extraction and explanation of these genes are provided in supplementary section 1 and in Figures S5 and S6. The *APN2* gene encoding a putative DNA lyase and the *SLA2* gene encoding a cytoskeleton assembly control factor have been reported neighboring *MAT* loci in many other ascomycetes^44^. Both genes, *APN2* (g12882.t1) and *SLA2* (g12886.t1) can be identified on the same contig_519 as the putative *MAT1–2–1* gene (Fig. 9). Two further open reading frames are adjacent to *MAT1–2–1* and flanked by *APN2* and *SLA2*, g12884.t1 and g12885.t1 on contig519 (Fig. 9), which encode proteins of 242 and 133 amino acids, respectively. Transcriptomic data revealed that both mating types genes are not expressed in wild type condition, while flanking genes - *APN2* (g12882.t1) and *SLA2* (g12886.t1) were expressed (Fig. 9), in at low level within tiers 9 and 8 (Table 2), respectively.

Taken together, the genome data showed that the sequenced isolate *Calcarisporium sp*. has a MAT1–2 mating type. MAT1–1 sequences could not be identified suggesting that *Calcarisporium sp*. is heterothallic, providing that *Calcarisporium spec* is able to reproduce sexually. A novel MAT1–2 specific ORF could be identified which is related to the MAT-1–2–3 protein encoded in the MAT1–2 loci members of the Hypocreales^45^. We performed similar homology-based detection of mating type genes in *Pestalotiopsis sp*., which yielded no results, suggesting that the isolated *Pestalotiopsis* strain has no mating type genes. We have recently reported that another marine fungus *S*. *brevicaulis* LF580 was a MAT1–1 strain^15^. These finding corroborates that reproduction in marine environments can be varied nature like either vegetative and sexually or both.

### Summary of carbohydrate active enzyme-encoding genes identified in the two novel marine fungal genomes

**Figure 10:**
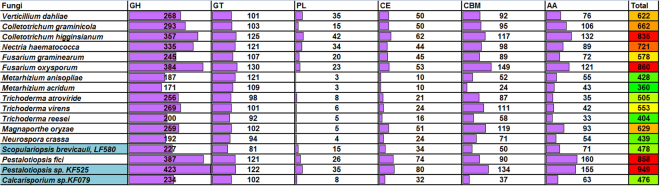
Summary of class-wise distribution of carbohydrate active enzymes in selected fungi. AA - auxiliary activities, GH - glycoside hydrolases, GT - glycosyltransferases, PL - polysaccharide lyases, CE - carbohydrate esterases and CBM - carbohydrate-binding modules.

Little is known about the lifestyle and ecological importance of marine fungi. Many water environments are low in nutrients and therefore unlikely to favor organisms that feed primarily by attachment to larger physical substrates and osmotrophy^2^. Fixed carbon on land is largely invested in the construction of large and complex plant tissues rich in energy and nutrients. Digestion of these plant tissues requires a complex set of carbohydrate active enzymes. Calcarisporium and Pestalotiopsis strains used in this study were collected in the German Wadden Sea, which is an area of about 3475 square miles with one of the highest biological primary productions in the world. As such the analysis of carbohydrate active enzymes is highly relevant. Using annotation tools derived from the CAZy database (http://www.cazy.org/), we identified 949 and 476 CAZy genes in *Pestalotiopsis sp*. KF079 and *Calcarisporium sp*. KF525 genomes (Fig. 10 and Table S10), respectively, confirming that *Pestalotiopsis* species are very rich in CAZymes and that *Calcarisporium sp*. is rather close in number to the marine derived- fungus *Scopulariopsis brevicaulis* LF580^46^. For *Pestalotiopsis sp*., the proteins encoded by the corresponding genome are divided into six major classes, namely 423 glycoside hydrolases (GH), 122 glycosyltransferases (GT), 35 polysaccharide lyases (PL), 80 carbohydrate esterases (CE), 134 carbohydrate binding module (CBM) and 155 auxiliary activities (AA). In comparison, CAZymes candidates of *Calcarisporium sp*. are radically lower in number for each class except for GT. Comparing the various genomes from mainly plant pathogens, entomopathogens and other model fungi, we can observe that the number of GTs, enzymes involved in the biosynthesis of oligo- and polysaccharides, is rather stable across genomes while the classes involved in the degradation processes (GH, PL, CE, CBM and AA) are highly variable and depend on the lifestyle of the fungus. The total number of CAZymes is generally high for plant pathogens and saprophytes, with the exception of *Trichoderma reesei*, known to produce a very efficient enzyme cocktail but poorly diversified. Inversely, the entomopathogenic fungi, *Metarhizium anisopliae* and *Metarhizium acridum*, are rather poor in all these enzyme classes. The two *Pestalotiopsis* species (marine strain and *P*. *fici*) examined belong to the fungal group harboring the highest CAZyme number along with the plant pathogens, *Colletotrichum higginsianum* and *Fusarium oxysporum*. *Calcarisporium sp*., together with *S*. *brevicaulis* are in an intermediate position as they have slightly lower numbers of GH, CE, CBM or AA members compared to all pathogic fungi and a very low content of PLs as for fungi possessing a specific life style, like *Trichoderma*, or *N*. *crassa*. Further details of Cazyomes of these two fungi are provided in supplementary section S2.

### Overview of MFS-type and sugar transporters encoded in the two novel genomes

**Figure 11:**
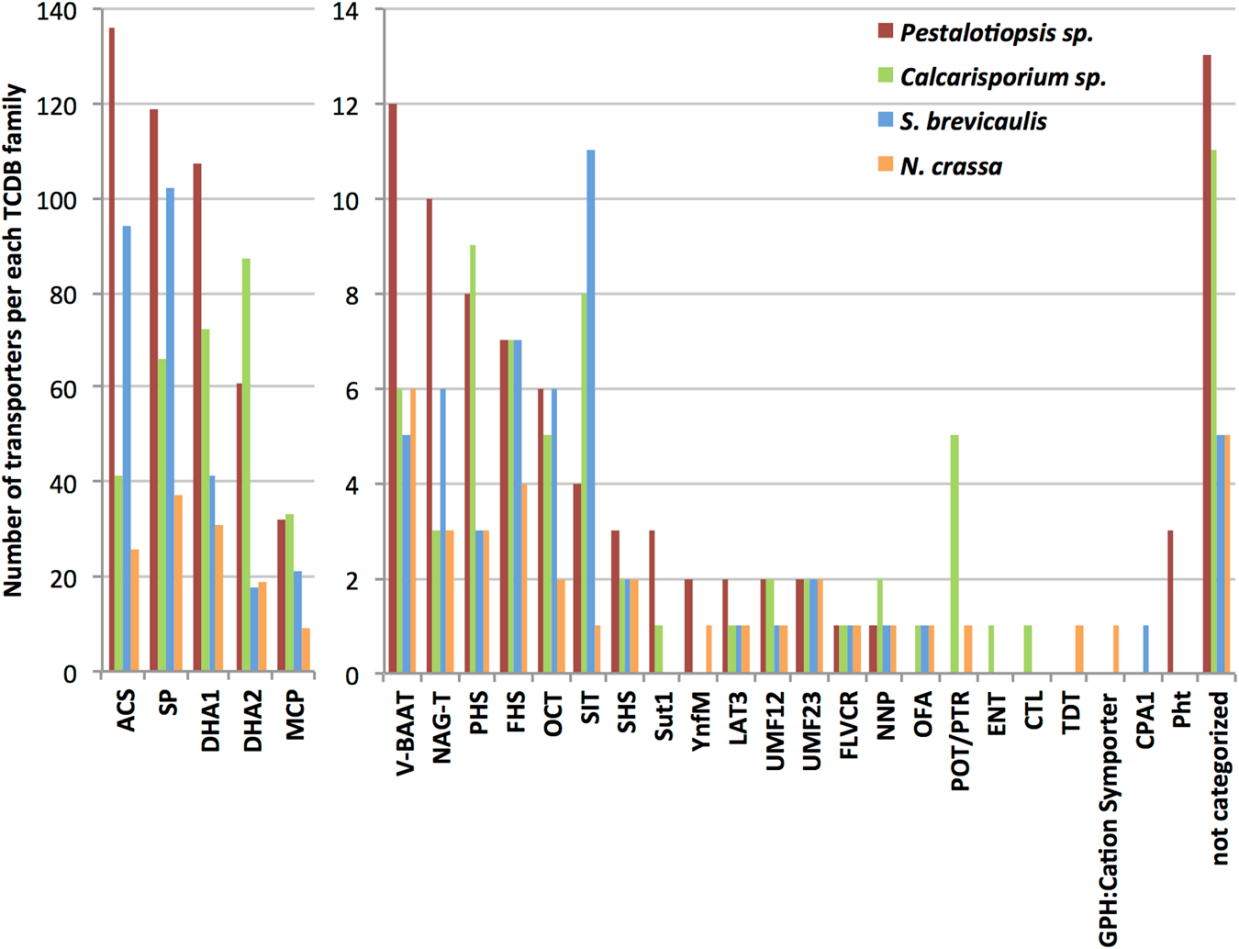
Comparative distribution of MFS-type and sugar transporter genes from *Pestalotiopsis sp*., *Calcarisporium sp*., *S*. *brevicaulis* and *N*. *crassa* into TCDB categories. The TCDB categories were ordered in descending fashion according to the number of *Pestalotiopsis* transporter genes present. The number of genes per category is presented as raw number for each organism. SP Family (TCDB category: 2.A.1.1); OFA Family (2.A.1.11); SHS Family (2.A.1.12); MCP Family (2.A.1.13); ACS Family (2.A.1.14); SIT Family (2.A.1.16); OCT Family (2.A.1.19); DHA1 Family (2.A.1.2); FLVCR Family (2.A.1.28); DHA2 Family (2.A.1.3); YnfM Family (2.A.1.36); LAT3 Family (2.A.1.44); V-BAAT Family (2.A.1.48); NAG-T Family (2.A.1.58); UMF12 Family (2.A.1.63); FHS Family (2.A.1.7); UMF23 Family (2.A.1.75); NNP Family (2.A.1.8); PHS Family (2.A.1.9); TDT Family (2.A.16); POT/PTR Family (2.A.17); GPH:Cation Symporter Family (2.A.2); CPA1 Family (2.A.36); Sut1 (2.A.2.6); ENT Family (2.A.57.5); CTL Family (2.A.92.1); Pht Family (2.A.1.53).

The genome sequence analysis of both *Calcarisporium sp*. and *Pestalotiopsis sp*. revealed that *Pestalotiopsis sp*. has the highest number of genes (534) encoding transport proteins predicted to have either a major facilitator superfamily (MFS) domain or a sugar transporter domain (Table S11). In *Calcarisporium* sp. on the other hand, the corresponding gene number (367) is rather close to the marine-derived *S*. *brevicaulis* strain LF580 (328 genes^15^). Comparing the distribution of the transporters into the categories defined by the Transporter Classification Database (TCDB; [2]), an overall similar distribution of the transporters of *Pestalotiopsis* sp., *S*. *brevicaulis* and *N*. *crassa* could be observed, while the distribution of *Calcarisporium* sp. transporters is slightly different (Fig. 11). We have provided complete details of transporter genes in the supplementary section S3.

### Summary of hydrophobins

**Figure 12:**
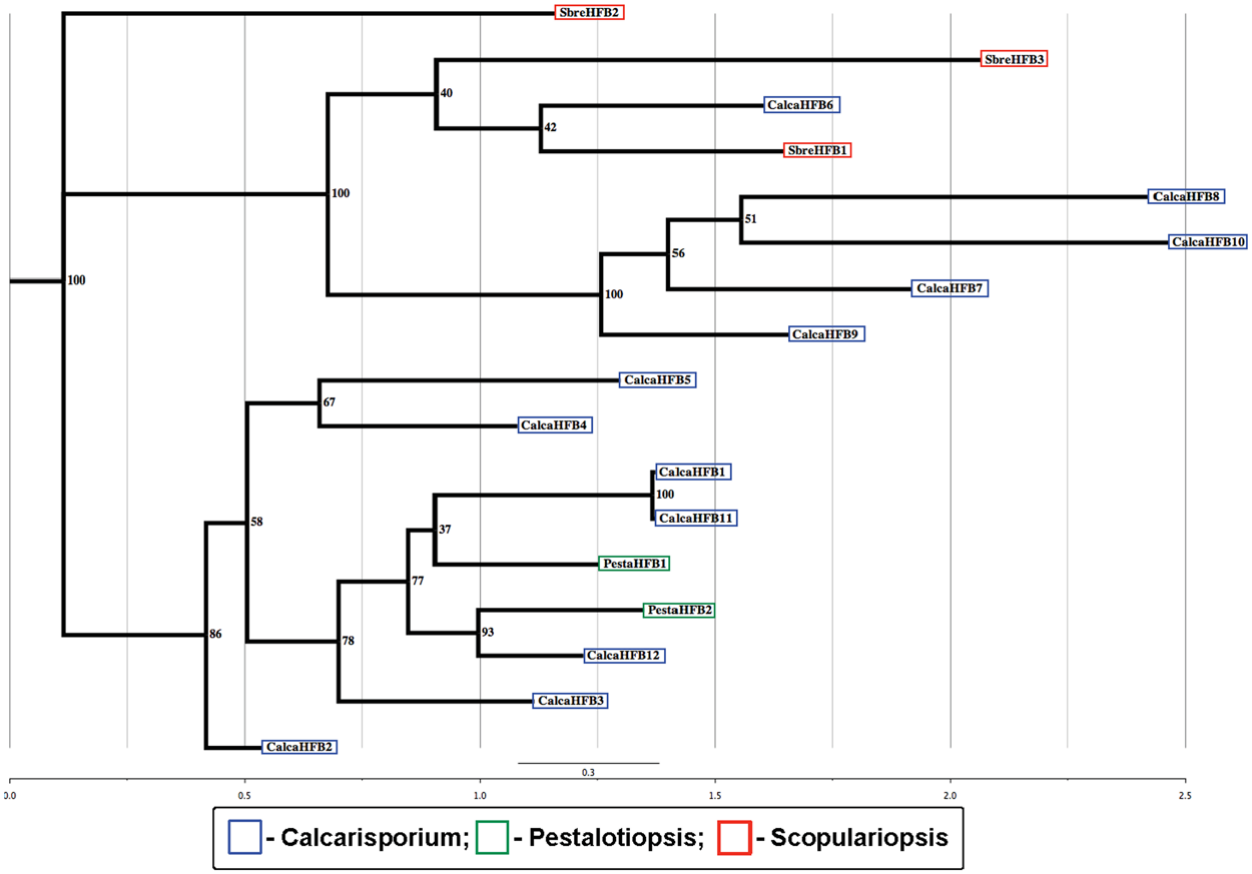
Phylogenetic analyses of hydrophins depict that there are different numbers of hydrophins are present in these marine fungi like 12, 2 and 3 in *Calcarisporium* sp. KF525, *Pestalotiopsis sp*. KF079 and *S*. *brevicaulis* KF580, respectively. Single copy of cryparin was found in two newly sequenced marine fungal genomes of *Calcarisporium* sp. KF525, and *Pestalotiopsis sp*. KF079.

Hydrophobins are morphogenetic, small mass (20 kDa) secreted hydrophobic fungi-specific cell wall proteins and they assist in the construction of aerial structures (like spores or fruiting bodies) in fungi [43]. The recently sequenced marine fungus *S*. *brevicaulis* LF580 possesses three hydrophobin genes, named as SbreHPB1 (g5510.t1), SbreHPB2 (g7216.t1), and SbreHPB3 (g15602.t1)^15^. *Calcarisporium* sp. and *Pestalotiopsis* sp. genomes were found to possess 12 and 2 hydrophobins, respectively (Fig. 12 and Table S12). Variable number of hydrophobins were also found in previous analyses of marine fungi^4^, and may represent adaptions to salt stress.

## Discussion

The oceans harbour enormous biochemical diversity in terms of natural products from various organisms. Marine fungi are potent producers of natural products however they have been under the least scientific focus. To bridge this gap, we used two marine fungal strains from the German Wadden Sea (*Calcarisporium* sp. KF525 and *Pestalotiopsis* sp. KF079) and performed genome wide scanning for the secondary metabolite genes and gene cluster (BGCs). We presented here genome sequences of two marine fungi namely *Calcarisporium* sp. KF525 and *Pestalotiopsis* sp. KF079 and the genome sizes of these two fungi are 35 Mb and ~46 Mb, respectively. Both genomes have a low content of total repeats (1%). Three other known marine fungal genomes, namely *S*. *brevicaulis* LF580^15^, *C*. *malorum* Mo12^21^ and *Cryptococcus* sp. Mo29^21^ have comparable repeat contents, which are close to 1%. This resides within the typical 1–4% of transposable elements in fungal genomes^20^. Exceptionally, only a few fungi have higher repeat contents like in a pezizomycete, *Tuber melanosporum* genome^47^ and several fungal genome of dothiodeomycetes^48^. However, these fungi typically have large expansions of the genome size like 125 Mb for *T*. *melanosporum*^47^.

Biosynthetic genes involved in the biosynthesis of a particular metabolite are organized in clusters and are often co-regulated and these clusters are known as biosynthetic gene clusters (BGCs)^19,49^. Exceptionally, more than one BGCs are involved in production of secondary metabolites like biosynthersis of meroterpenoids austinol and dehydroaustinol in Aspergillus nidulans^19^. These clusters have one or two core genes and several accessary genes, which encode proteins required for biosynthesis of the metabolite. Often filamentous fungi have 25 BCGs but this number can be much higher (80–90)^50^. The two marine fungi analysed here possess a total of 127 BGCs. Most of them have no significant homologies to BGCs in other fungi as well as in the bacteria. Notably, only three of these gene clusters are well characterized BGCs (Fig. 4) and reported in MiBiG datasets^28^. Our genome analyses thus revealed a huge amount of novel BGCs, highlighting that marine fungi are an exceptional source for secondary metabolites. Future studies should be aimed at identifying conditions in which the BGCs are active or methods to activate them artificially so that their properties can be analysed in more detail. In the draft fungal genomes, genomic fragments are smaller and hence in a few cases are noted that possesses a single gene in the cluster like mCaBGC60 (Figure S3). The cluster mCaBGC60 is localized on the contig1101, which is only 11.9 kb in size. Hence, drawing a conclusion from this cluster will be difficult, yet kept as found, and further genome assembly improvements and/or resequencing can resolve issues of such clusters.

*Pestalotiopsis* and *Calcarisporium* species belong to the Xylariales and Hypocreales, respectively, and are distributed in tropical and temperate regions (for a review on *Pestalotiopsis* see^51^). *Pestalotiopsis* species typically are phytopathogens causing different diseases i.e. needle or tip blights, canker lesions or fruit rots. *Pestalotiopsis* sp. are also found as endophytes or saprophytes, as they were isolated from dead leaves, barks and twigs. In addition, some species were found in animal infections demonstrating that these fungi could have versatile nutritional habits, or are opportunistic. In general, *Pestalotiopsis* sp. appears to be host-specific and live in a wide range of substrates or associated with different hosts. *Calcarisporium* sp. have a widespread occurrence and they are generally isolated as endophytes or parasites from basidiomycetes^52^, or ascomycetes, or from wood^53^, but have occasionally been isolated from marine environments^32^. In a recent study of a mangrove fungus, *Pestalotiopsis* sp. was described along with its adaptation to sea salt^54^. By a proteomic analysis, it was demonstrated that the lignocellulolytic enzyme composition was considerably changed in salt conditions, with for instance, a great reduction of the oxidase abundance, while specific carbohydrate-active enzymes are secreted exclusively at high salt concentrations. Taking into account all these data related to the CAZyome and sugar transporter repertoires (Tables S10–S11), it can be suggested that *Pestalotiopsis sp*. KF079, similar to the marine-derived *S*. *brevicaulis*, possesses a metabolism focused on the breakdown of the terrestrial plant biomass rather than algal or animal biomass. On the other hand, *Calcarisporium sp*. has adopted a different (and reduced) CAZy repertoire specialized for algae and animal degradation that reflects a life style oriented either towards parasitism or endophytic growth or towards utilization of dead algae or animals.

Upon scanning MAT loci in these two marine fungi, we demonstrated that *Calcarisporium sp*. has a MAT1-2 mating type while no MAT locus was detected in *Pestalotiopsis* sp.

Altogether, we present two draft genomes of marine fungi with a high number of BGC most of them being new clusters not observed before. These computational predictions of the genomic data provide further insight into genetics of these two fungi. This study step platform for the designing experiments to confirm findings about BGCs and corresponding secondary metabolite biosynthesis. We provide a comprehensive annotation of these genomes.

## Methods

### Fungal strain collection, cultivation, and DNA isolation

These two marine fungal strains (*Pestalotiopsis sp*. *KF079* and *Calcarisporium sp*. *KF525) were isolated from the German Wadden Sea*, which is the southeastern part of the North Sea. These strains were cultivated as previously described^55^. DNA isolation was per formed as described in the supplementary section S4.

### Genome sequencing, assembly, genome and RNA-Seq analyses

Short-read DNA sequencings were performed using Roche 454 and Illumina HiSeq™ 2000 methods with starting samples of 20 µg genomic DNA for these two marine fungi at Macrogen (Korea). We constructed hybrid *de novo* genome assemblies of Roche 454 and Illumina HiSeq™ 2000 for *Pestalotiopsis* sp. KF079 and *Calcarisporium* sp. KF525 using the Newbler assembler^56^ and the CLCBio Genomic workbench^57^, respectively. Further details of genomics and RNA-Seq analyses are provided in the supplementary section S4.

### Unraveling and characterization of biosynthetic gene clusters

Initially, putative genes that encoding for proteins which produce bioactive compounds are identified using BLAST^58^ with an E-value < 1e^−3^. Subsequently, this genome was analyzed using antiSMASH 3.0^29^ for putative clusters and further examined by manually coupled with RNA-Seq data. The functional domains of PKSs and NRPSs were identified as previously described^59^, using a combinations of tools namely antiSMASH 3.0^29^, NCBI Conserved Domain Database^60^, InterPro^24^ and the PKS/NRPS Analysis Web-site^61^.

### Data access

Entire datasets used in the current work were publically available using BioSample accession IDs: SAMN06272793 and SAMN06272794 with corresponding BioProject accession IDs as PRJNA368776 and PRJNA368777 for *Pestalotiopsis* sp. KF079 and *Calcarisporium* sp. KF525, respectively.

## Electronic supplementary material

Supplementary Information

TableS1

Table S2

Table S3

Table S4

Table S5

Table S6

Table S7

Table S8

Table S9

Table S10

Table S11

Table S12
