# Supplementary material for: Genome Sequencing and analyses of Two Marine Fungi from the North Sea Unraveled a Plethora of Novel Biosynthetic Gene Clusters
Source: Sci Rep. 2018 Jul 5;8:10187. doi: 10.1038/s41598-018-28473-z (PMC6033941; doi:10.1038/s41598-018-28473-z)
Supplement: Supplementary file 1 — Supplementary Information [file 41598_2018_28473_MOESM1_ESM.doc]

**Supplementary Information**

**Genome Sequencing and analyses of Two Marine Fungi from the North Sea Unraveled a Plethora of Novel Biosynthetic Gene Clusters**

**Abhishek Kumar^1^, Jens Laurids Sørensen^2^, Frederik Teilfeldt Hansen^3^, Mikko Arvas^4^, Muhammad Fahad Syed^4, 5^, Lara Hassan^6^, J. Philipp Benz^6^, Eric Record^7,8^,**

**Bernard Henrissat^8,9,10^, Stefanie Pöggeler^11^, and Frank Kempken^1, *^**

^1^Department of Genetics & Molecular Biology in Botany, Institute of Botany,

Christian-Albrechts-University at Kiel, Kiel, Germany

^2^Department of Chemistry and Bioscience, Aalborg University, Niels Bohrs Vej 8, DK-6700 Esbjerg, Denmark

^3^Department of Biochemistry, McGill University, Francesco Bellini Life Sciences Building, 3649 Promenade Sir William Osler, Montreal, QC, H3G 0B1, Canada

1. ^4^VTT Technical Research Centre of Finland Ltd, Tietotie 2, FI-02044 VTT, Espoo, Finland

^5^Biocomputing Platforms Ltd, Tekniikantie 14, FI-02150 Espoo, Finland

^6^Holzforschung München, TUM School of Life Sciences Weihenstephan, Technische Universität München, Hans-Carl-von-Carlowitz-Platz 2, Freising, Germany

^7^ INRA, Aix-Marseille Université, UMR1163, Biodiversité et Biotechnologie Fongiques, Marseille, France

^8^Centre National de la Recherche Scientifique (CNRS), UMR7257, Université Aix-Marseille, Marseille, 13288, France

^9^INRA, USC 1408 AFMB, F-13288 Marseille, France

^10^Department of Biological Sciences, King Abdulaziz University, Jeddah, Saudi Arabia

^11^Institute of Microbiology and Genetics, Department of Genetics of Eukaryotic Microorganisms, Georg-August University, Göttingen, Germany

***Corresponding author:**

**Frank Kempken,** Department of Genetics & Molecular Biology in Botany, Institute of Botany, Christian-Albrechts-University at Kiel, Kiel, Germany, [fkempken@bot.uni-kiel.de](mailto:fkempken@bot.uni-kiel.de)

**Email of other authors:**

AK – [abhishek.abhishekkumar@gmail.com](mailto:abhishek.abhishekkumar@gmail.com);

BH – [bernard.henrissat@afmb.univ-mrs.fr](mailto:bernard.henrissat@afmb.univ-mrs.fr);

ER – [eric.record@inra.fr](mailto:eric.record@inra.fr);

JLS – [jls@bio.aau.dk](mailto:jls@bio.aau.dk);

JFTH - [jens.hansen@mail.mcgill.ca](mailto:jens.hansen@mail.mcgill.ca);

JPB - [benz@hfm.tum.de](mailto:benz@hfm.tum.de);

LH - hassan@hfm.tum.de;

MA – [mikko.arvas@veripalvelu.fi](mailto:mikko.arvas@veripalvelu.fi);

MFS – [s_m_fahad@yahoo.com](mailto:s_m_fahad@yahoo.com);

SP – [spoegge@gwdg.de](mailto:spoegge@gwdg.de);

**Keywords**

Biosynthetic gene clusters; Calcarisporium; CAZyome; Marine fungi; Marine fungal genomics; Pestalotiopsis; Secondary metabolites; Transporters; Transcriptomics

# Supplementary section S1 – Overview of Mating locus in these two marine fungi

Reproduction in marine environments can be vegetative, sexually or both. In fungi, sexual reproduction is regulated by a genomic region referred to as the mating-type (*MAT*) locus ^1^. In self-sterile (heterothallic) species, mating occurs between morphologically identical partners that are only distinguished by their *MAT* locus. In filamentous ascomycetes the *MAT* locus consists of two dissimilar DNA sequences in the mating partners termed the *MAT1-1* and *MAT1-2* idiomorphs ^2^. In contrast to heterothallic species, the genome of self-fertile (homothallic) filamentous ascomycetes contains genes indicative of both mating types that can be either linked or unlinked ^3,4^.

The *MAT1-1* locus invariably contains the *MAT1-1-1* gene encoding a protein with a α-box domain. In most of the Sordariomycetes, two other genes are also located in the *MAT1-1* locus: (i) the *MAT1-1-2* gene encoding a protein with a PPF domain harbouring the three invariant residues proline (P), proline (P) and phenylalanine (F) and (ii) the *MAT1-1-3* gene encoding a protein with a high-mobility-group (HMG) domain as a DNA-binding motif. The characteristic of the *MAT1-2* locus is the *MAT1-2-1* gene encoding a protein with an HMG domain. In addition to the *MAT1-2-1* gene, other genes may also be present at the *MAT1-2* locus ^5^. Interestingly, even supposedly asexual filamentous fungi have been reported to contain functional, constitutively transcribed *MAT* genes ^6^.

A tBLASTN search with the MAT1-2-1 HMG domain mating type protein of *S. macrospora* and *N. crassa* revealed the presence of a *MAT1-2-1* homolog (g12883.t1) in the genome sequence of *Calcarisporium sp*. The gene encodes a protein of 240 amino acids with a conserved HMG domain. A Blastp search against the GenBank database showed the best hits in *Epichloe festucae* (accession AEI72619, E = 1e-111), the entomopathogenic fungus *Metarhizium acridum* CQMa 102 (accession EFY88585, E = 2e-110) and various species of *Trichoderma*, *Cordyceps* and *Fusarium* (Fig. S5).

Blast searches with MAT1-1 specific genes of *S. macrospora* and *N. crassa* yielded no hits.

Previously, in heterothallic and homothallic *Fusarium* species the MAT1-2 specific gene *MAT1-2-3* was identified directly adjacent to the *MAT1-2-1* gene ^7^. The N-terminus of the protein encoded by g12884.t1 revealed some sequence similarities with the MAT1-2-3 proteins of Fusarium species (Fig. S6), but the closest homolog is a hypothetical protein encoded by *M. acridum* CQMa 102 (accession EFY88586; E = 6e-22). A homolog of this gene is also present in genomes of *Trichoderma* species (*T. atroviride* E = 2e-17; *T. virens*, E = 2e-17, *T. reesei* 2e-13 and *Ophiocordyceps sinensis* 3e-11 (Fig. S6). All identified homologs correspond to a position immediately adjacent and in an inverse orientation to the mating type gene *MAT1-2-1*. BLASTP search with the133-aa protein encoded by g12885.t1 yielded no hits.

# Supplementary section S2 – Detailed Survey of carbohydrate active enzyme-encoding genes in two marine fungi

Using annotation tools derived from the CAZy database (<http://www.cazy.org/>), we identified 949 and 476 CAZy genes in *Pestalotiopsis* *sp.* KF079 and *Calcarisporium sp.* KF525 genomes (Figure 10 and Table S10) respectively, confirming that *Pestalotiopsis* species are very rich in CAZymes and that *Calcarisporium sp.* is rather close in number to the marine derived- fungus *Scopulariopsis brevicaulis* LF580 ^8^. For *Pestalotiopsis sp*., the proteins encoded by the corresponding genome are divided into six major classes, namely 423 glycoside hydrolases (GH), 122 glycosyltransferases (GT), 35 polysaccharide lyases (PL), 80 carbohydrate esterases (CE), 134 carbohydrate binding module (CBM) and 155 auxiliary activities (AA). In comparison, CAZymes candidates of *Calcarisporium sp.* are radically lower in number for each class except for GT. Comparing the various genomes from mainly plant pathogens, entomopathogens and other model fungi, we can observe that the number of GTs, enzymes involved in the biosynthesis of oligo- and polysaccharides, is rather stable across genomes while the classes involved in the degradation processes (GH, PL, CE, CBM and AA) are highly variable and depend on the lifestyle of the fungus. The total number of CAZymes is generally high for plant pathogens and saprophytes, with the exception of *Trichoderma reesei*, known to produce a very efficient enzyme cocktail but poorly diversified. Inversely, the entomopathogenic fungi, *Metarhizium anisopliae* and *Metarhizium acridum*, are rather poor in all these enzyme classes. The two *Pestalotiopsis* species (marine strain and *P. fici*) examined belong to the fungal group harboring the highest CAZyme number along with the plant pathogens, *Colletotrichum higginsianum* and *Fusarium oxysporum*. *Calcarisporium sp.*, together with *S. brevicaulis* are in an intermediate position as they have slightly lower numbers of GH, CE, CBM or AA members compared to all pathogic fungi and a very low content of PLs as for fungi possessing a specific life style, like *Trichoderma,* or *N. crassa*.

*Pestalotiopis* *sp.* KF079 contains the highest number of enzymes involved in the cellulose breakdown (families GH1, GH3, GH6, GH7, GH45, with the exception of GH12), with a general enzyme repartition in each class that is close to those of terrestrial fungi specialized in plant biomass modification or degradation. In comparison, *Calcarisporium sp.* is rather poor with only one or two members in each class with the exception of families GH3 and GH5 (11 and 12 candidates, respectively). Family GH5 is more difficult to interpret, as it is a highly multifunctional enzyme family. This family has been subdivided into subfamilies that show an improved correlation with activities such as β-glycosidases (subfamilies GH5_11, GH5_12 and GH5_22), endo-β-1,4-glucanases (GH5_5), endo-β-1,6-glucanases (GH5_15 and GH5_16) acting on polysaccharides from fungal cell walls, and xylanase (GH5_4) ^9^. Concerning family GH5, we observed that *Pestalotiopis sp.* KF079 has a subfamily profile similar to plant pathogens while the marine-derived fungi *S. brevicaulis*, and *Calcarisporium sp.* are less diversified, as it is the case for *Trichoderma* *or N. crassa*. On the other hand, the entomopathogenic fungi, which have a metabolism specialized in the insect material utilization, do not possess any cellulolytic GH6, GH7 and GH45 enzymes, and only one member of the GH12 family. For GH5, the differences are mainly related to the low number or absence of representatives of the families GH5_23 (hesperidin 6-O-α-L-rhamnosyl-β-glucosidase and hesperidin being involved in plant defenses), GH5_5 and GH5_7 (β-mannosidase and endo-β-1,4-mannanase).

For the hemicellulose degradation, *Pestalotiopsis* *sp.* KF079 harbors a rather important number of representatives of families GH10, GH11 and GH51, as expected for a plant degrading fungus, but it has the particularity to possess a high number of GH30 enzymes (eight representatives). Family GH30 groups together several different activities such as endo-β-1,4-xylanase, β-glucosidase, β-glucuronidase, β-xylosidase, and endo-β-1,6-galactanase. In contrast, only one member is represented for all these classes in the *Calcarisporium sp.* genome while the marine-derived *S. brevicaulis* is relatively close to the other terrestrial fungi. None of these xylan-acting families are present in the entomopathogenic fungi, and only one GH10 to three GH11 representatives are present in *T. reesei*. Xylanases are known to act in synergy with CEs, and *Pestalotiopsis* *sp.* is rich in CE1 enzymes with nine members, including at least four candidate acetyl xylan esterases and one candidate feruloyl esterase. The nine CE3 proteins from *Pestalotiopsis sp.* KF079 are putative acetyl xylan esterases (AXE). Sixteen family CE5 members were identified which correspond to five AXE and 11 cutinases. Other hemicellulases such as β-mannanases (GH26) are absent in *Pestaloptiopsis sp.* KF079. *Pestalotiopsis* *sp.* KF079 presents a high number of GH39 (four) candidate β-xylosidases, less represented in other plant degrading fungi. *Calcarisporium sp.* genome is poorly represented for xylan-acting families and has a comparable poor set for CE, i.e. CE1 (three), CE3 (six), and CE5 (11) with no GH26 nor GH39 members.

Because pectins have only been identified in land plants and the more recent streptophyte, and not in the early-diverging streptophyte algae ^10,11^, pectin-degrading enzymes have been suggested to be potential indicators for the association between fungi and land-plants lineage. Chang et al. (2015) showed that pectinases generally evolve rapidly ^12^. They can be lost by fungi that have adopted non-plant nutrition (yeast or fungi associated to animals) or their encoding genes duplicated while fungi have shifted to a plant-based nutrition and adapt the land plant diversification. During the transition from water to soil, early fungi may have moved to land by first living in microbial slime feeding on streptophyte algae in fresh-water rivers ^13^ and the age of the common ancestor of the terrestrial fungi was estimated to predate the origin of the land plants ^12^. In *Pestalotiopsis* sp., we were able to identify the main members of the pectinolytic enzymes, i.e. 13 PL1 members (candidate pectin/pectate lyase), seven PL3 enzymes (candidate pectate lyases), five PL4 and no PL11 members (candidate rhamnogalacturonan lyase), nine CE8 esterases (candidate pectin methylesterase), five CE12 proteins (candidate rhamnogalacturonan acetylesterase) and representatives of families GH28, GH53 and GH93. By comparison, plant pathogenic fungi and the marine-derived *S. brevicaulis* display a similar enzyme portfolio, while *Calcarisporium sp.* and *Trichoderma* species are characterized by a paucity or an absence of these enzyme families. The same picture could be drawn for the two enthomopathogenic fungi, *M. anisopliae* and *M. acridum*. Surprisingly, the PL7 family (alginate lyase), which contains enzymes involved in the degradation of the brown algae cell wall, is absent in *Pestalotiopsis* sp. or *S. brevicaulis*. No agarase, no carrageenase and no porphyranase (family GH16) were found in *Pestalotiopsis* *sp.* KF079 and *S. brevicaulis* or any of other terrestrial fungal genomes compared in our study, suggesting that *Pestalotiopsis sp.* is probably not adapted to the degradation of algal cell walls, as it was previously hypothesized for the marine derived *S.brevicaulis*. The *Calcarisporium sp.* genome was found to encode three members of the PL7 family and no member of the PL9 family, suggesting that this fungus may be adapted to the degradation of algal substrates. In addition, the entomopathogenic fungi, are characterized by the presence of GH84 (β-glucosaminidases) and GH89 (α-acethylglucosamidases) representatives, which are specifically involved in the degradation of animal glycans. No member of these families was found in the *Pestalotiopsis* sp. genome, suggesting also that it is not equipped for animal biomass degradation. In contrast, *Calcarisporium sp. KF525* genome contains one member for each family, suggesting that this fungus might also degrade animal glycans. The last interesting point on polysaccharide degradation concerns the lytic polysaccharide monoxygenase (LPMO), that oxidatively cleave the glycosidic bonds of the cellulose, chitin or starch (classified in families AA9, AA11 and AA13, respectively). With 27 LPMO genes, a number similar to what found in *Verticillium dahliae* and *C. higginsianum*, *Pestalotiopsis* *sp.* KF079 is again closer to plant pathogens than to other fungi that display one (*Metarhizium*) to five (*Calcarisporium sp.*) LMPOs genes.

The lignin degrading enzyme pattern of a fungus can give indications on whether it is able to utilize lignin-containing material. Auxiliary activities (AA) are requested to degrade or modify lignin and to give access to GH, but they also act on other aromatic compounds. If we consider *Pestalotiopsis* *sp.* KF079, its AA content is comparable to plant pathogen fungi AA. For instance, *Pestalotiosis* sp. there are 155 AA genes in the genome, similar to the 160 genes found in *P. fici*. The plant pathogens *Colletotrichum higginsianum* and *Fusarium oxysporum* have a slightly lower number with 132 and 121, respectively. Entomopathogenic fungi and *Trichoderma* species are rather poor for oxidative enzymes with around 40 representatives reflecting their nutrition mode. *Calcarisporium sp.* and *S. brevicaulis* possess 63 and 71 representatives, respectively. The most striking fact is that *Pestalotiopsis* sp. is largely represented by the multicopper family (AA1) with 26 representatives and the highest number of the AA3 (aryl alcohol oxidase, glucose oxidase) and AA5 (glyoxal oxidase, galactose oxidase), with 66 and 5 members for these families, respectively. In comparison, *Calcarisporium sp* has around 2 times less members for each family. AA3 and AA5 are provider of H_2_O_2_ that are used by peroxidase (AA2) and in the Fenton reaction to modify the lignin and/or the cellulose.

For instance, while *Calcarisporium* sp. has less Sugar Porter family (2.A.1.1) transporters than *Pestalotiopsis* sp. and *S. brevicaulis*, it has considerably more representatives of the Drug:H^+^ Antiporter family 2 (DHA2; 2.A.1.3). Also, *Calcarisporium* sp. seems more diversified, having 1-5 transporters of the Equilibrative Nucleoside Transporter family (ENT; 2.A.57.5), the Choline Transporter-like family (CTL; 2.A.92.1) and the Proton-dependent Oligopeptide Transporter family (POT/PTR; 2.A.17), which are absent in the *Pestalotiopsis* sp. and *S. brevicaulis* genomes. *Calcarisporium* and *S. brevicaulis* furthermore have a particularly high number of transporters of the Siderophore-Iron Transporter family (SIT Family 2.A.1.16)*.*

# Supplementary section S3 – Detailed overview of MFS-type and sugar transporters encoded in the two marine fungal genomes

An overview of MSF-type and sugar transporters is provided here (**Figure 11**).

Similar to *S. brevicaulis* and *N. crassa*, the major categories in both *Calcarisporium* sp. and *Pestalotiopsis* sp. are the Anion:Cation Symporter family (ACS; 2.A.1.14), the Sugar Porter family (SP; 2.A.1.1), the Drug:H^+^ Antiporter families 1 and 2 (DHA1; 2.A.1.2 and DHA2; 2.A.1.3), as well as the Monocarboxylate Porter family (MCP; 2.A.1.13) (**Supplementary Table S11**). Considering the transporters assigned to sugar transport (2.A.1.1), *Pestalotiopsis* has the highest number of transporter genes belonging to this family (119) which is, in number, similar to *S. brevicaulis* (102), indicating a high sugar transport capacity of *Pestalotiopsis*. Moreover, it suggests that its sugar transporters may be more functionally diverse and thus able to utilize a greater variety of different sugars, which would coincide with the overrepresentation of CAZy genes in the *Pestalotiopsis* genome. On the other hand, *Calcarisporium* possesses only about half as many sugar transporters (66 genes belonging to 2.A.1.1), yet still about twice as much as *N. crassa* (37 genes) in the same category [1].

Using the better annotated transporters of *N. crassa* [3-13]*,* we attempted to broadly assign the MFS-type transporters from *Pestalotiopsis* sp. and *Calcarisporium* sp. belonging to the Sugar Porter family (2.A.1.1) to probable substrate groups by phylogenetic analysis. The transporters of both strains clustered into six main categories: pentose transporters, cellodextrin/β-linked hexose transporters, glucose/hexose transporters, maltose/α-linked hexose transporters, uronic acid/carboxylic acid transporters, and a group of transporters of so far unclear function (**Figures S7 and S8**). Comparing the number of transporters in each category, we noticed an expansion of *Pestalotiopsis* sp. and *Calcarisporium* sp. transporters in the cellodextrin/β-linked hexose transporter category and the uronic acid/carboxylic acid transporter category. Surprisingly, the opposite was found for the glucose/hexose transporters, where the relative fraction of transporters in *Pestalotiopsis* sp. and *Calcarisporium* sp. was less than in *N. crassa*. Moreover, while *Pestalotiopsis* sp. seems enriched in the cellodextrin/β-linked hexose transporter category compared to *N. crassa*, this does not seem to be the case for *Calcarisporium* sp.

The phylogenetic correlation of *Pestalotiopsis* sp. and *Calcarisporium* sp. transporters with representatives of known function provides a first rough insight into the possible substrate classes of the encoded transport proteins. However, during the adaptation to a drastically different lifestyle, the transporters clearly diversified and likely evolved towards novel substrates, which remain to be identified.

# Supplementary section S4 – Detailed Methods

## S4.1. Collection of fungal strain and cultivation

1. These two marine fungal strains (*Pestalotiopsis sp. KF079* and *Calcarisporium sp. KF525) were isolated from the German Wadden Sea,* which is the southeastern part of the North Sea. These strains were cultivated as previously described ^14^. These strain were received from the fungal collection of the Kiel Center for Marine Natural Products as cryo-conserved material. These marine fungi were cultivated on solid WSP30 medium, which is a variant of Wickerham-medium (with composition as following 1% glucose, 0.5% soy peptone, 0.3% malt extract, 0.3% yeast extract, 3% NaCl) ^15^.
2. **S4.2. DNA isolation**
3. Whole genome DNA samples from these two marine fungi were prepared by following a modification of previously published methods ^16,17^. Mycelium was frozen in liquid nitrogen, pulverized, and incubated in equal volumes of lysis buffer (10 mM Tris-HCl, 1 mM EDTA, 100 mM NaCl, 2% SDS, pH 8.0), After centrifugation, the supernatant was treated with RNase, and afterwards with an equal volume phenol/chloroform (1:1).

## S4.3. Genome sequencing and data availability

1. Short-read DNA sequencings were performed using Roche 454 and Illumina HiSeq™ 2000 methods with starting samples of 20 µg genomic DNA for these two marine fungi at Macrogen (Korea). Entire datasets used in the current work were publically available using BioSample accession IDs: SAMN06272793 and SAMN06272794 with corresponding BioProject accession IDs as PRJNA368776 and PRJNA368777 for *Pestalotiopsis* sp. KF079 and *Calcarisporium* sp. KF525, respectively.

## S4.4. Genome assembly and repeats detection

We constructed hybrid *de novo* genome assemblies of Roche 454 and Illumina HiSeq™ 2000 for *Pestalotiopsis* sp. KF079 and *Calcarisporium* sp. KF525 using the Newbler assembler ^18^ and the CLCBio Genomic workbench ^19^, respectively.

1. We deduced repeat elements of these two fungal genomes with help of Repeat Masker and RepeatProteinMasker tools (Smit, AFA, Hubley, R & Green, P. RepeatMasker Open-4.0.0 1996–2013 [http://www.repeatmasker.org](http://www.repeatmasker.org/)) using the fungal transposon species library (database version 20120418).

## S4.5. Gene prediction and annotation analyses

1. We predicted estimated genes using Augustus package using *Asperigillus niger* as training dataset. We carried out the gene annotation using BLAST homology searches ^20^ with an E-value cutoff of 1e−3 within BLAST2GO suite ^21^. Additionally, predicted proteins were annotated using BLAST ^20^ with comparing the Kyoto Encyclopedia of Genes and Genomes (KEGG) ^22^, Swiss-Prot, TrEMBL, Gene Ontology (GO), and non-redundant (NR) databases.
2. Predicted proteins of this genome were scanned to all known Pfam (version 27) ^23^ and Interpro (version 43) ^24^ protein domains collections, respectively using HMMER 3.0 ^25^, as described previously ^26^. In order to confirm the phylogenetic position of these marine species, we also performed a phylogenomic analysis using the CVtree ^27^, as described recently ^26^.

## S4.6. RNA isolation and RNA-Seq analyses

1. Two fungal strains were grown on the WSP-30 medium for 7 days at 200 rpm

in the dark. RNA was isolated using previously known methods for RNA isolation ^16,28,29^. RNA sequencing was performed using Illumina HiSeq™ 2000 at the Beijing Genome Institute (BGI) (Shenzhen, China). Raw reads were mapped to predicted genes using RNA-Seq mapping tool of CLCBio Genomic workbench ^19^ and relative expression levels were measured as Reads Per Kilobase of transcript per Million mapped reads (RPKM).

## S4.7. Detection of CAZymes

1. We compared all putative proteins against the entries in the CAZy database
2. ^30^ using BLASTP ^20^, as previously ^26^.

## S4.8. Surveying and characterization of MFS-type and sugar transporters

1. MFS-type or Sugar Transporter-type genes from two the fungal genomes were identified by Pfam scanning and performing sequence similarity searches against the Transporter Classification Database (TCDB) ^31^ as described recently ^26^. Phylogenetic trees were created using protein sequences from *Calcarisporium sp.* and *Pestalotiopsis* sp. MFS-type transporters belonging to the Sugar Porter sub-family (SP; 2.A.1.1) with transporters from *N. crassa.* The sequence alignments and tree generation were performed using MUSCLE ^32^ and PhyML programs ^33^, respectively. iTOL was used for tree visualization and editing ^34^

**References:**

Fig. S1: Overview of genome sizes and gene contents in selected fungi.


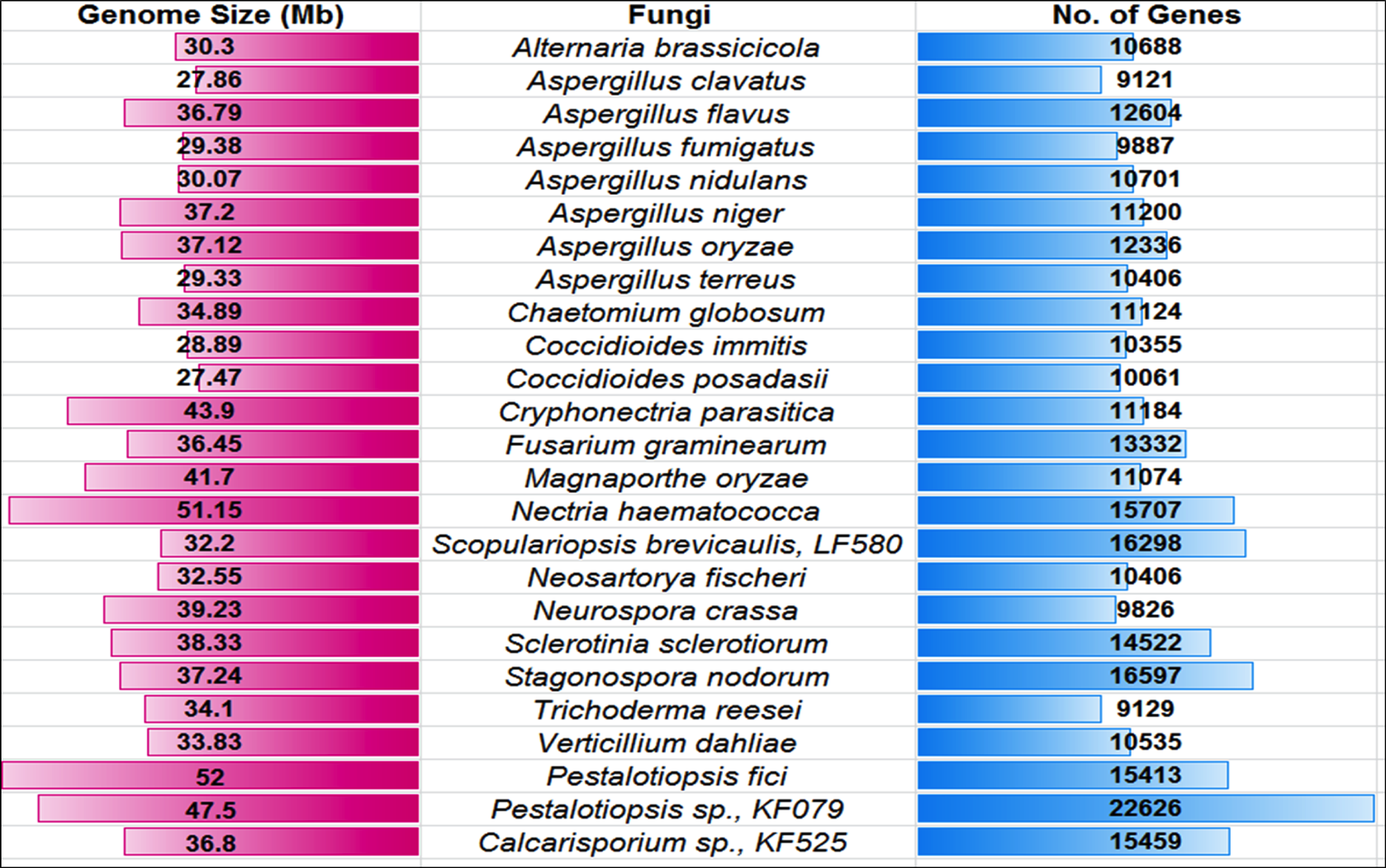


Fig. S2: Phylogenetic distribution of fungi


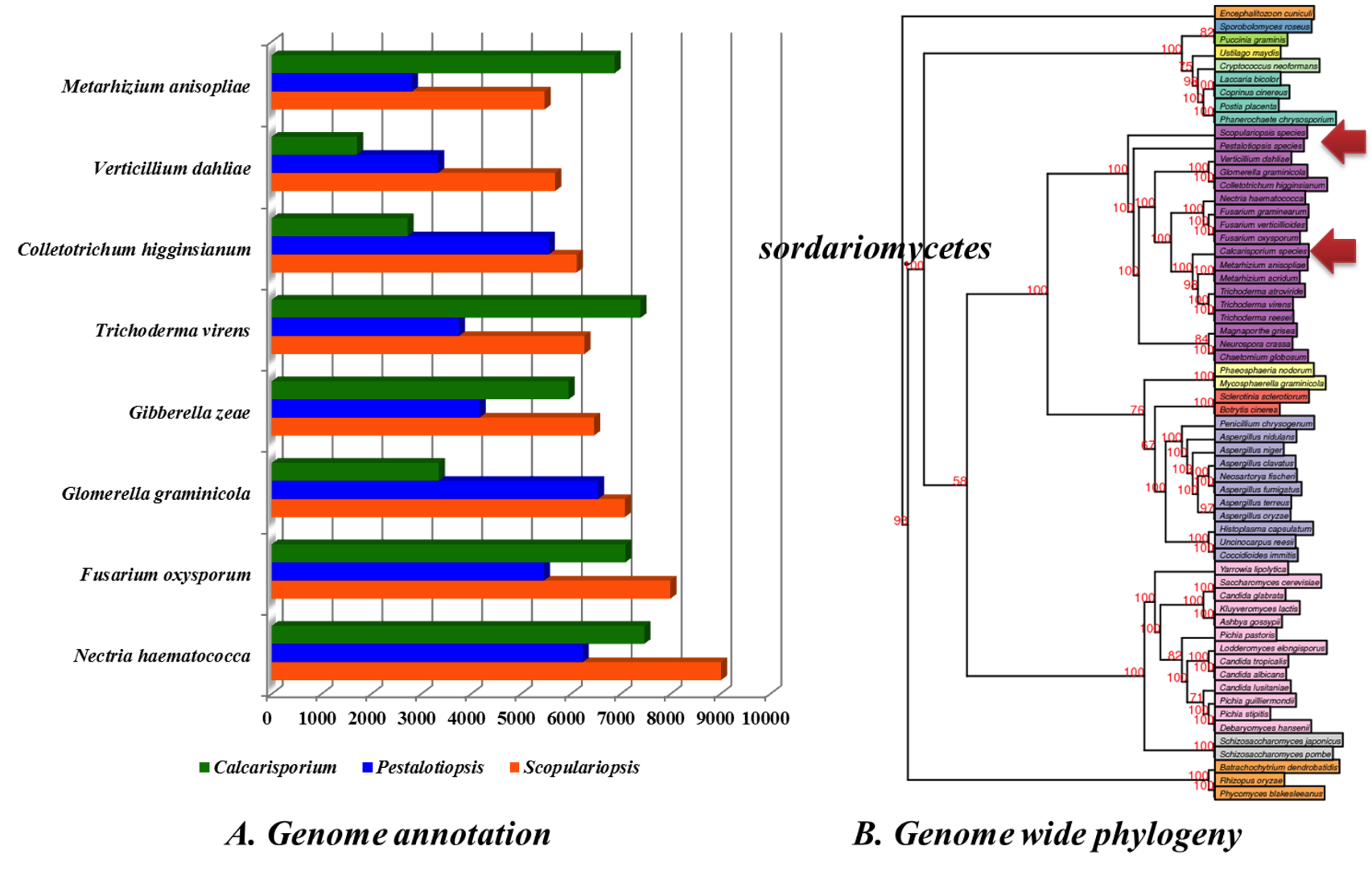


Fig. S3: Overview of 60 BGCs of *Calcarisporium* sp., KF525 genome with details of homologous clusters in other organisms.

1. **mCaBGC1 - contig_10 – Other**


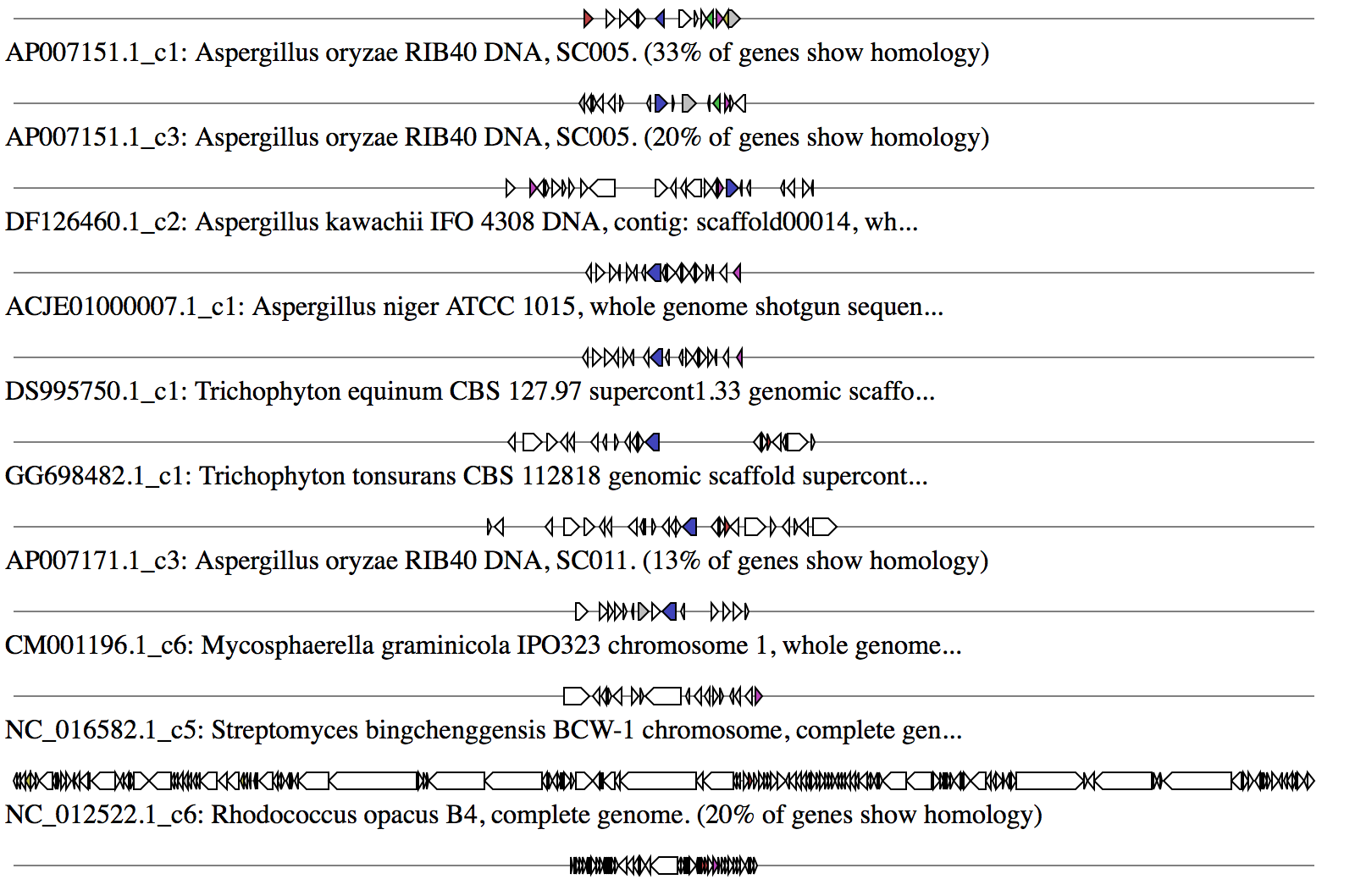


1. **mCaBGC2 - contig_39 - Nrps-t1pks**


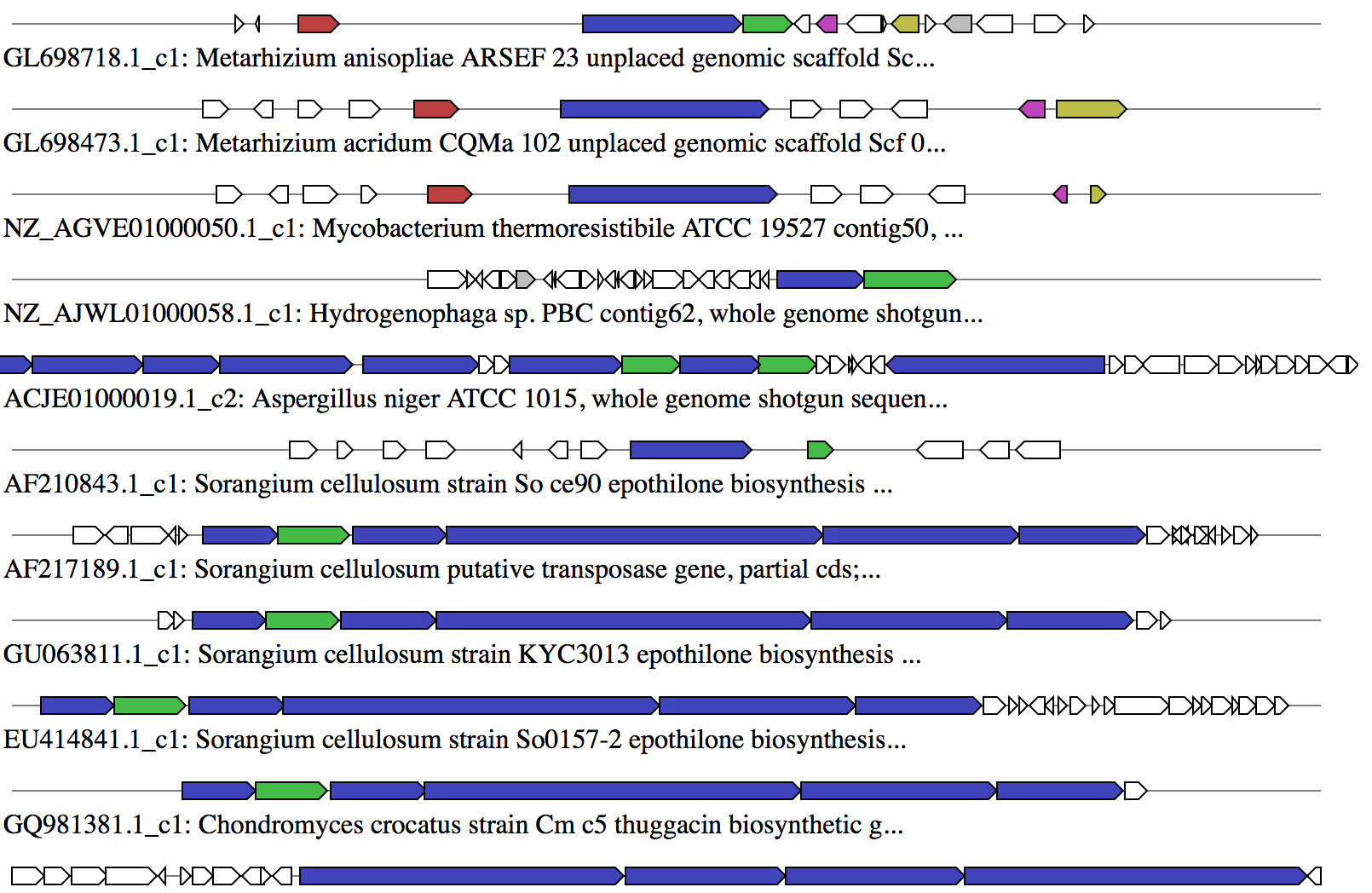


1. **mCaBGC3 - contig_44 – Nrps**

**
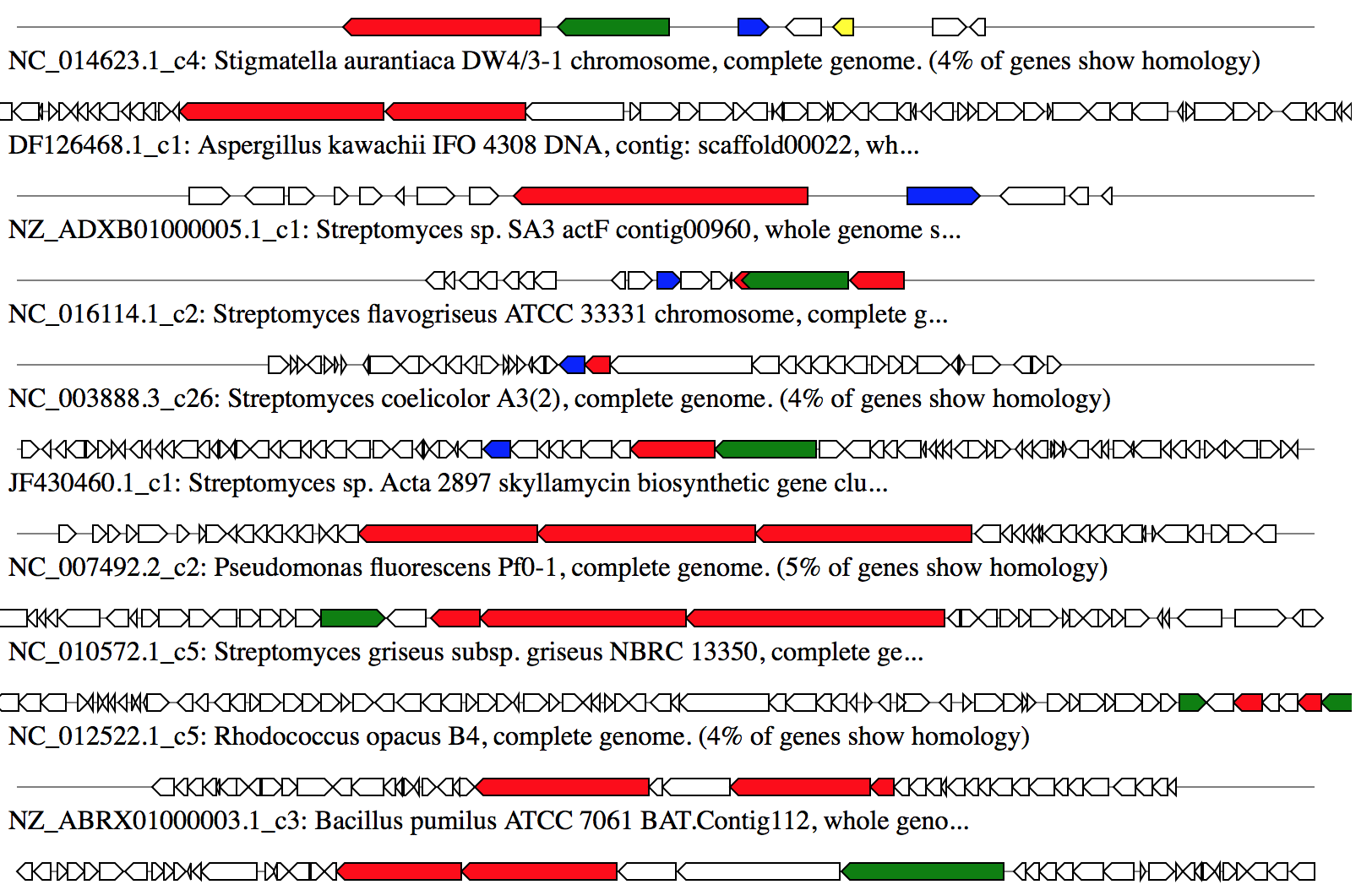
**

1. **mCaBGC4 - contig_59 - Hglks-t1pks**

**
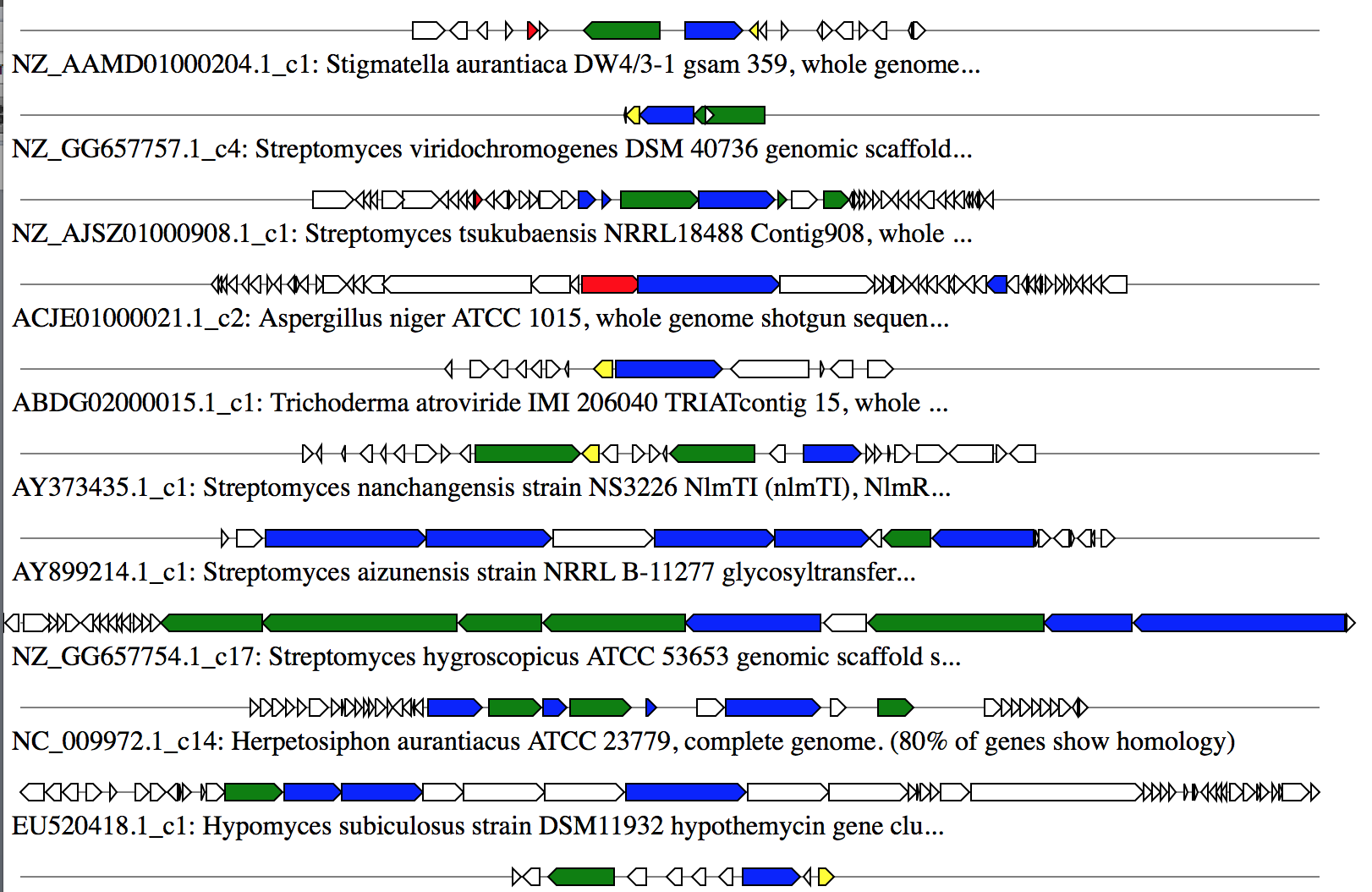
**

1. **mCaBGC5 - contig_65 – Nrps**

**
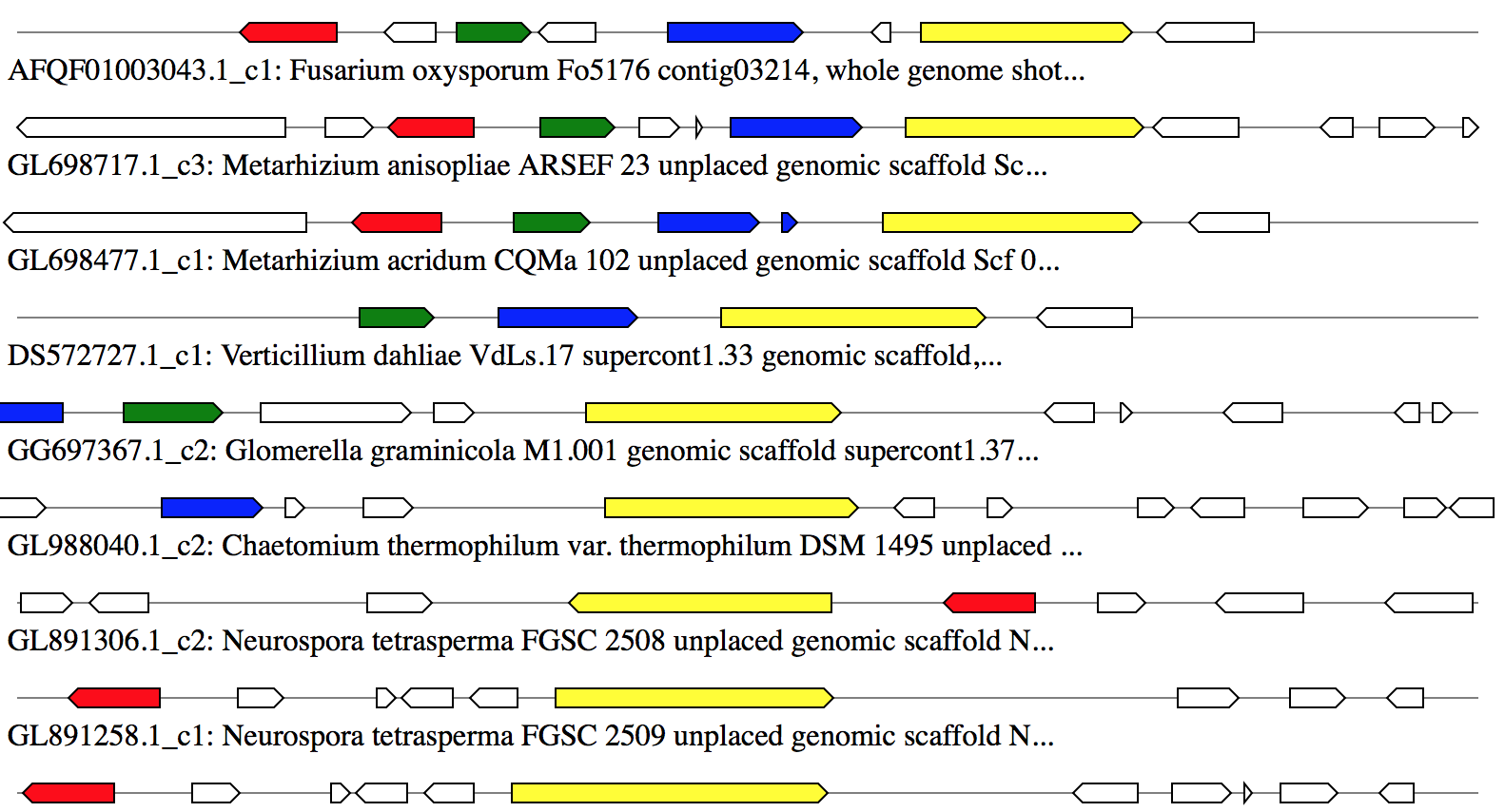
**

1. **mCaBGC6 - contig_72 – Terpene**

**
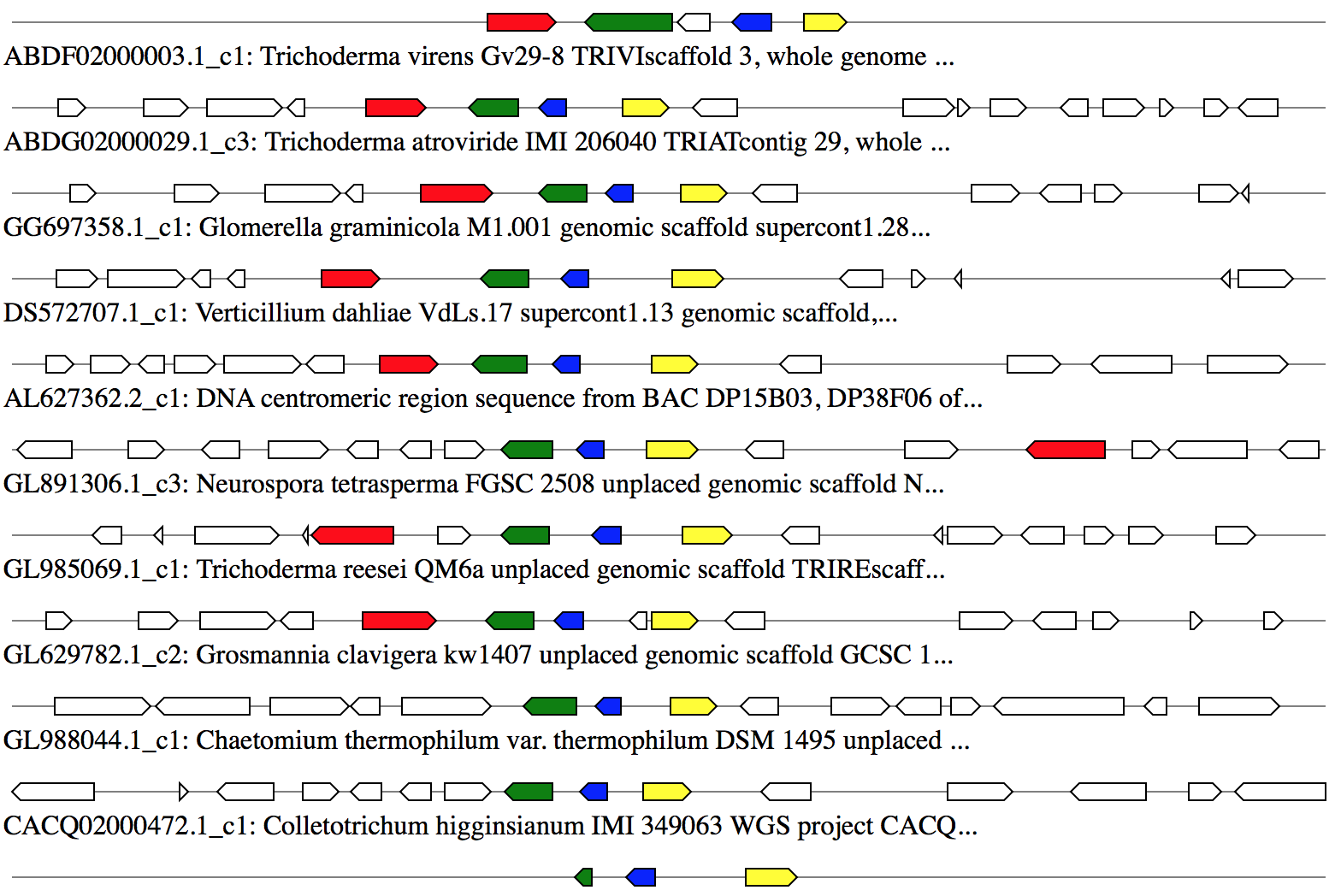
**

1. **mCaBGC7 - contig_73 - T1pks**

**
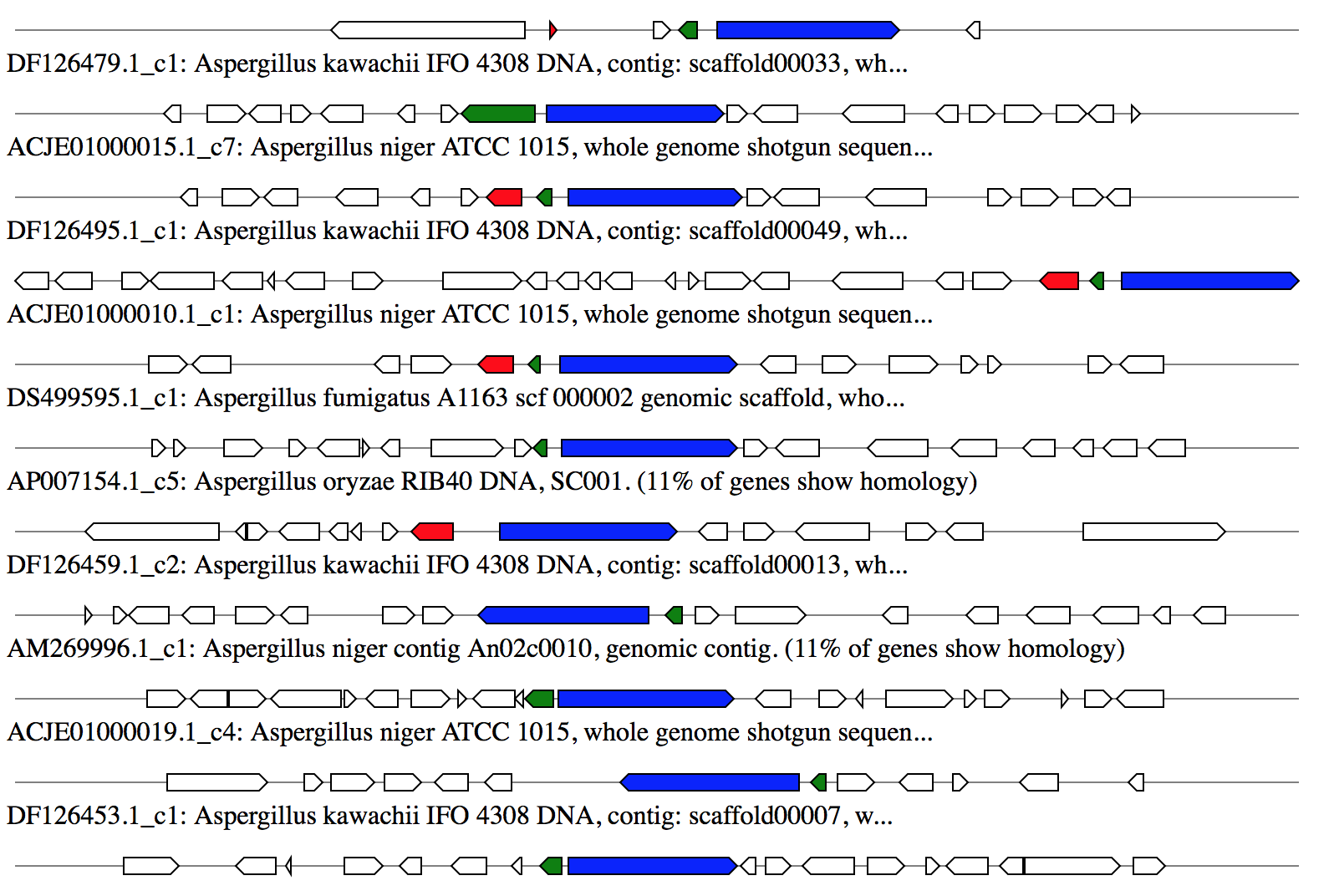
**

1. **mCaBGC8 - contig_81 – Nrps**

**
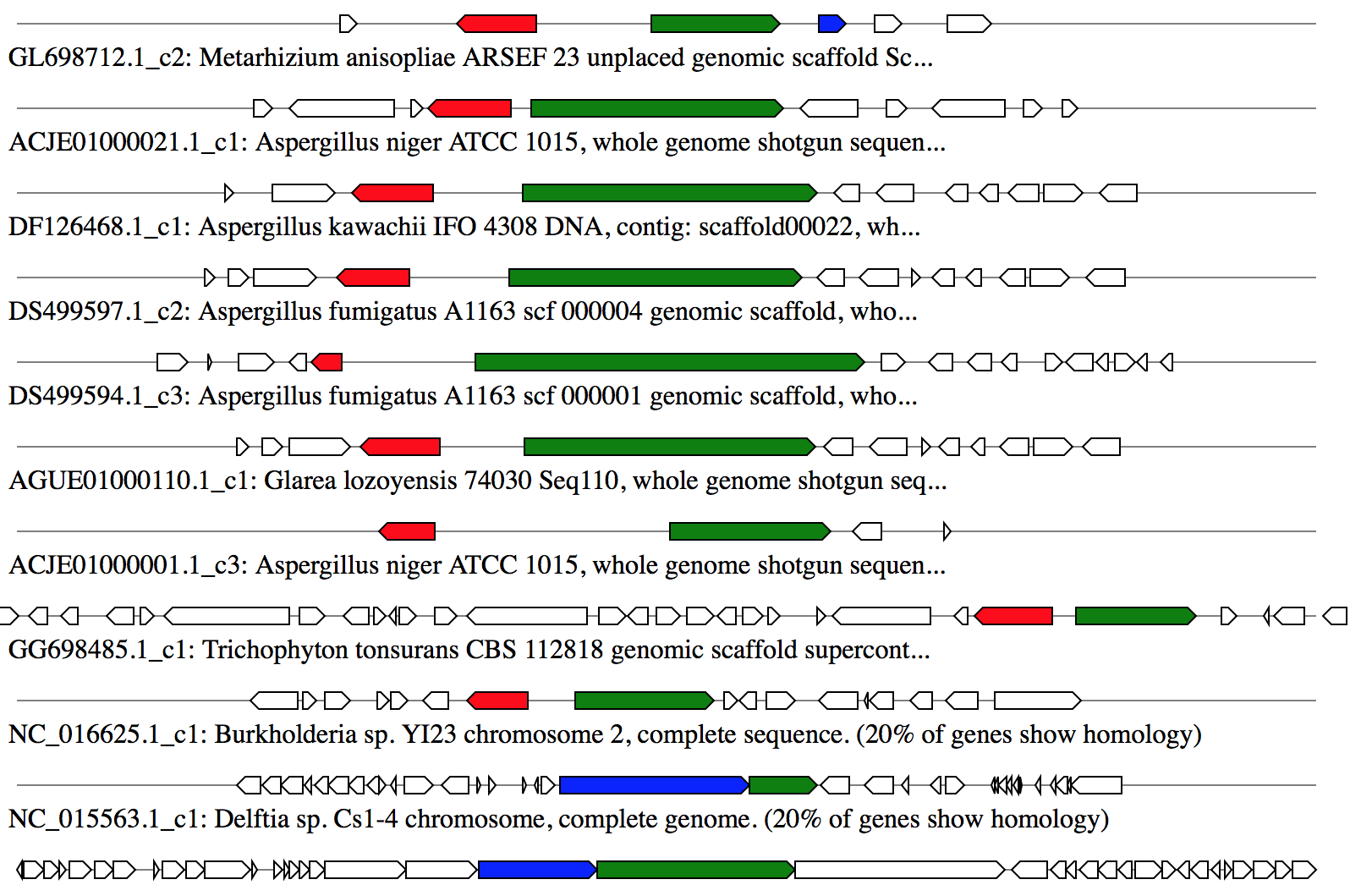
**

1. **mCaBGC9 - contig_85 - T1pks**

**
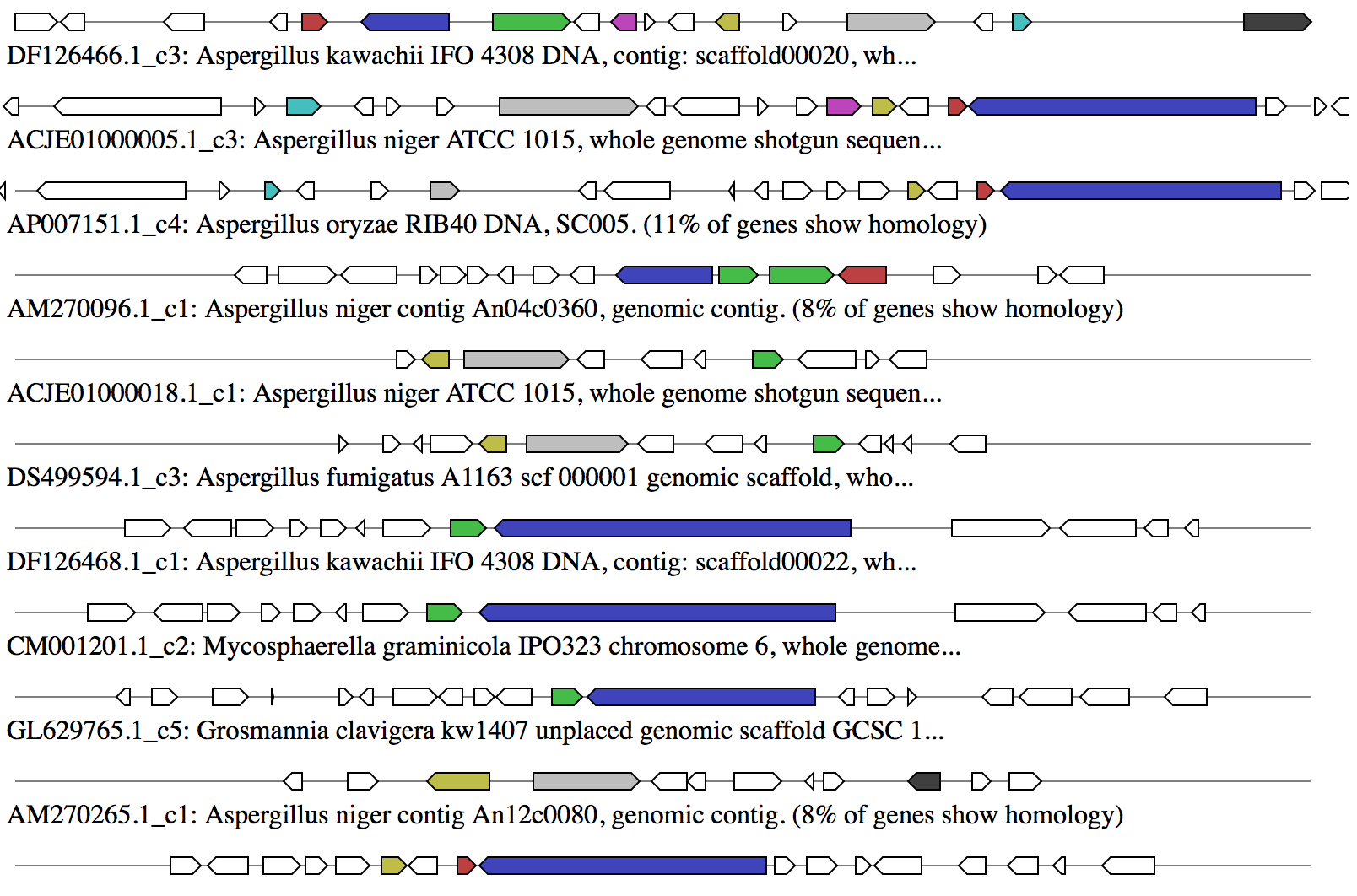
**

1. **mCaBGC10 - contig_100 - T1pks**

**
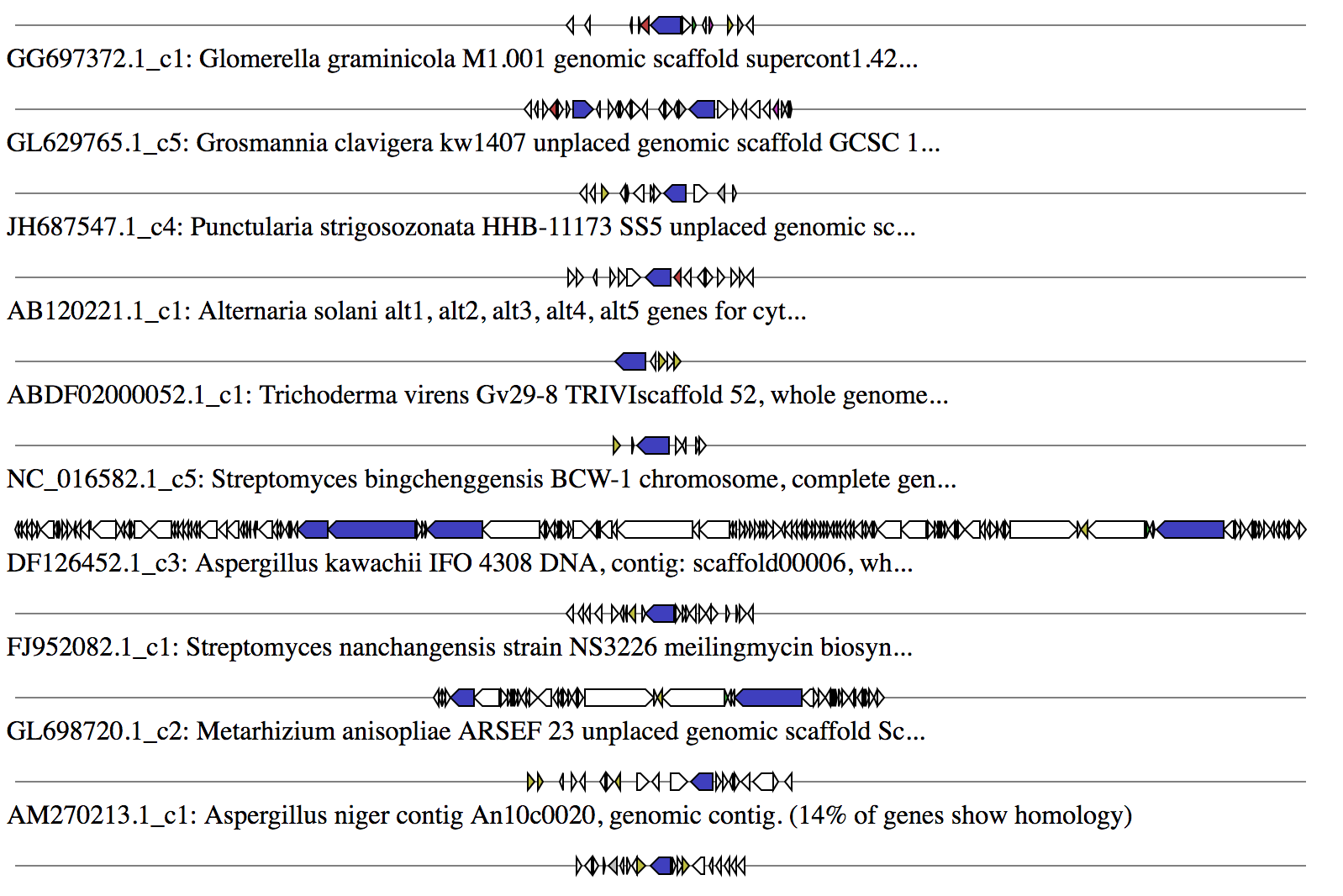
**

1. **mCaBGC11 - contig_101 - T1pks**

**
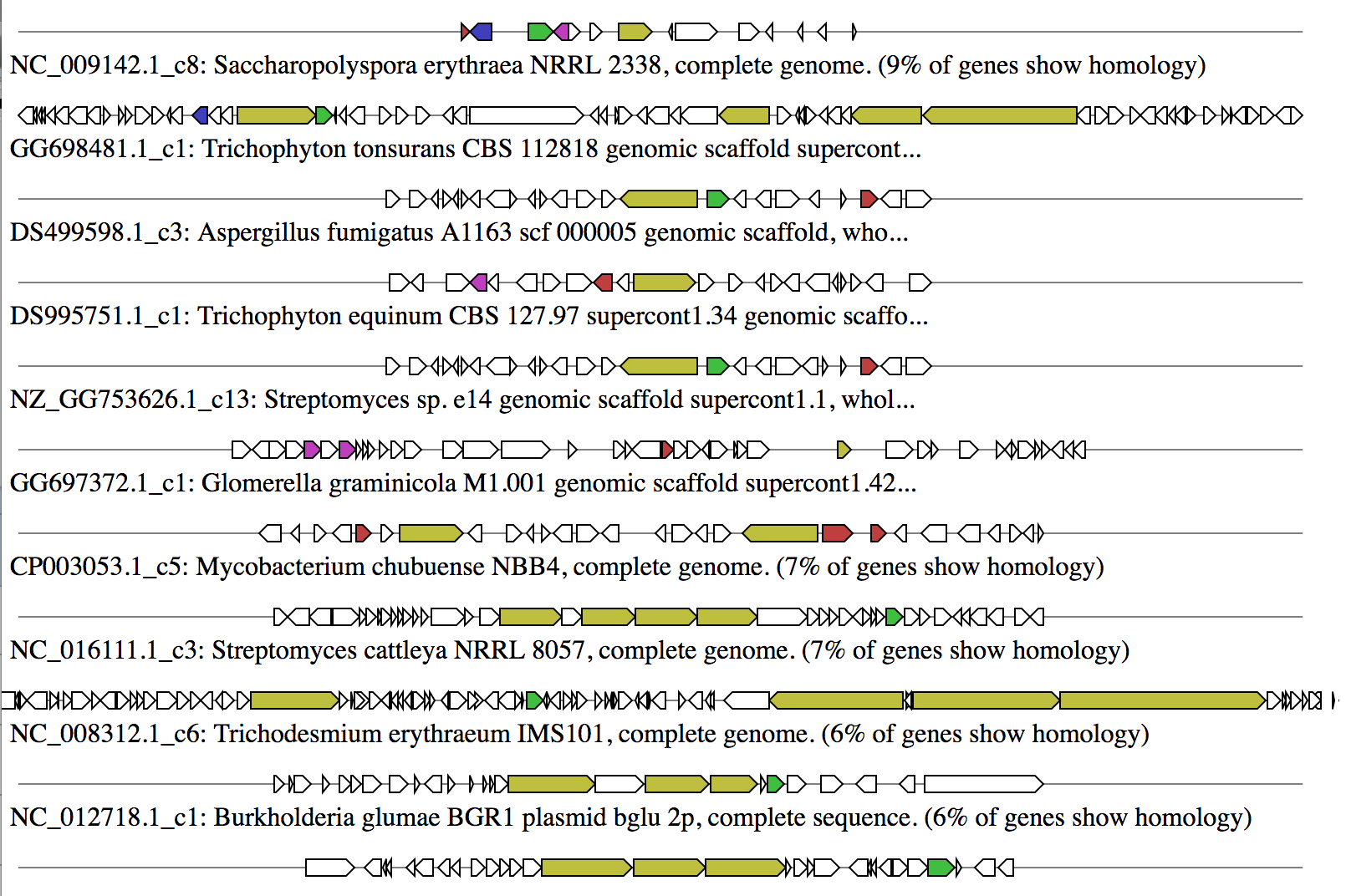
**

1. **mCaBGC12 - contig_101 – Terpene**

**
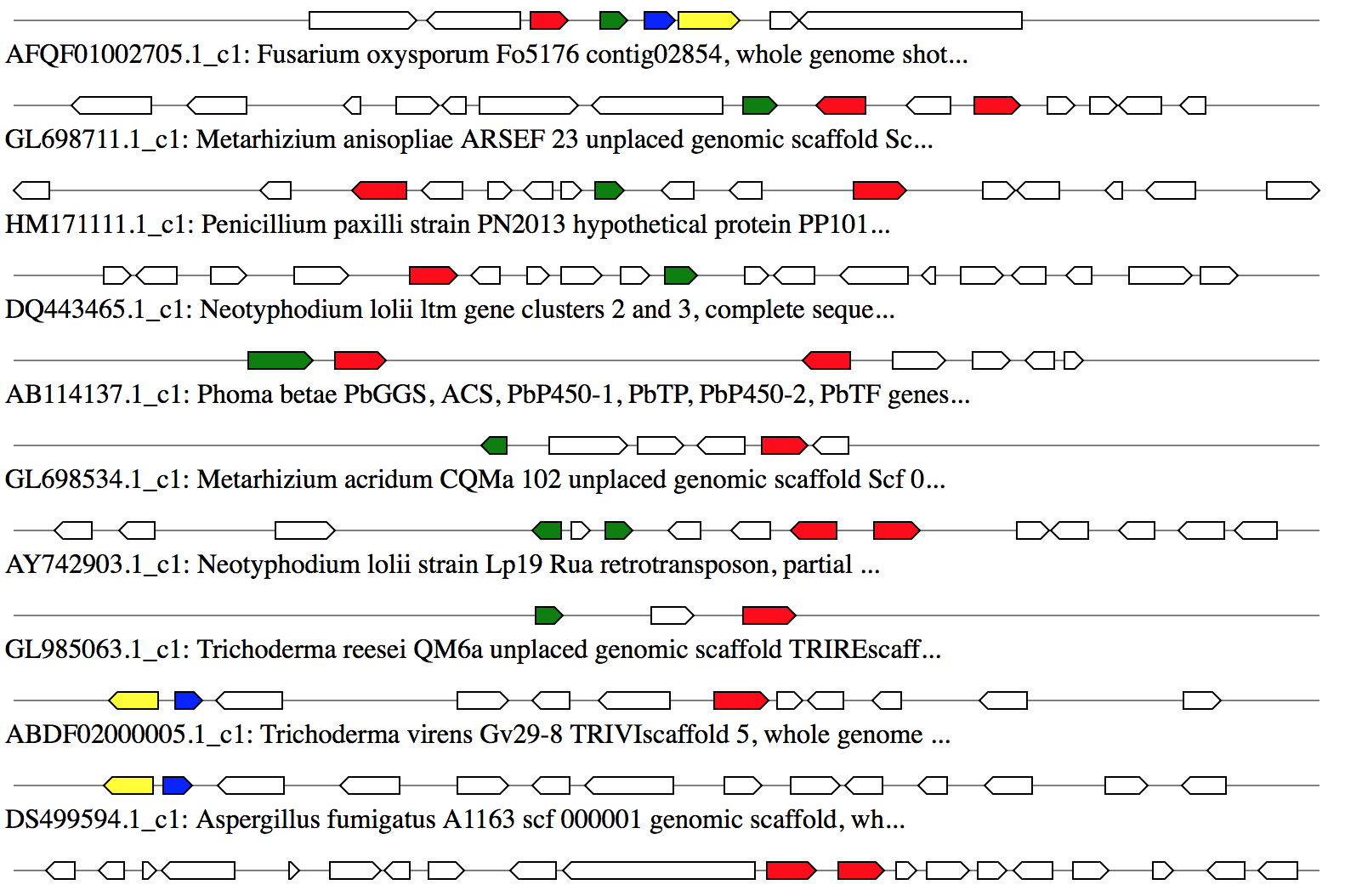
**

1. **mCaBGC13 - contig_116 - T1pks**

**
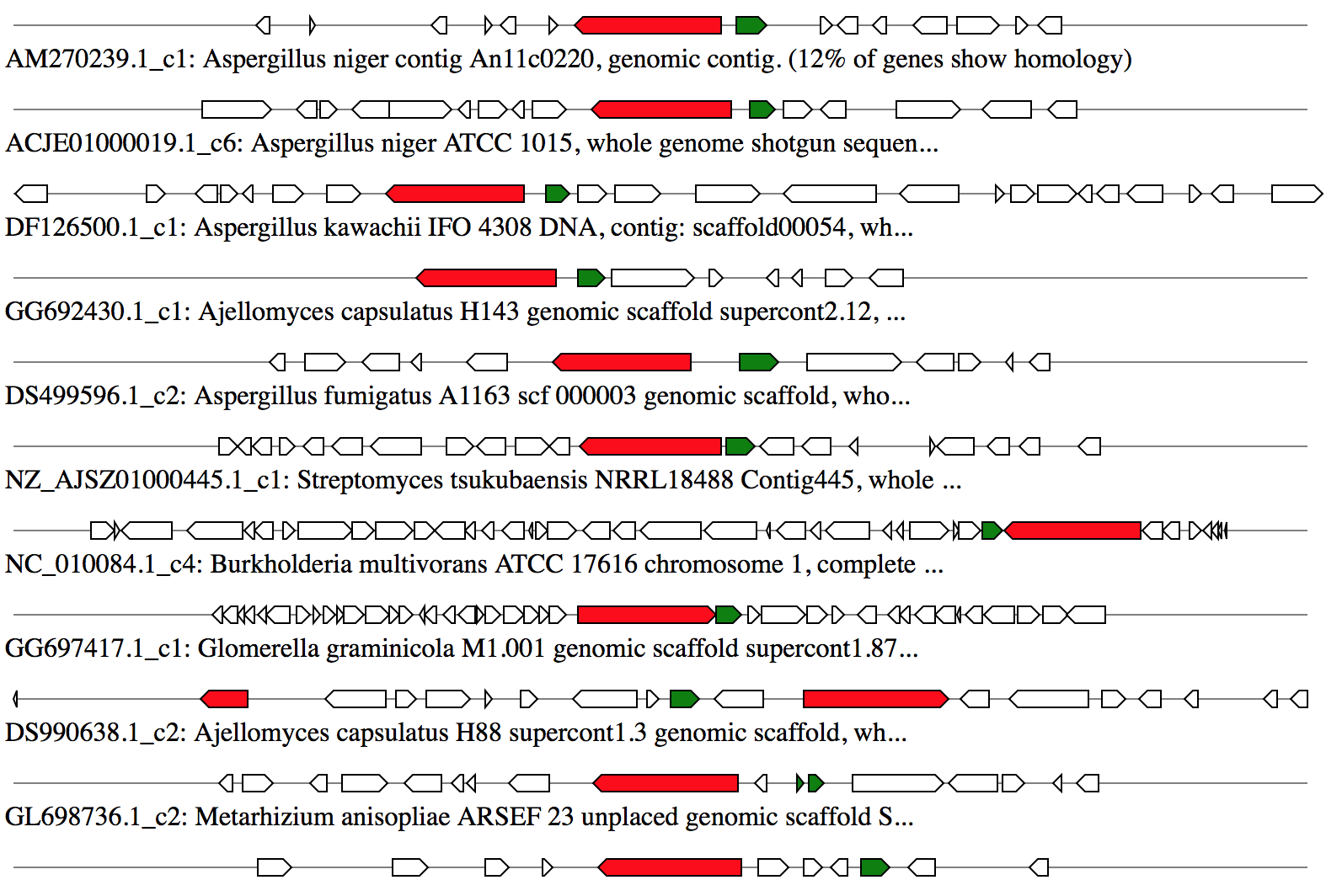
**

1. **mCaBGC14 - contig_123 – Other**

**
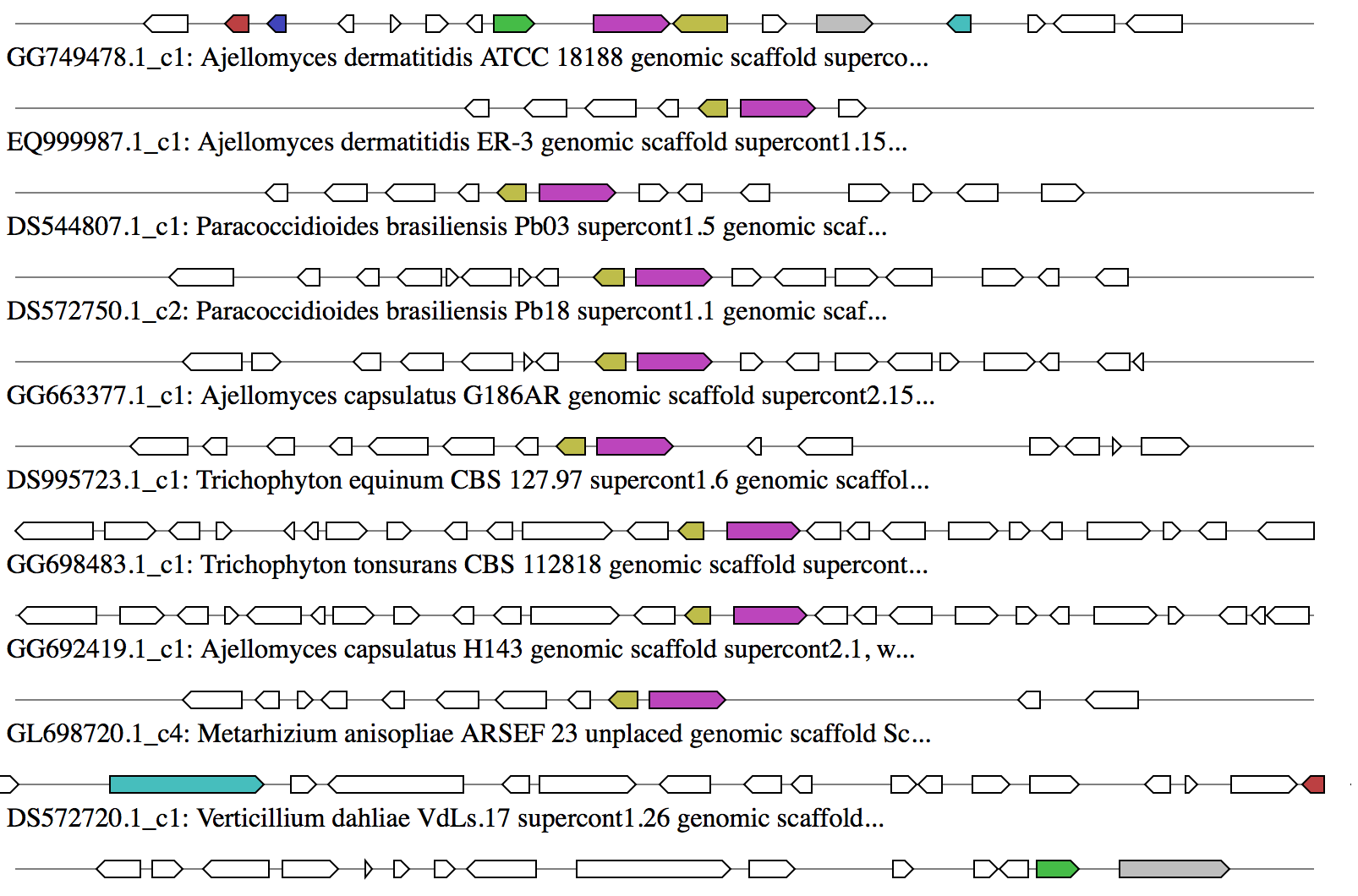
**

1. **mCaBGC15 - contig_131 - T1pks**

**
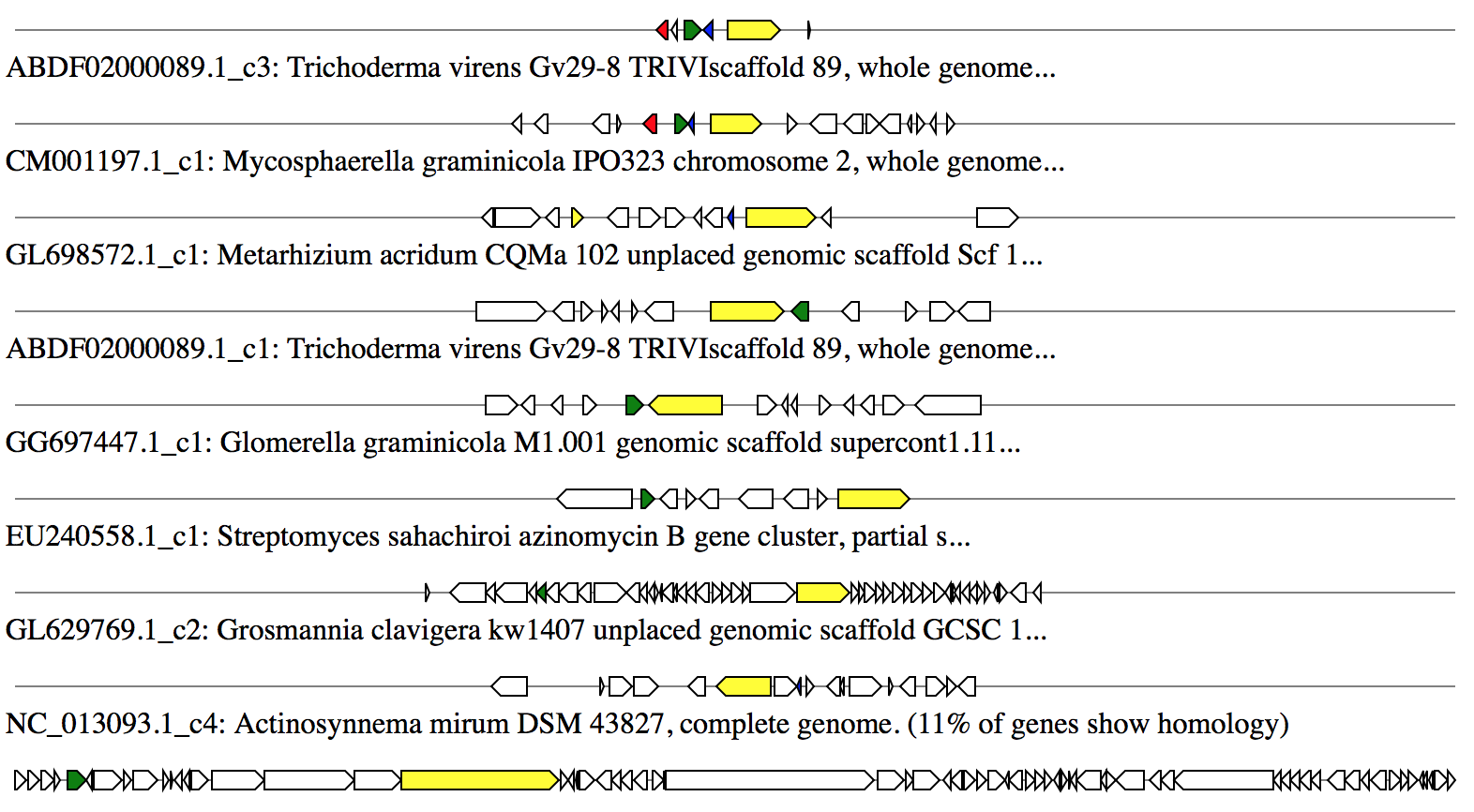
**

1. **mCaBGC16 - contig_158 – Nrps**

**
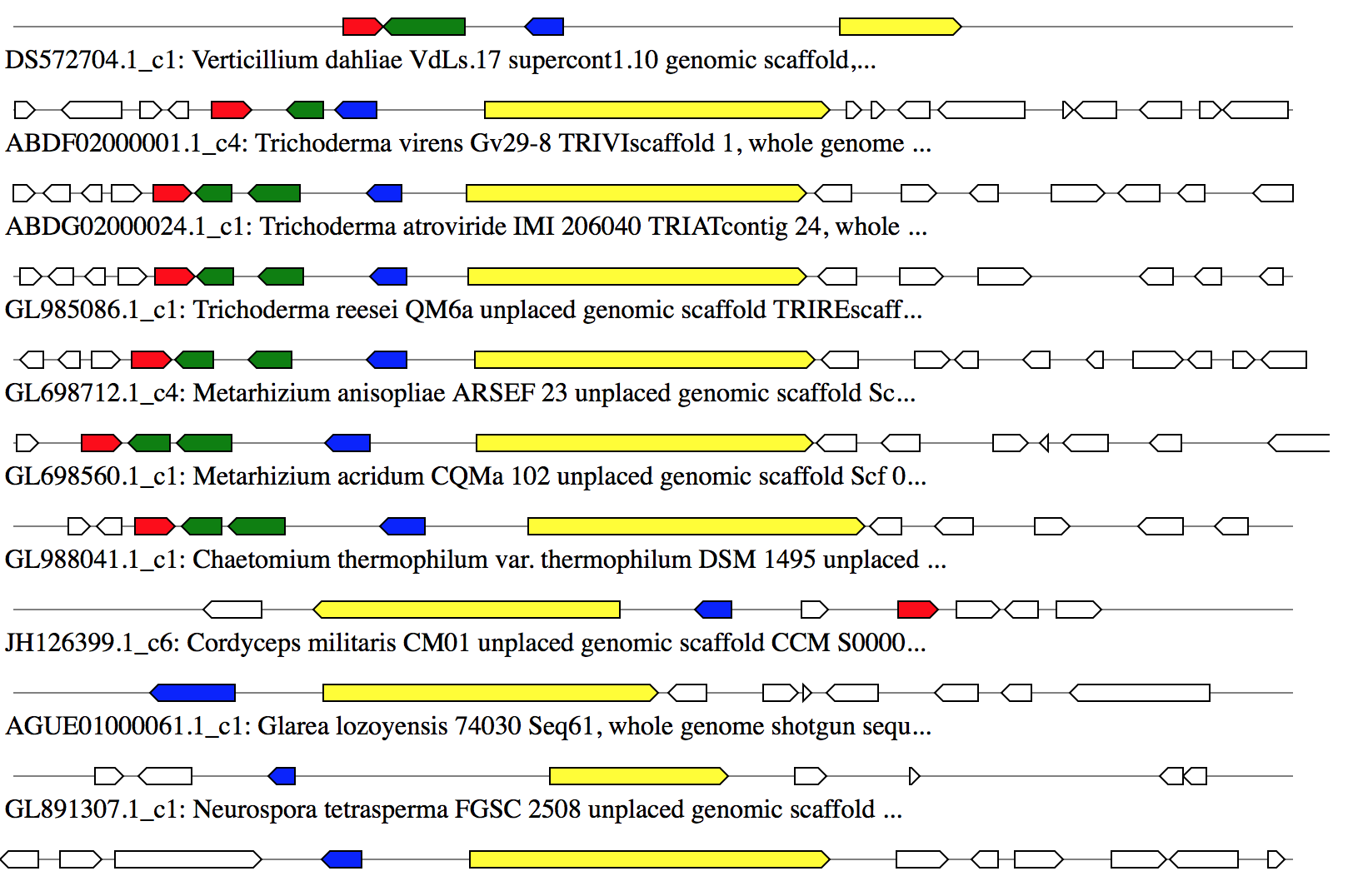
**

1. **mCaBGC17 - contig_159 – Terpene**

**
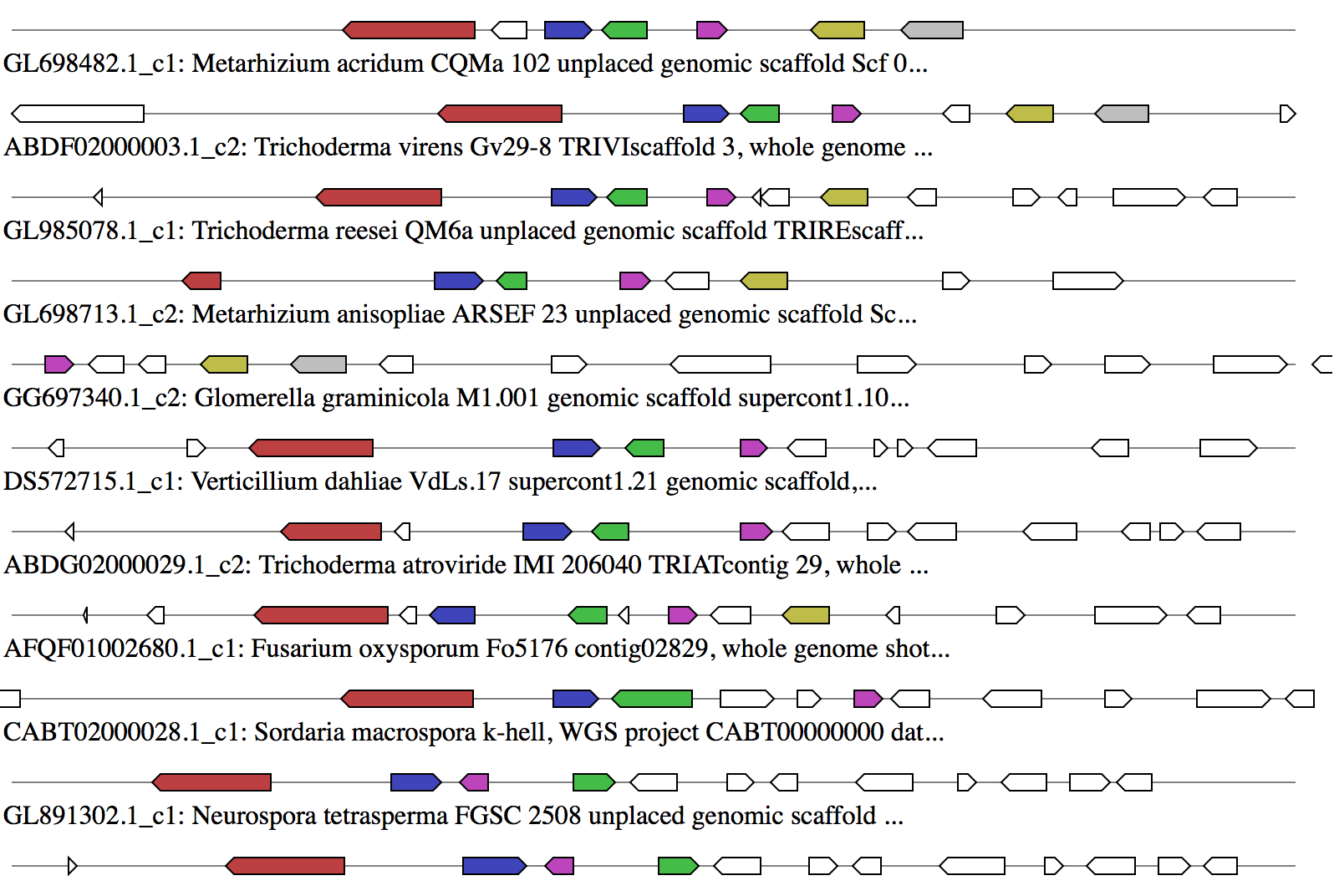
**

1. **mCaBGC18 - contig_161 – Nrps**

**
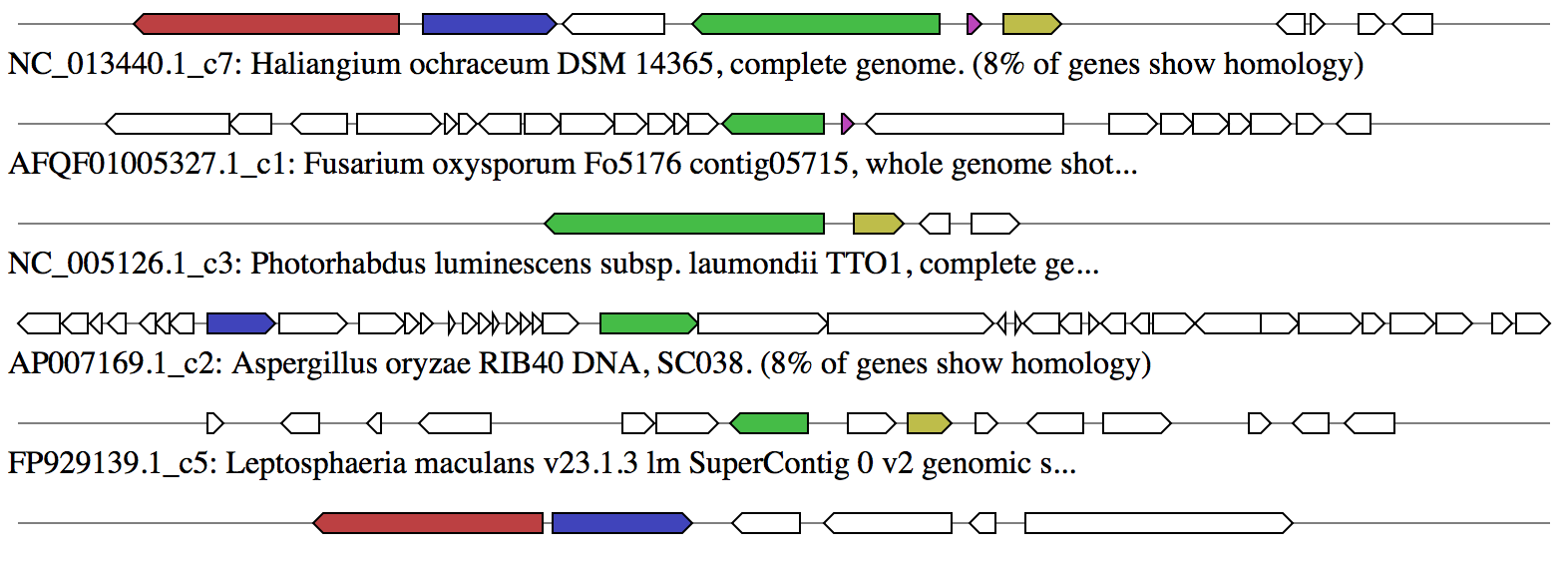
**

1. **mCaBGC19 - contig_165 – Terpene**

**
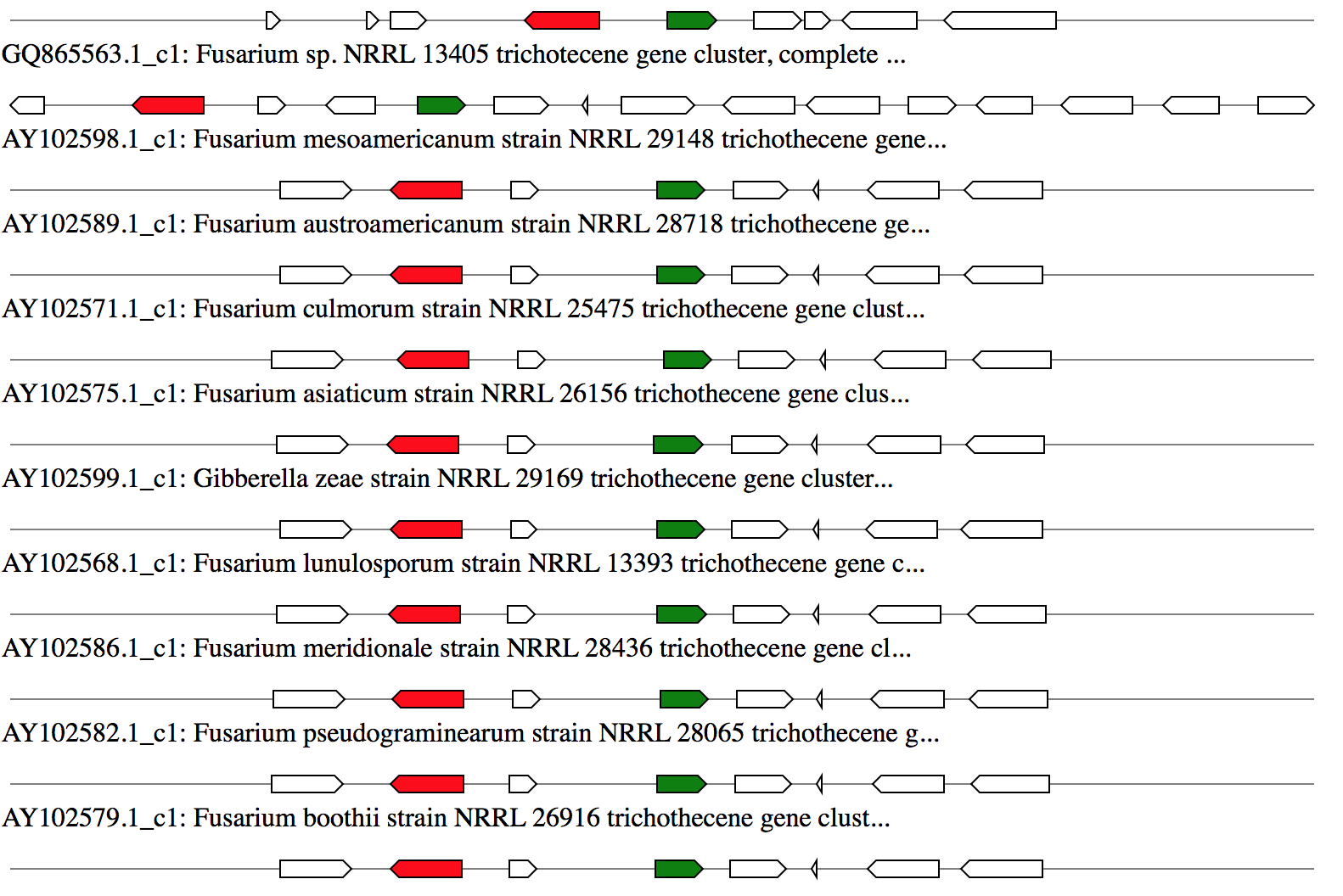
**

1. **mCaBGC20 - contig_169 - T1pks**

**
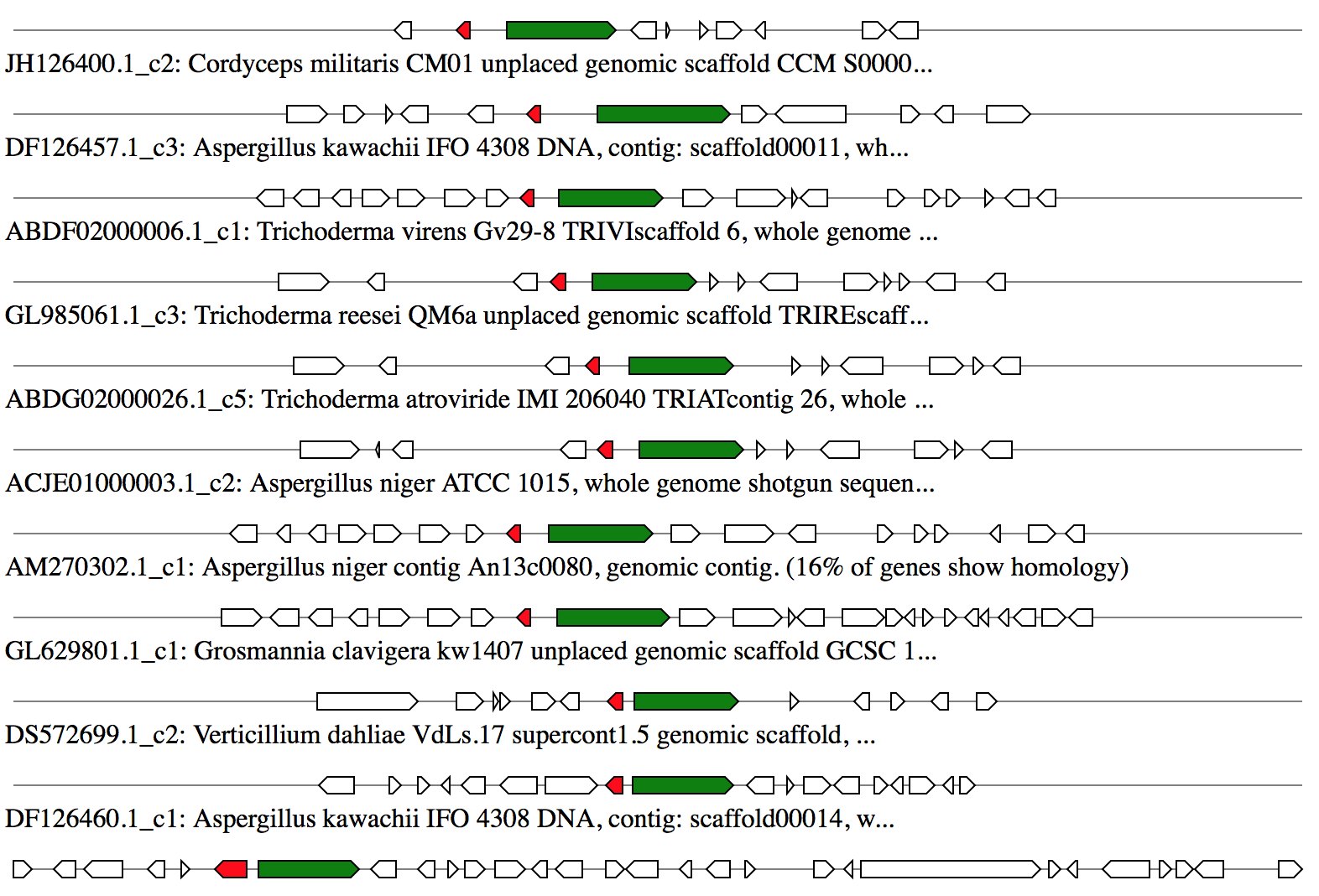
**

1. **mCaBGC21 - contig_169 - T1pks**

**
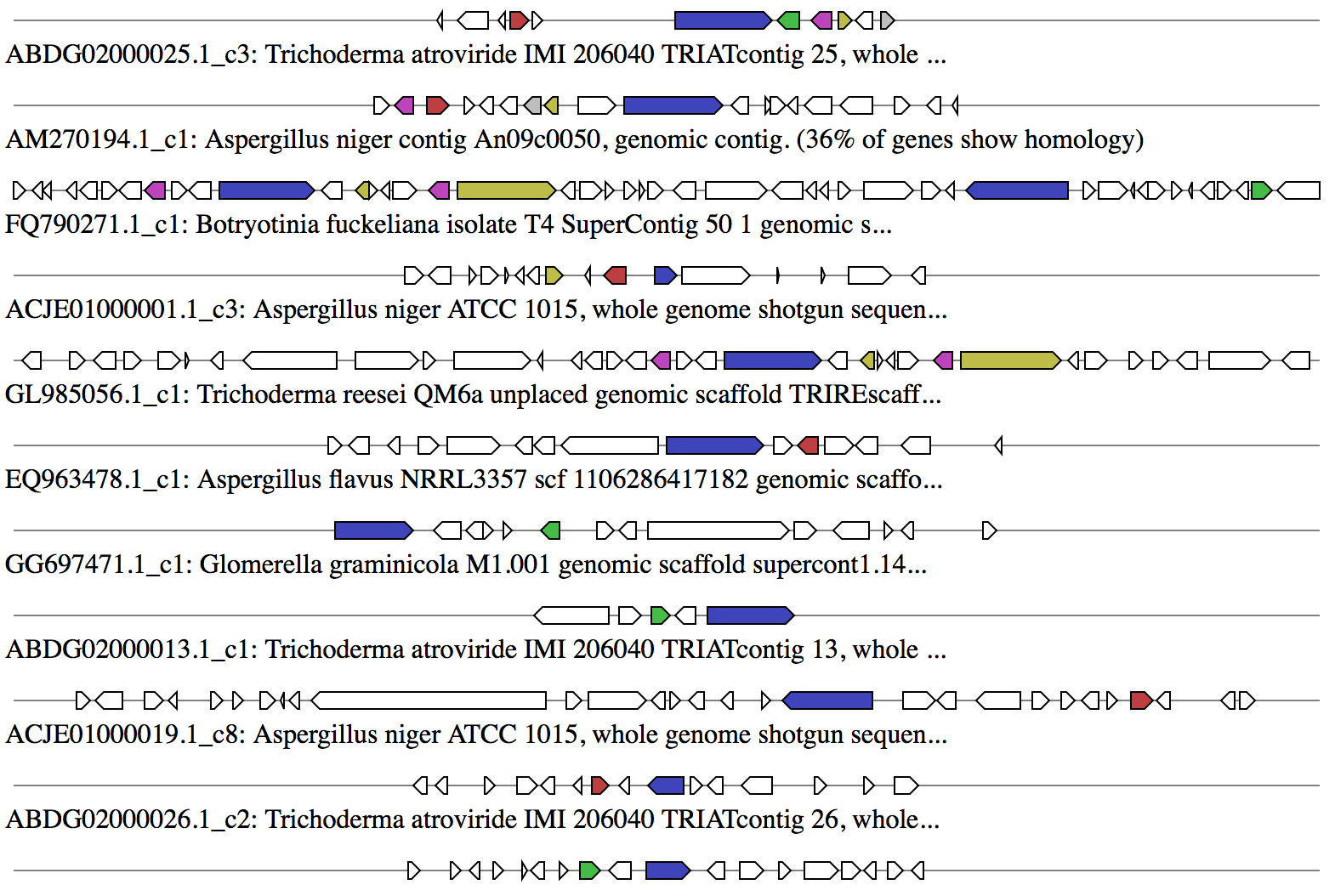
**

1. **mCaBGC22 - contig_173 – Other**

**
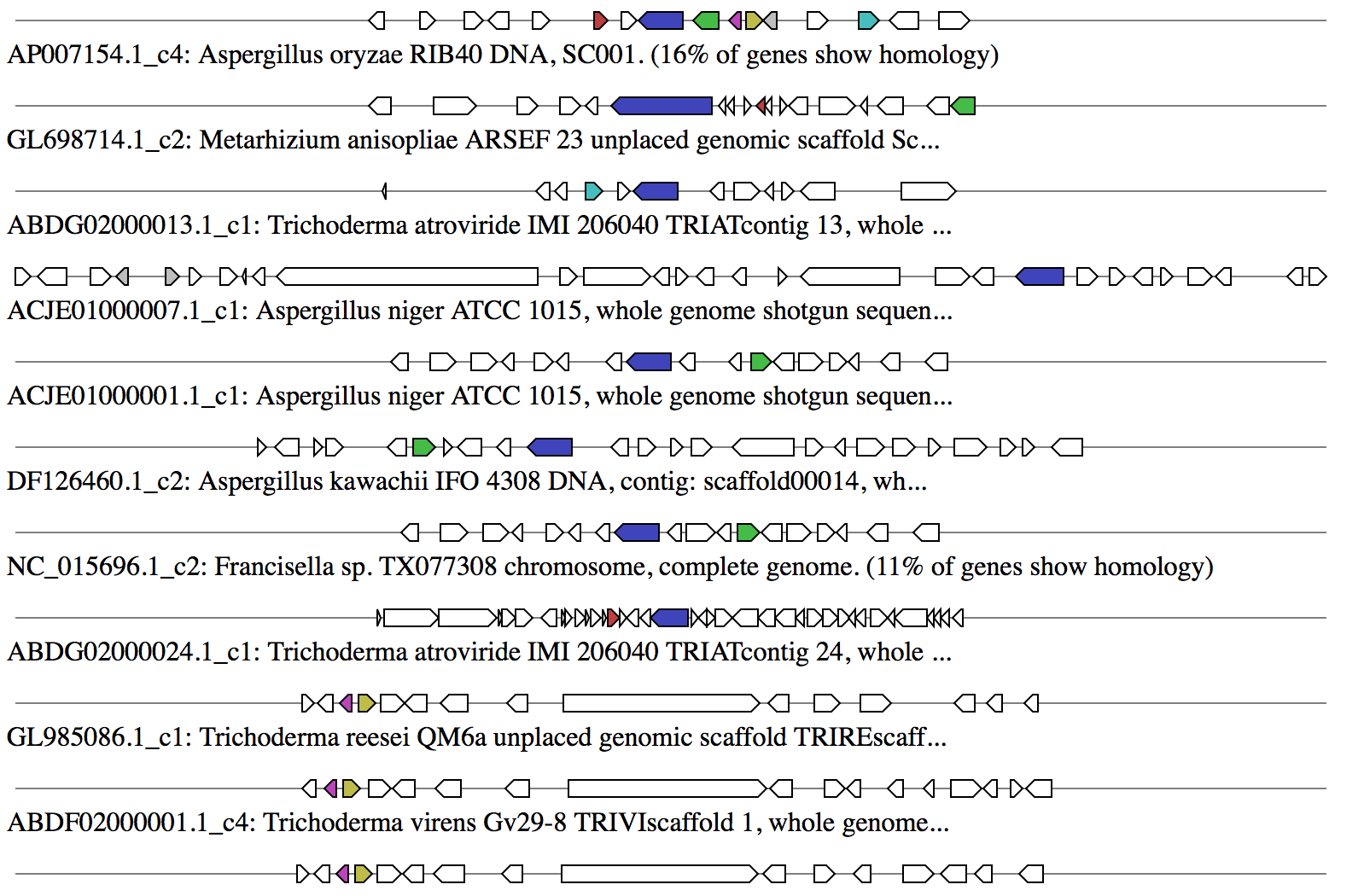
**

1. **mCaBGC23 - contig_175 – Nrps**

**
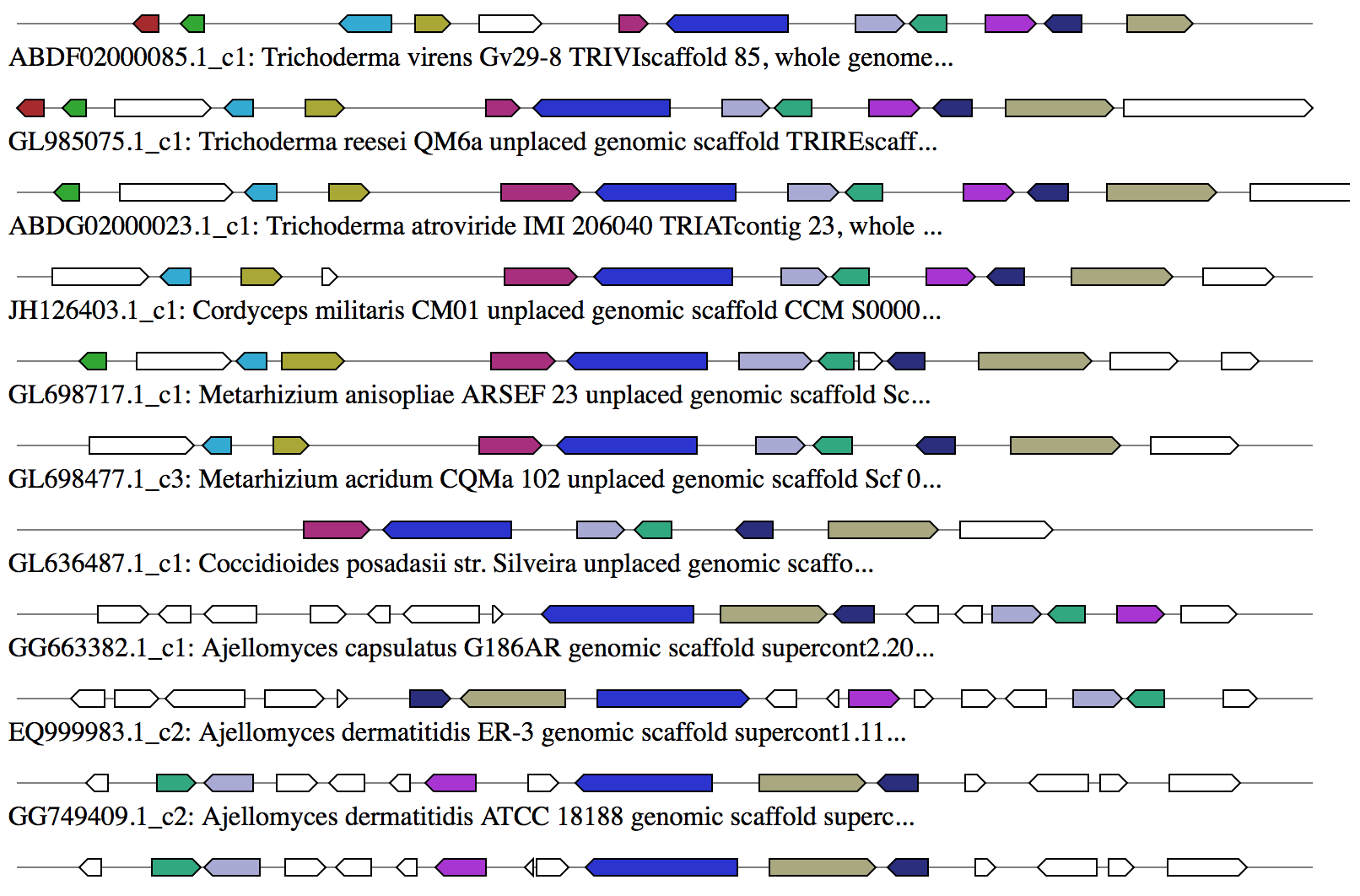
**

1. **mCaBGC24 - contig_178 - T1pks**

**
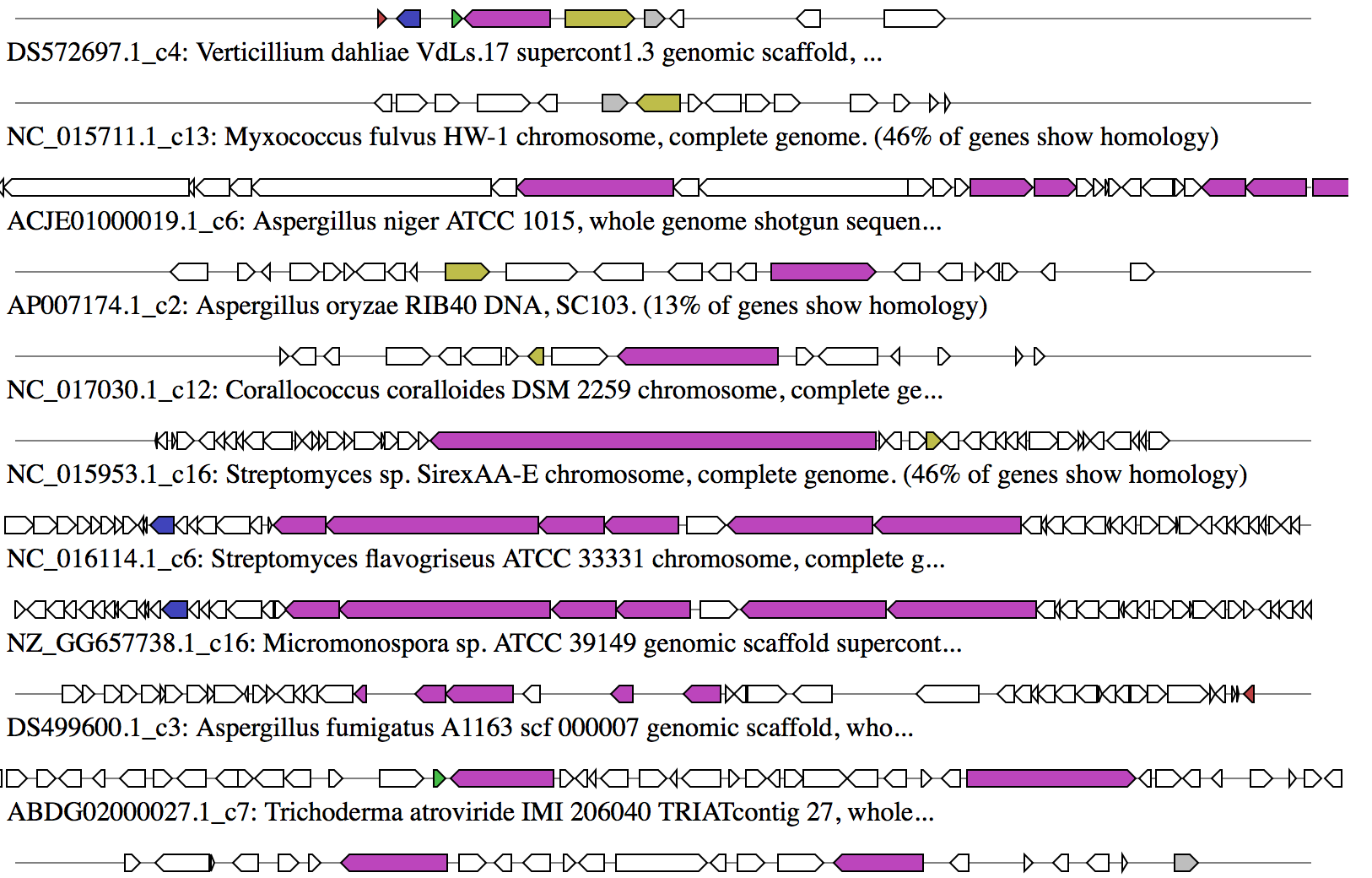
**

1. **mCaBGC25 - contig_189 - T1pks**

**
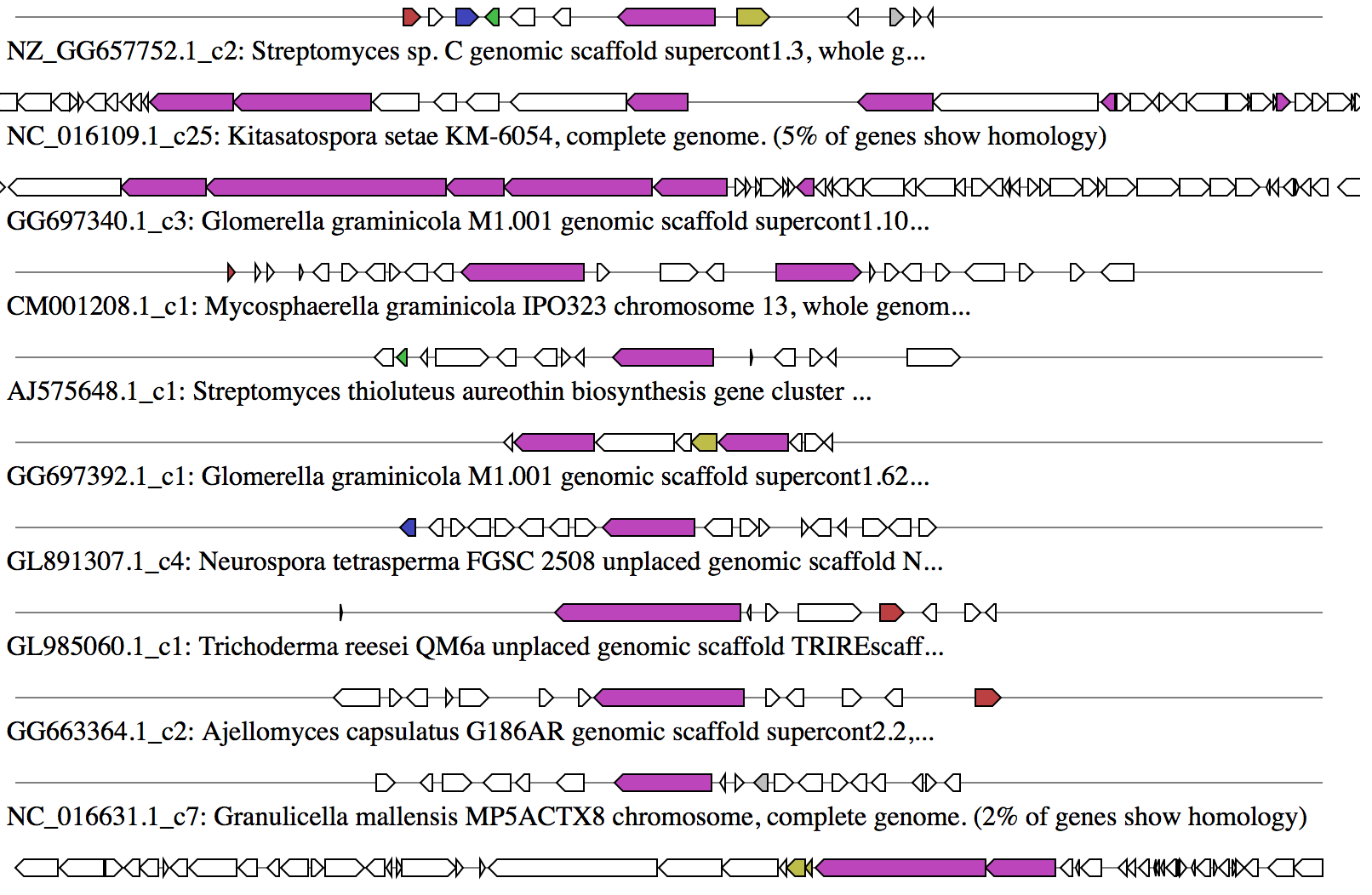
**

1. **mCaBGC26 - contig_201 - T1pks**

**
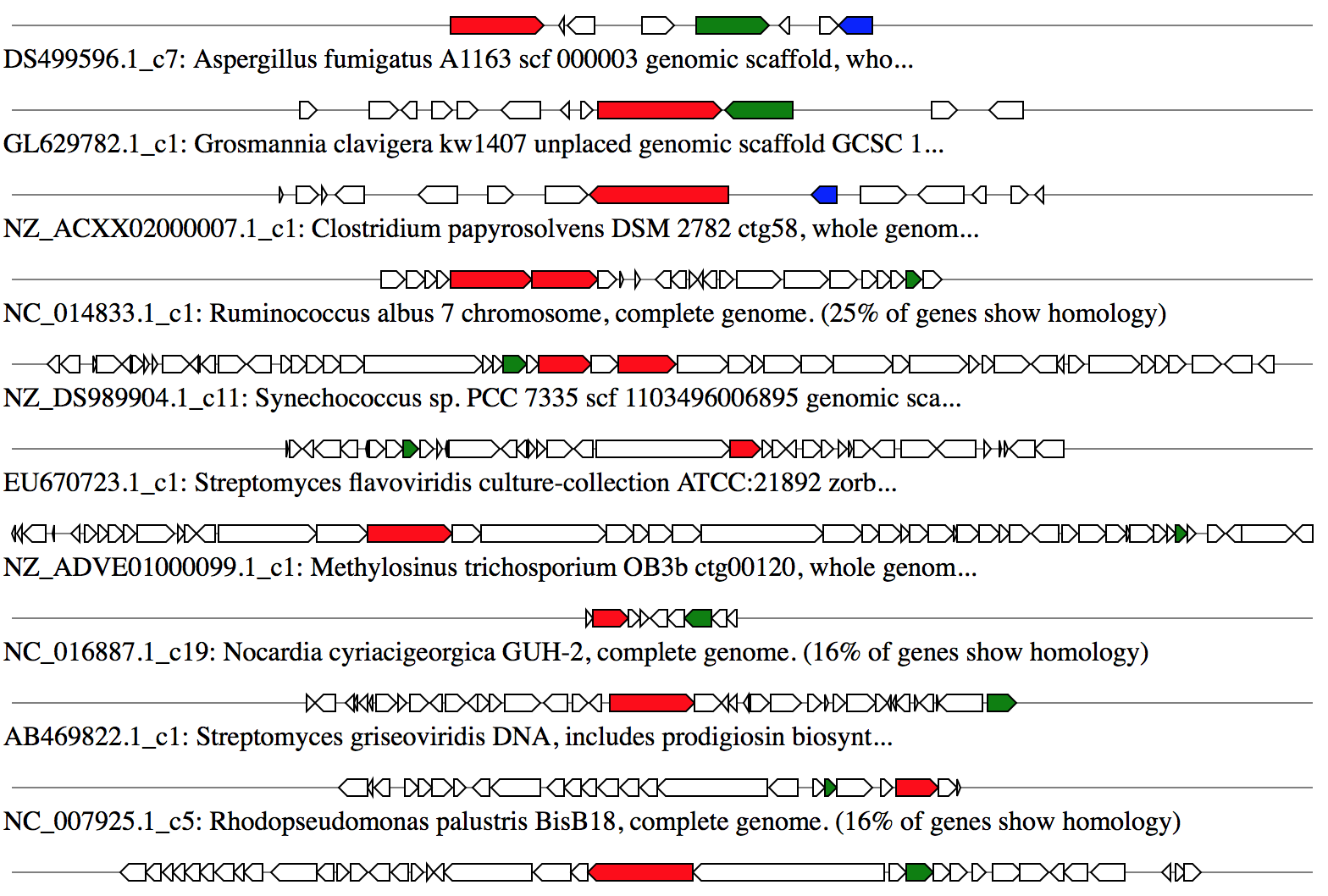
**

1. **mCaBGC27 - contig_219 – Nrps**

**
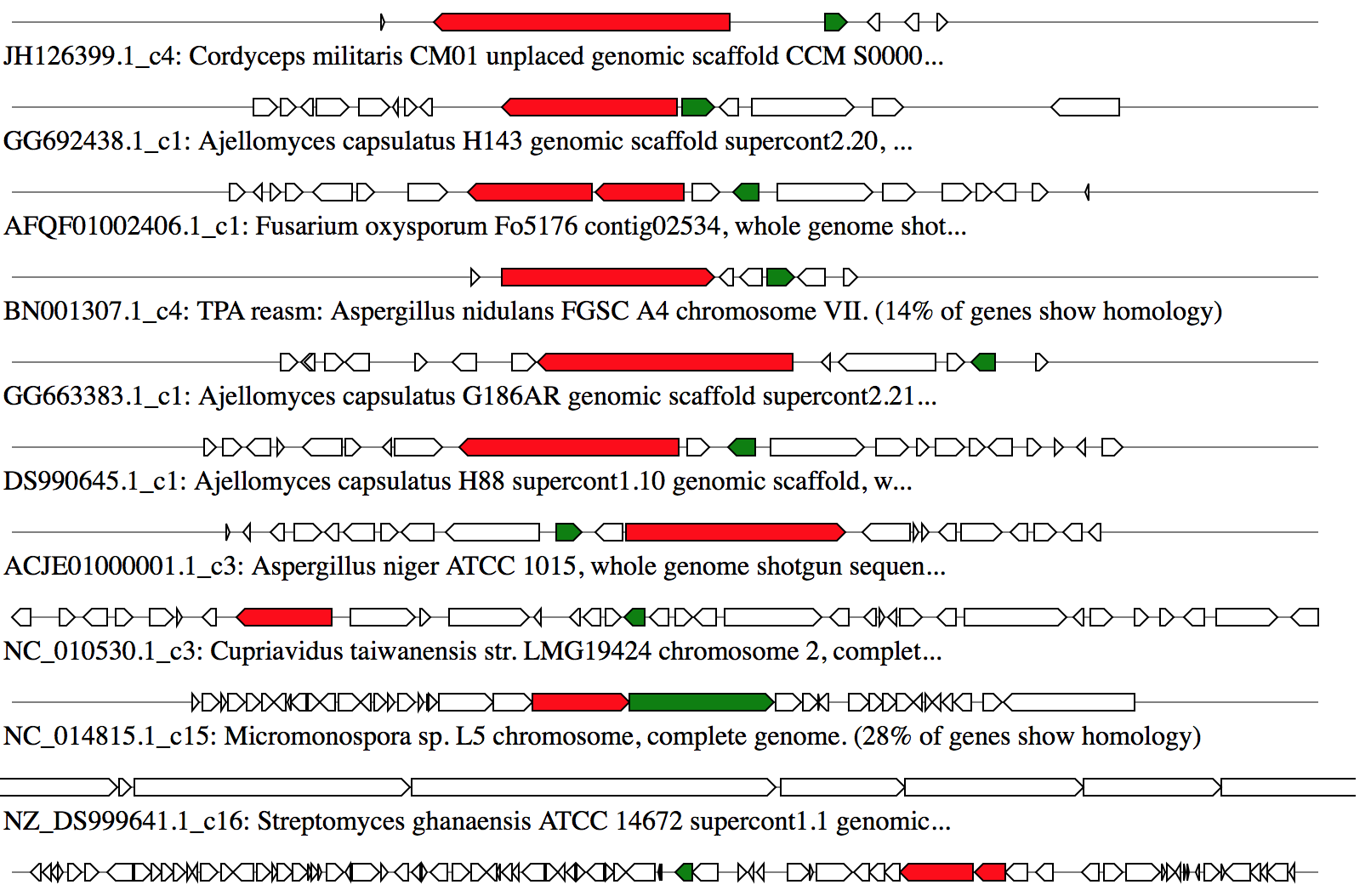
**

1. **mCaBGC28 - contig_221 – Nrps**

**
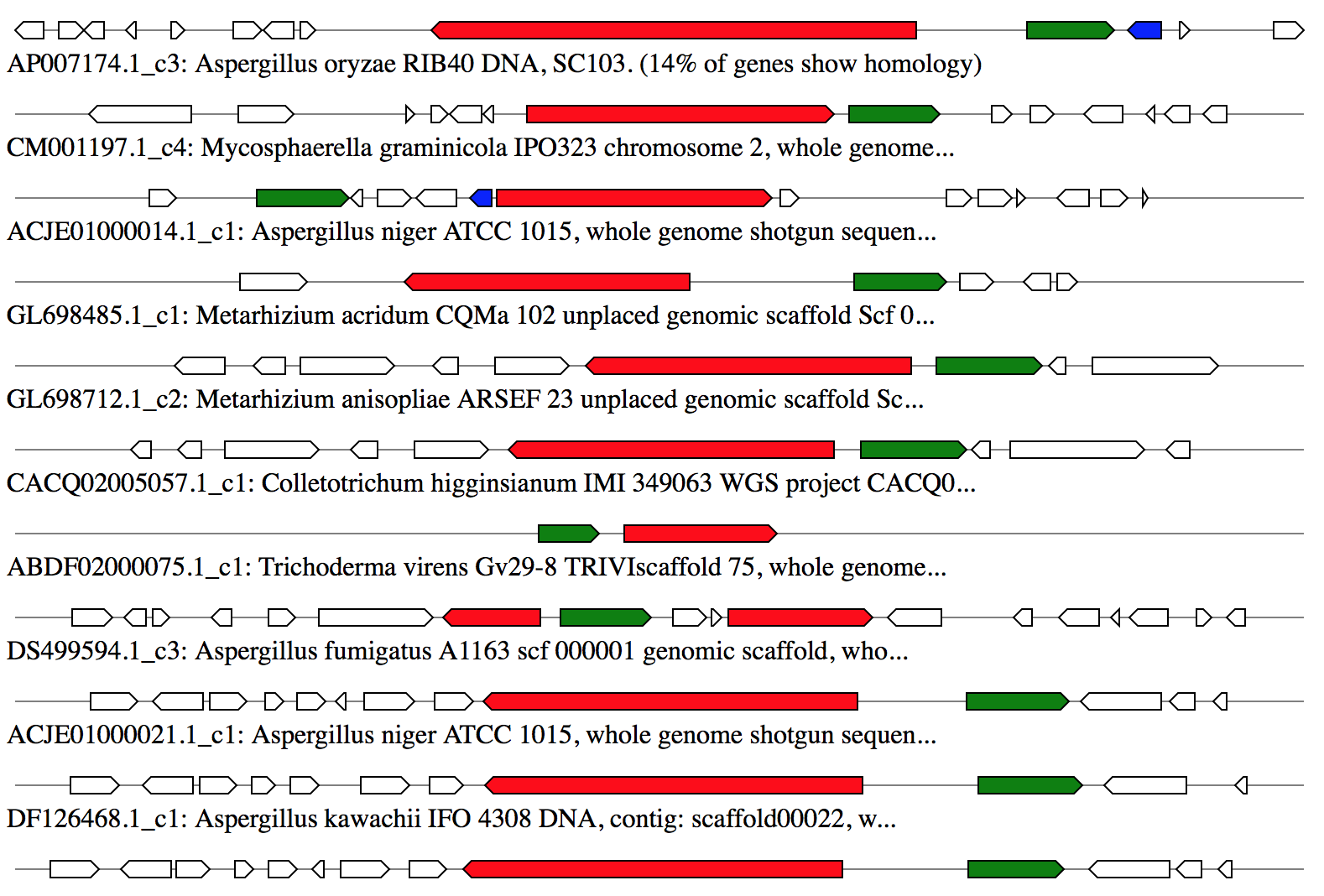
#**

1. **mCaBGC29 - contig_233 - T1pks**

**
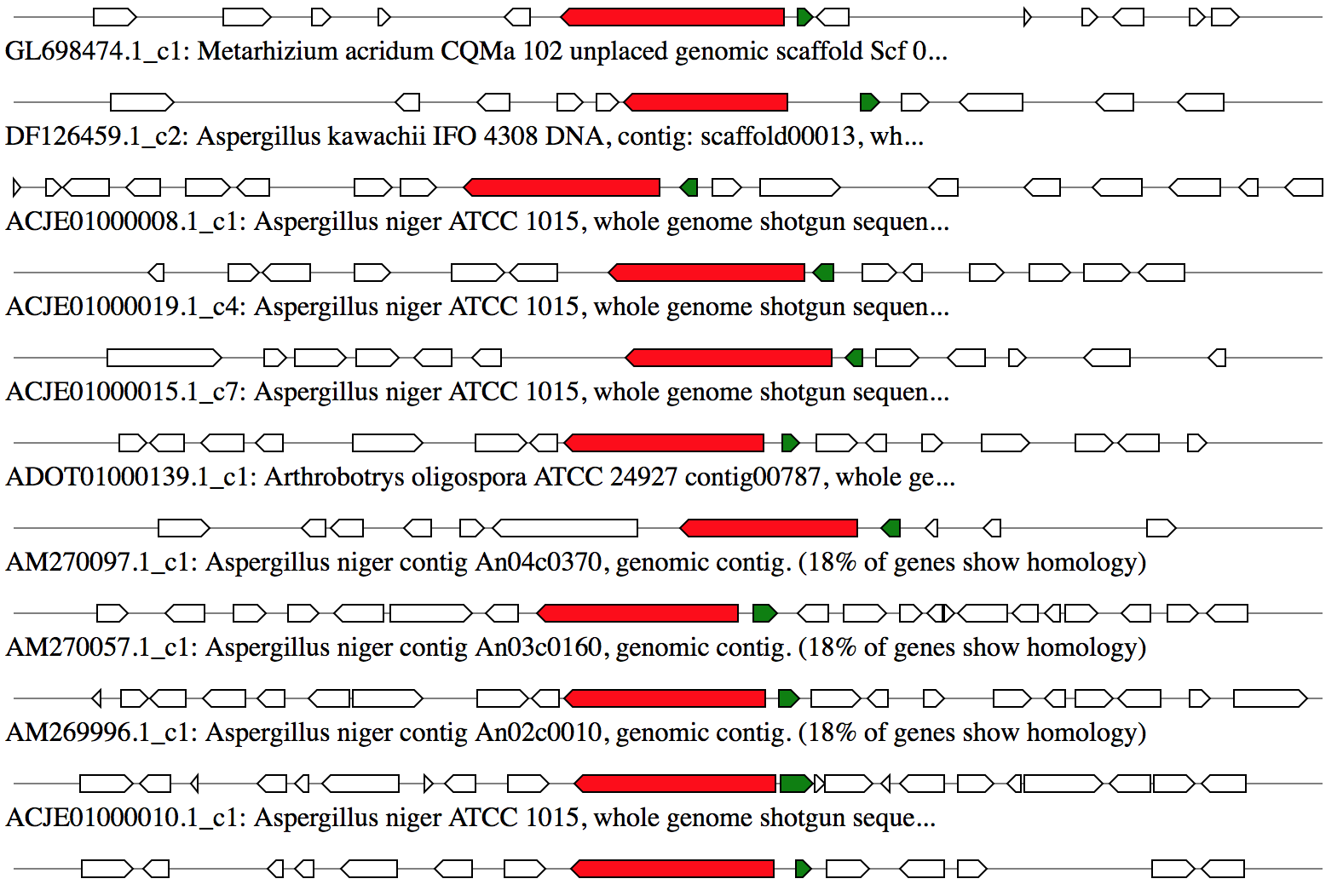
**

1. **mCaBGC30 - contig_245 – Terpene**

**
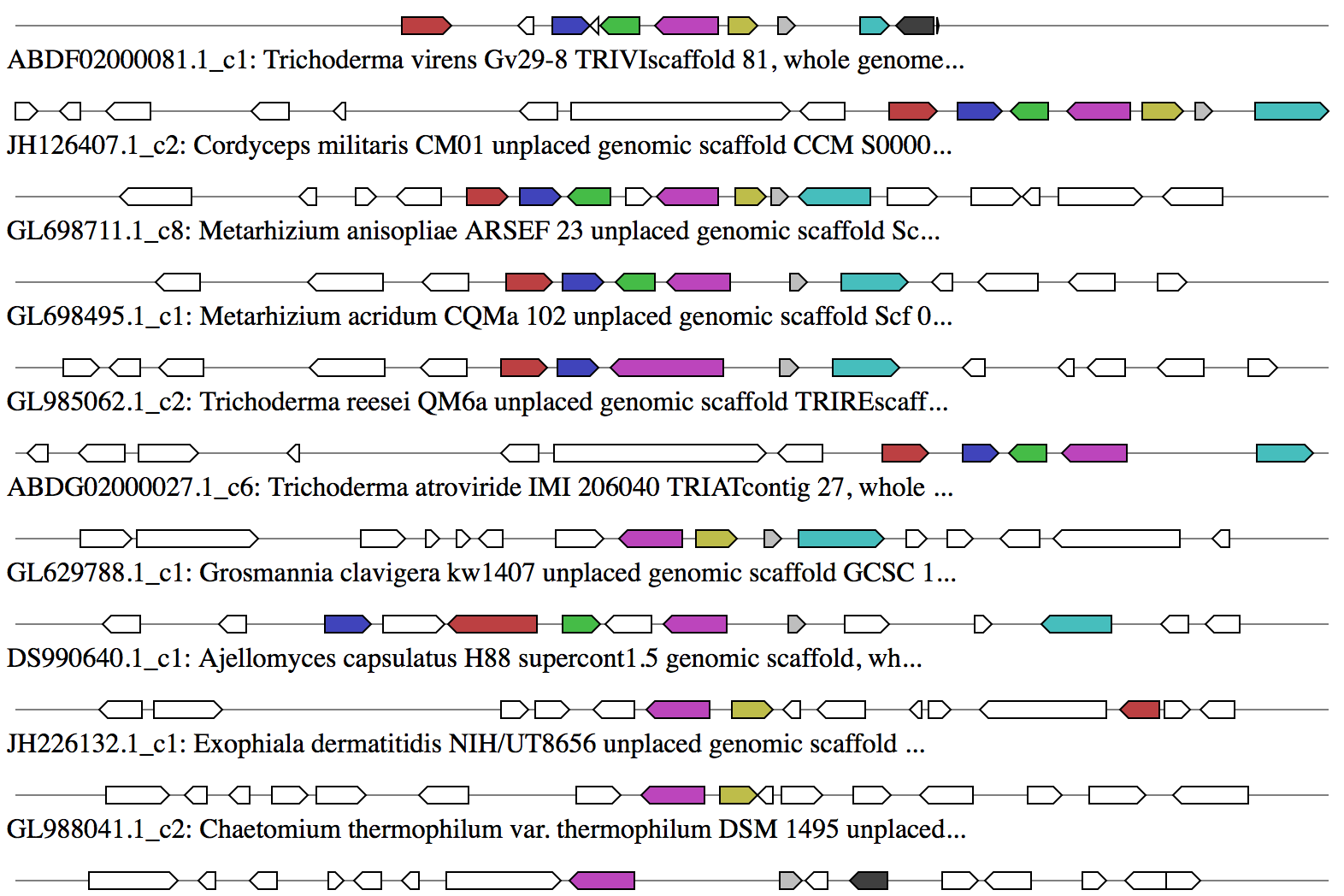
**

1. **mCaBGC31 - contig_270 – Other**

**
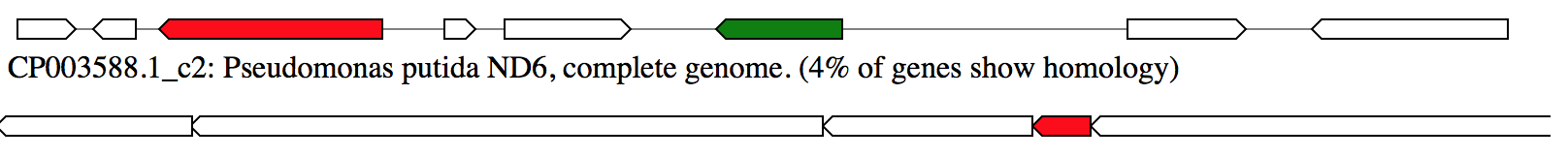
**

1. **mCaBGC32 - contig_272 - T1pks-nrps**

**
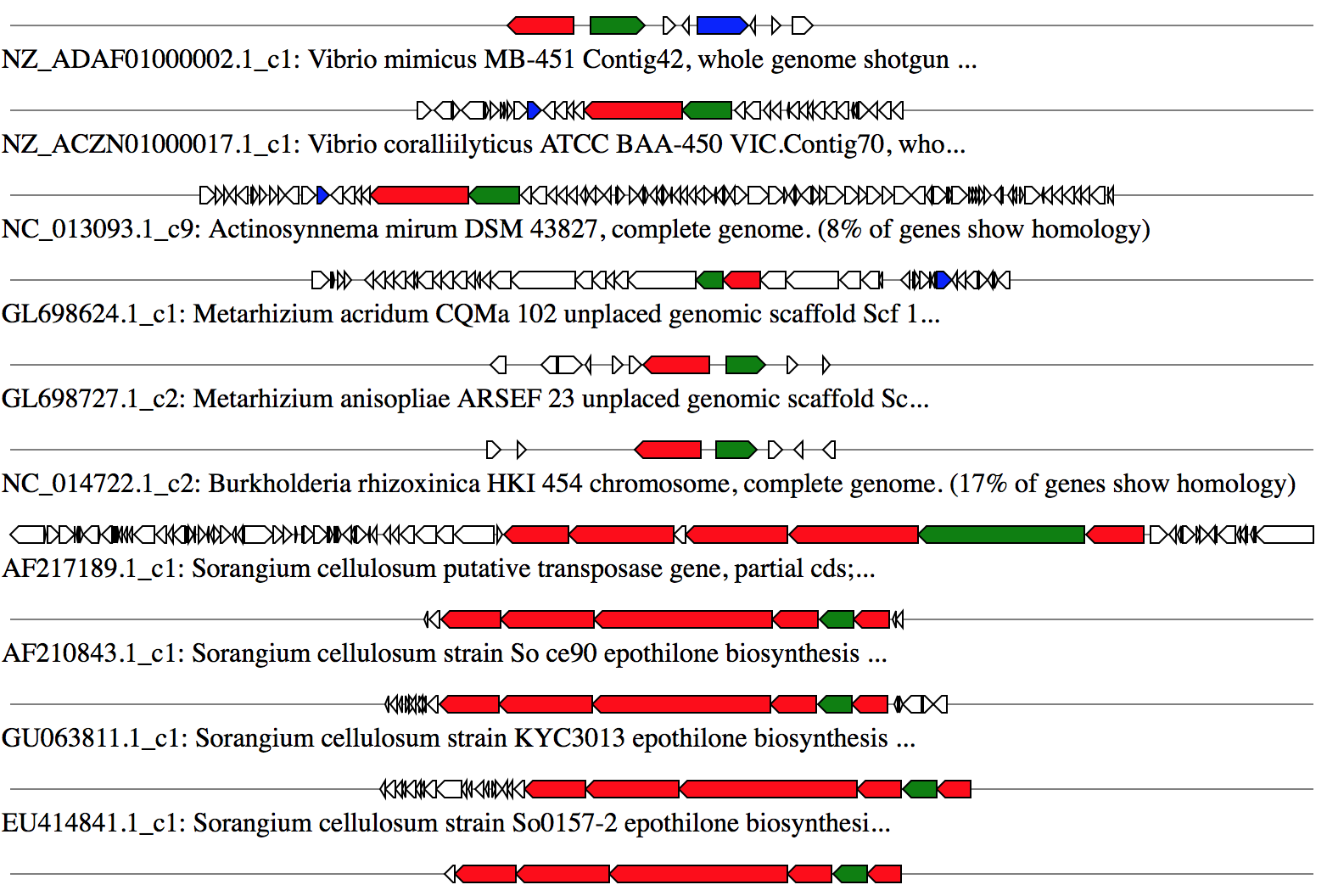
**

1. **mCaBGC33 - contig_274 – Hglks**

**
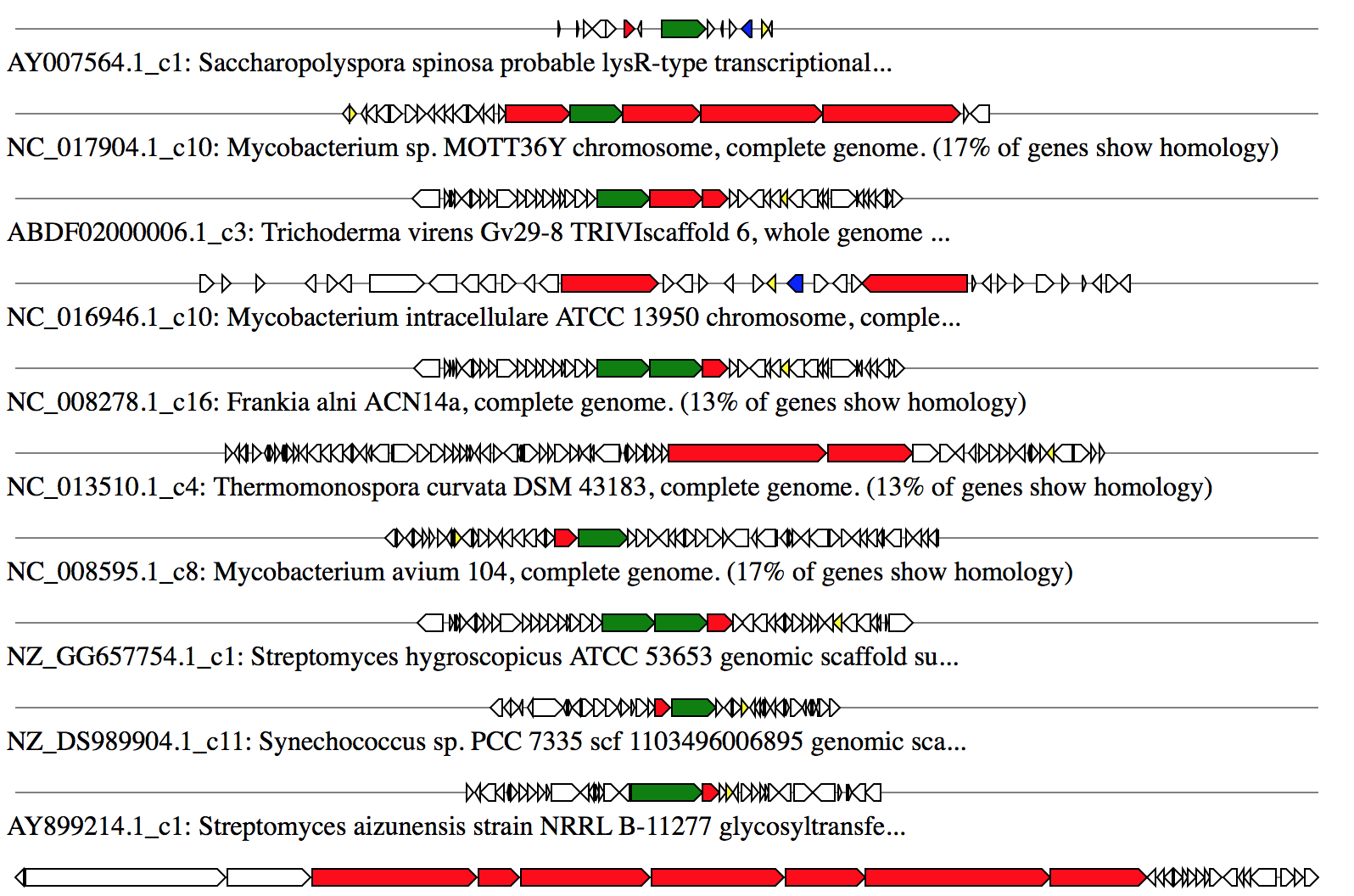
**

1. **mCaBGC34 - contig_280 - T1pks**

**
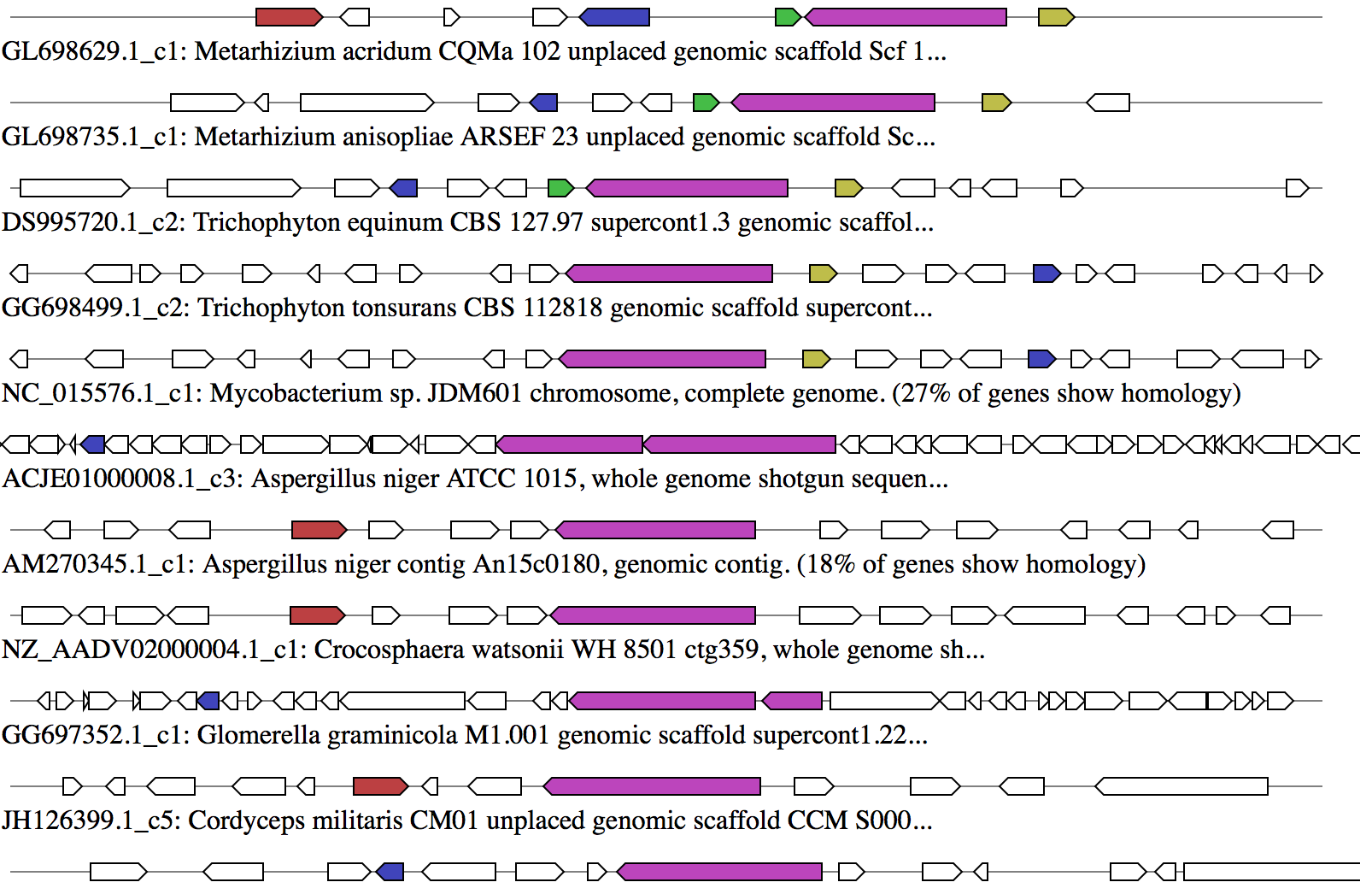
**

1. **mCaBGC35 - contig_282 - T1pks**

**
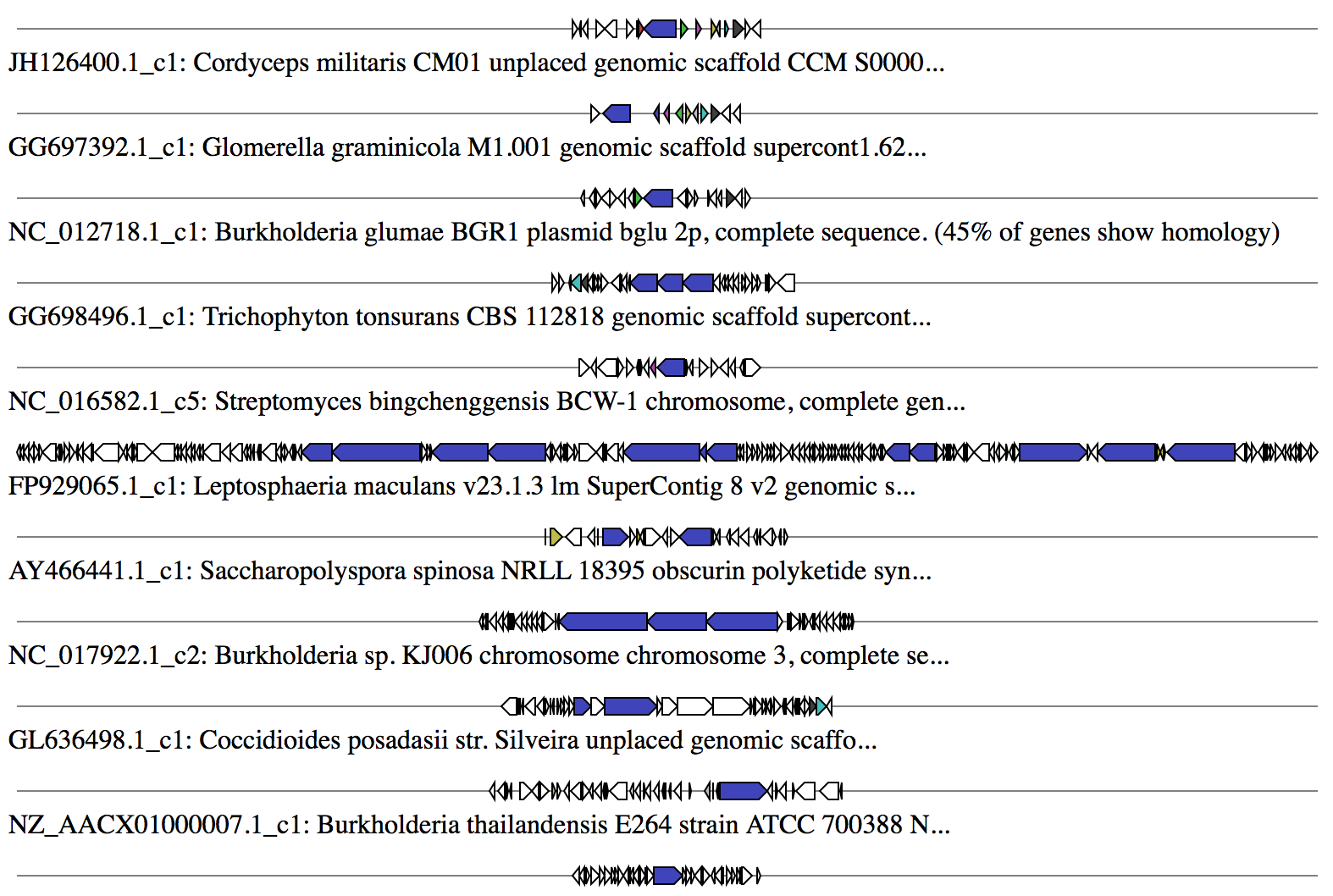
**

1. **mCaBGC36 - contig_284 - T1pks**

No homology clusters

1. **mCaBGC37 - contig_286 – Nrps**

**
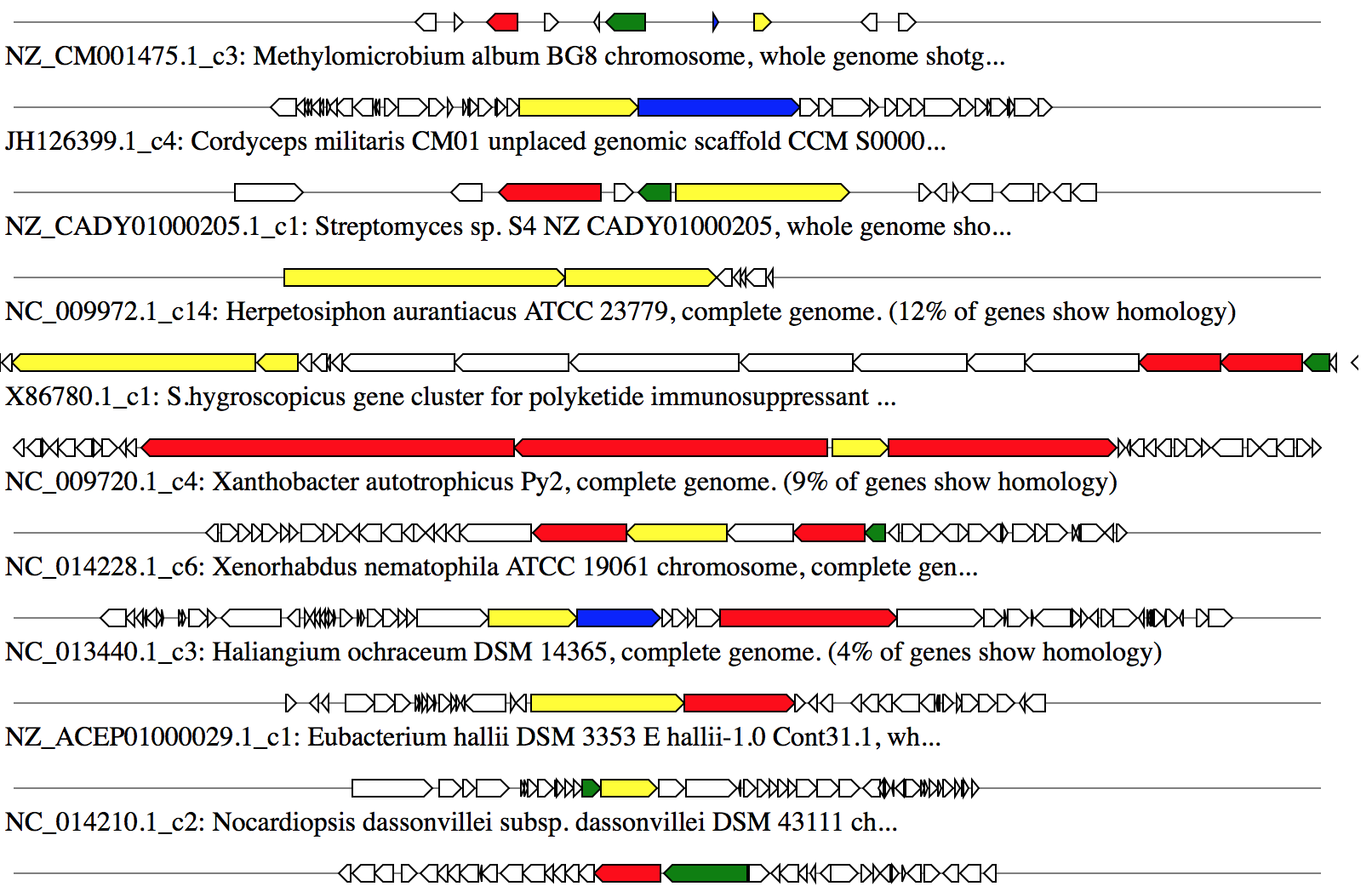
**

1. **mCaBGC38 - contig_325 – Other**

**
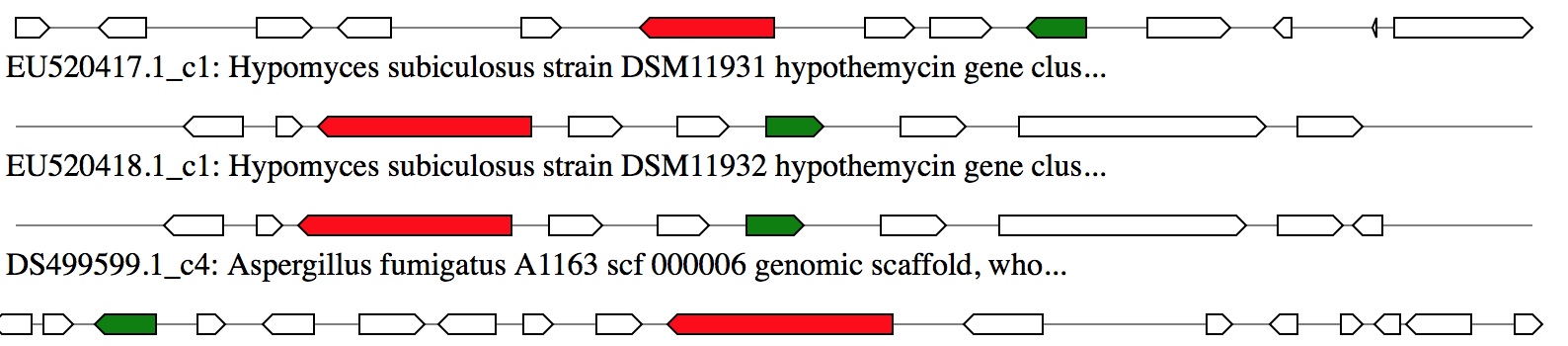
**

1. **mCaBGC39 - contig_333 – Other**

**
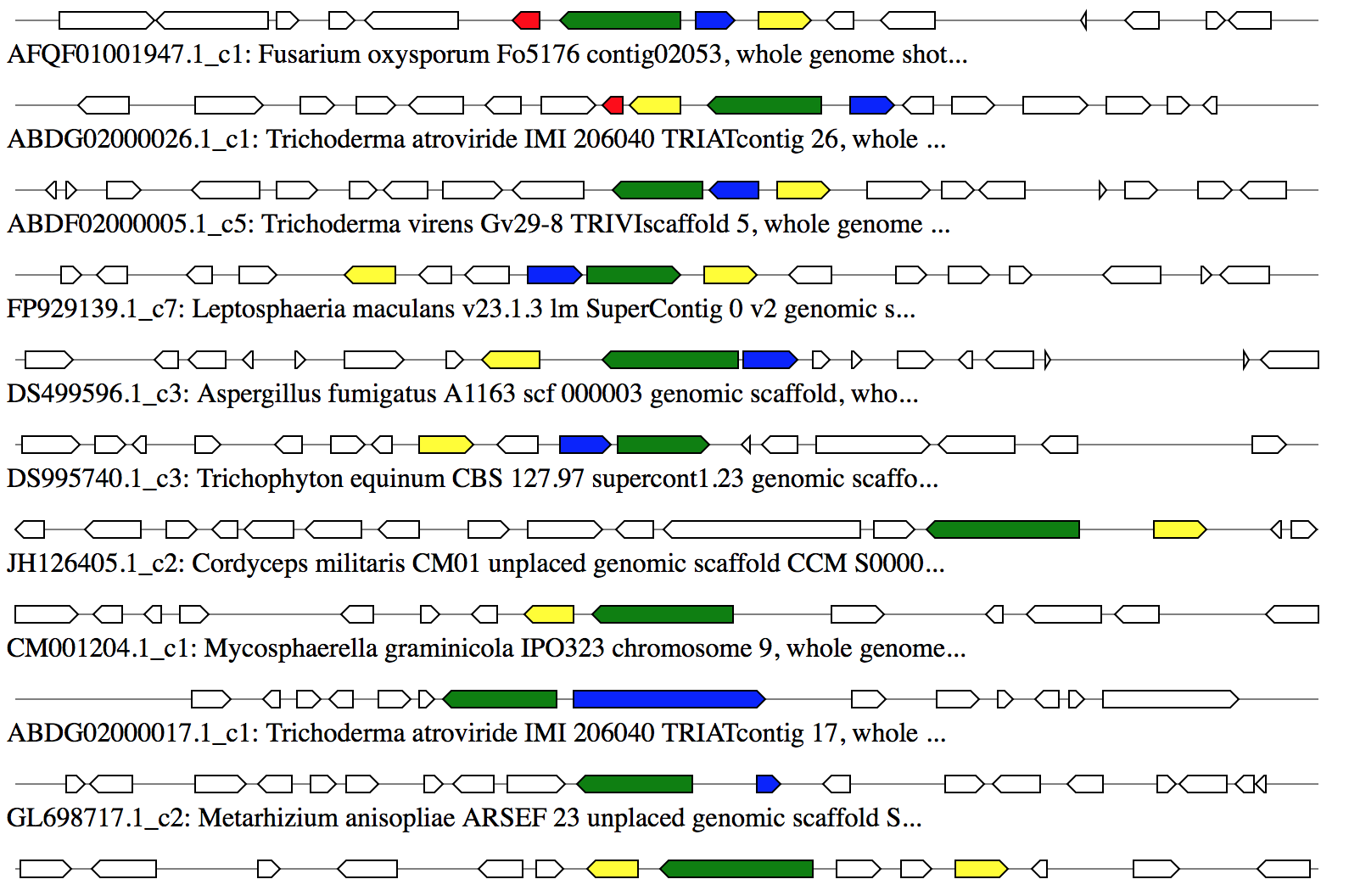
**

1. **mCaBGC40 - contig_358 - T1pks**

**
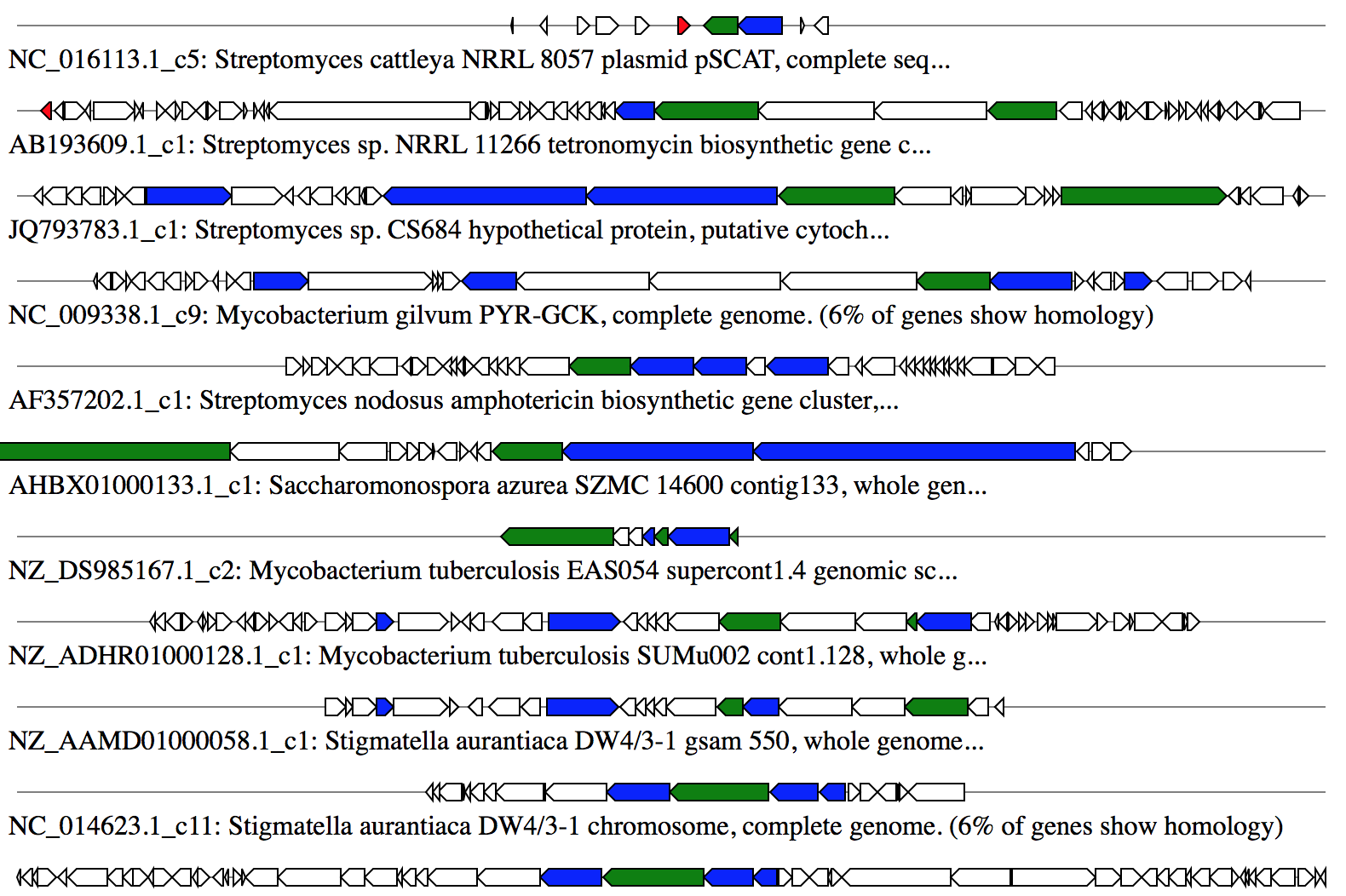
**

1. **mCaBGC41 - contig_367 - T1pks**

**
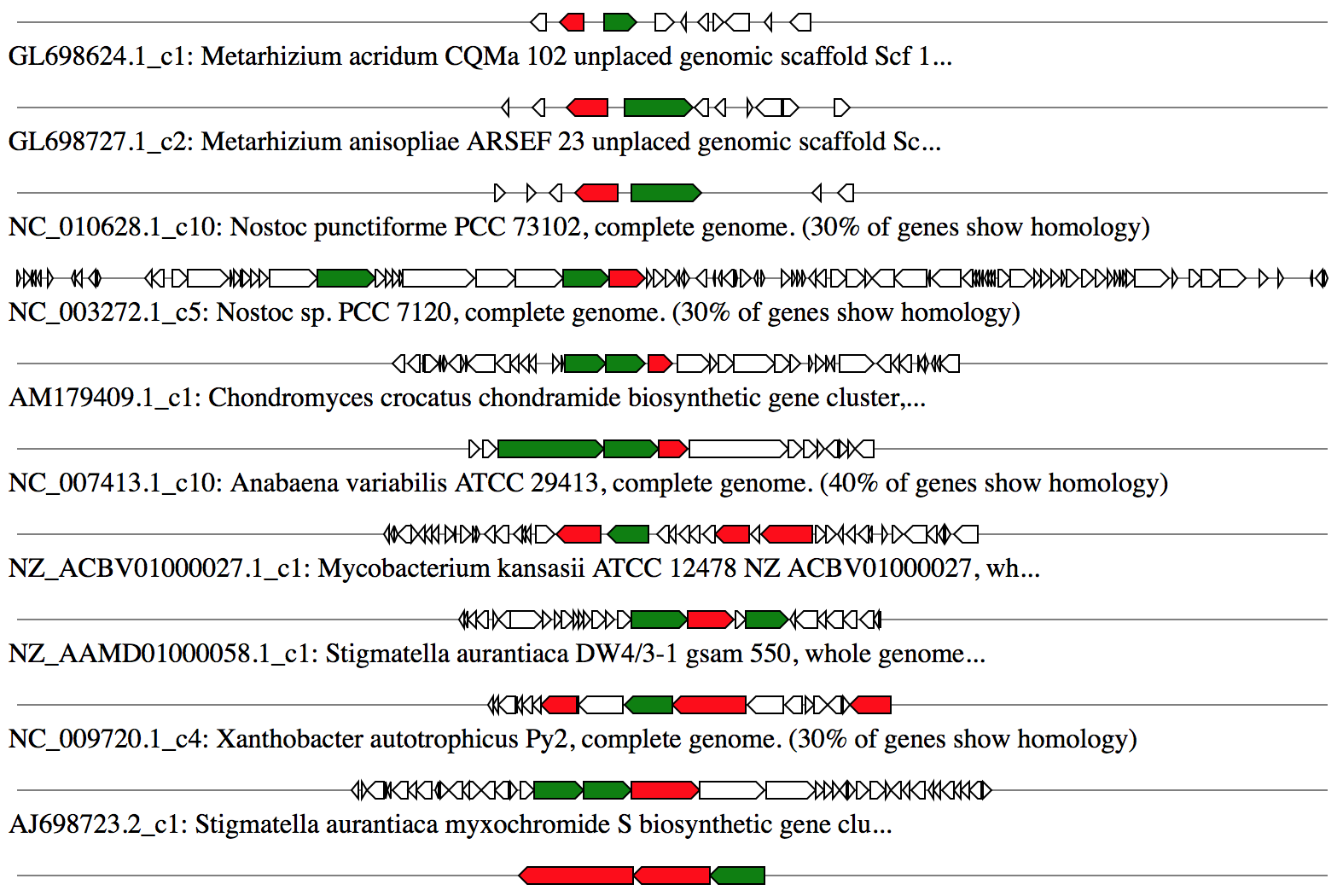
**

1. **mCaBGC42 - contig_371 - Nrps-t1pks**

**
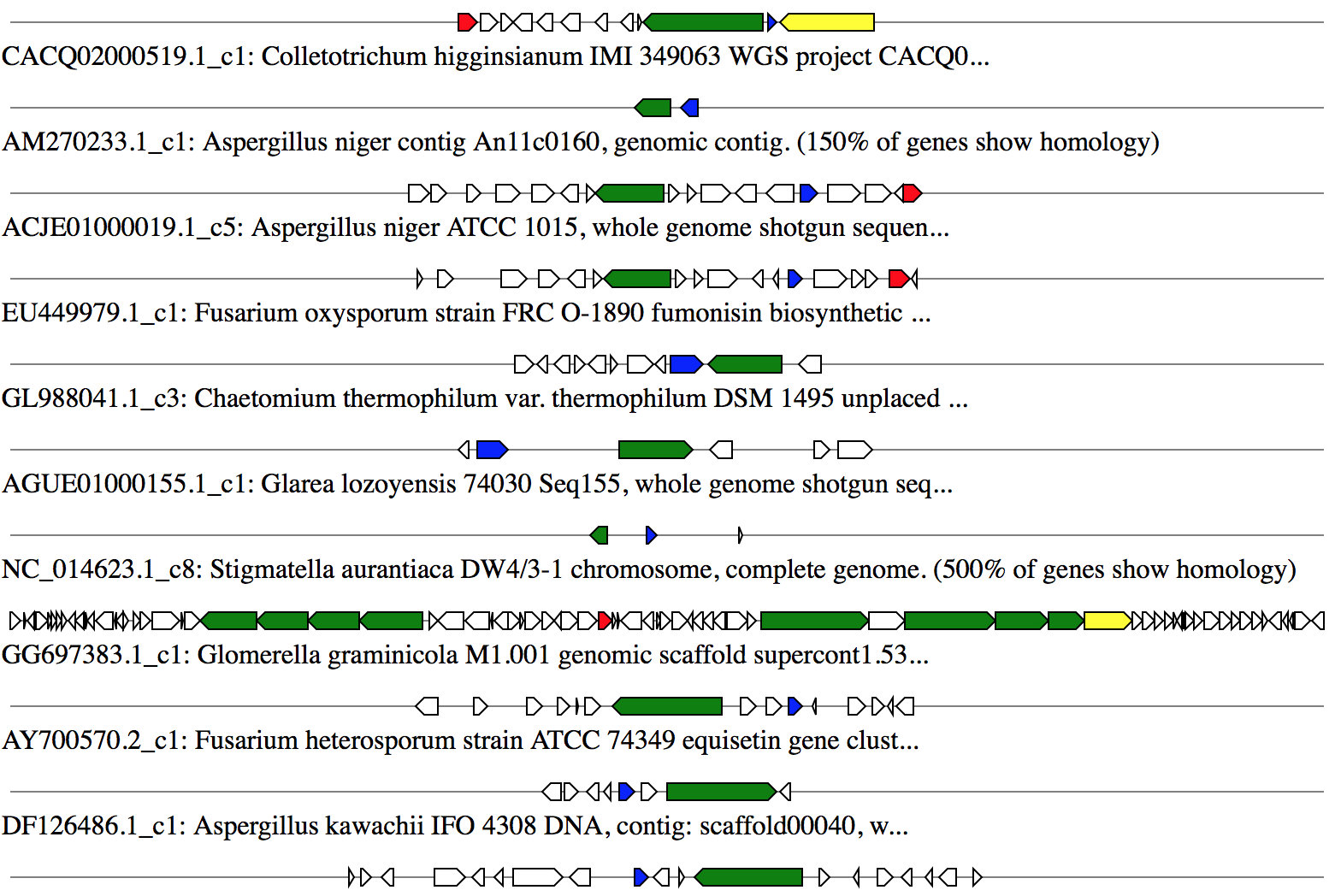
**

1. **mCaBGC43 - contig_372 – Nrps**

**
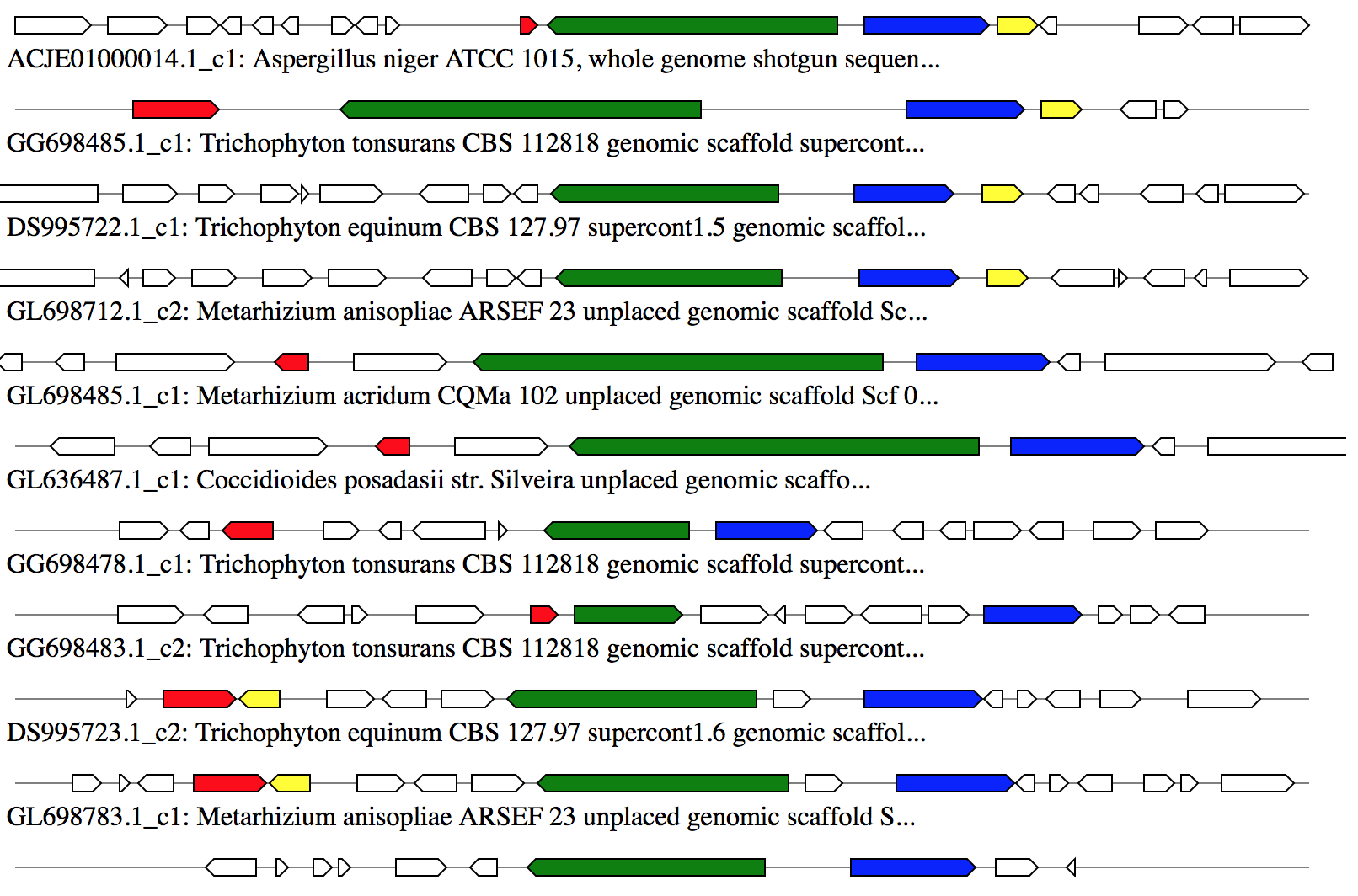
**

1. **mCaBGC44 - contig_400 – Nrps**

No Significant hit

1. **mCaBGC45 - contig_413 - T1pks**

**
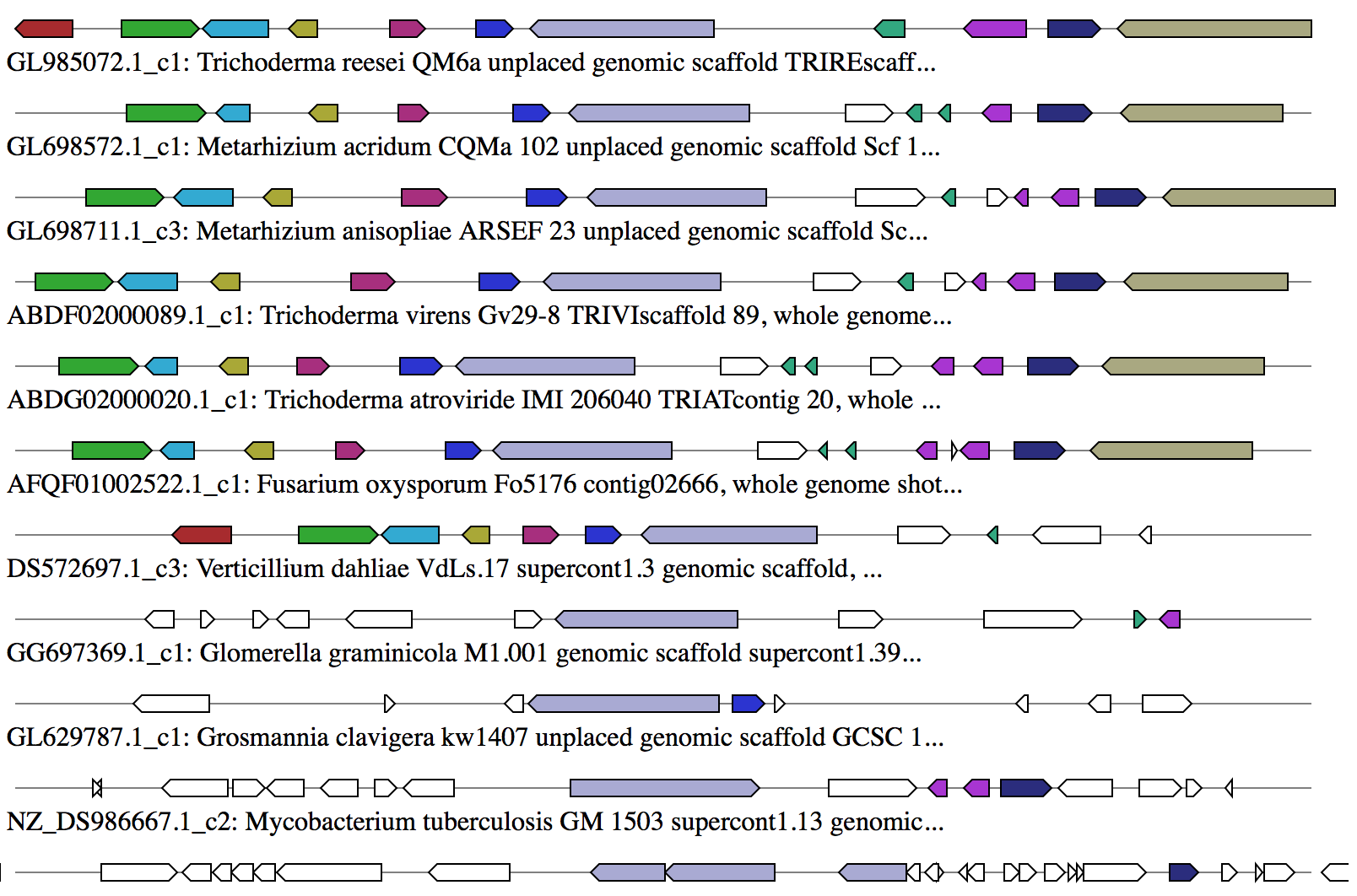
**

1. **mCaBGC46 - contig_422 – Hglks**

**
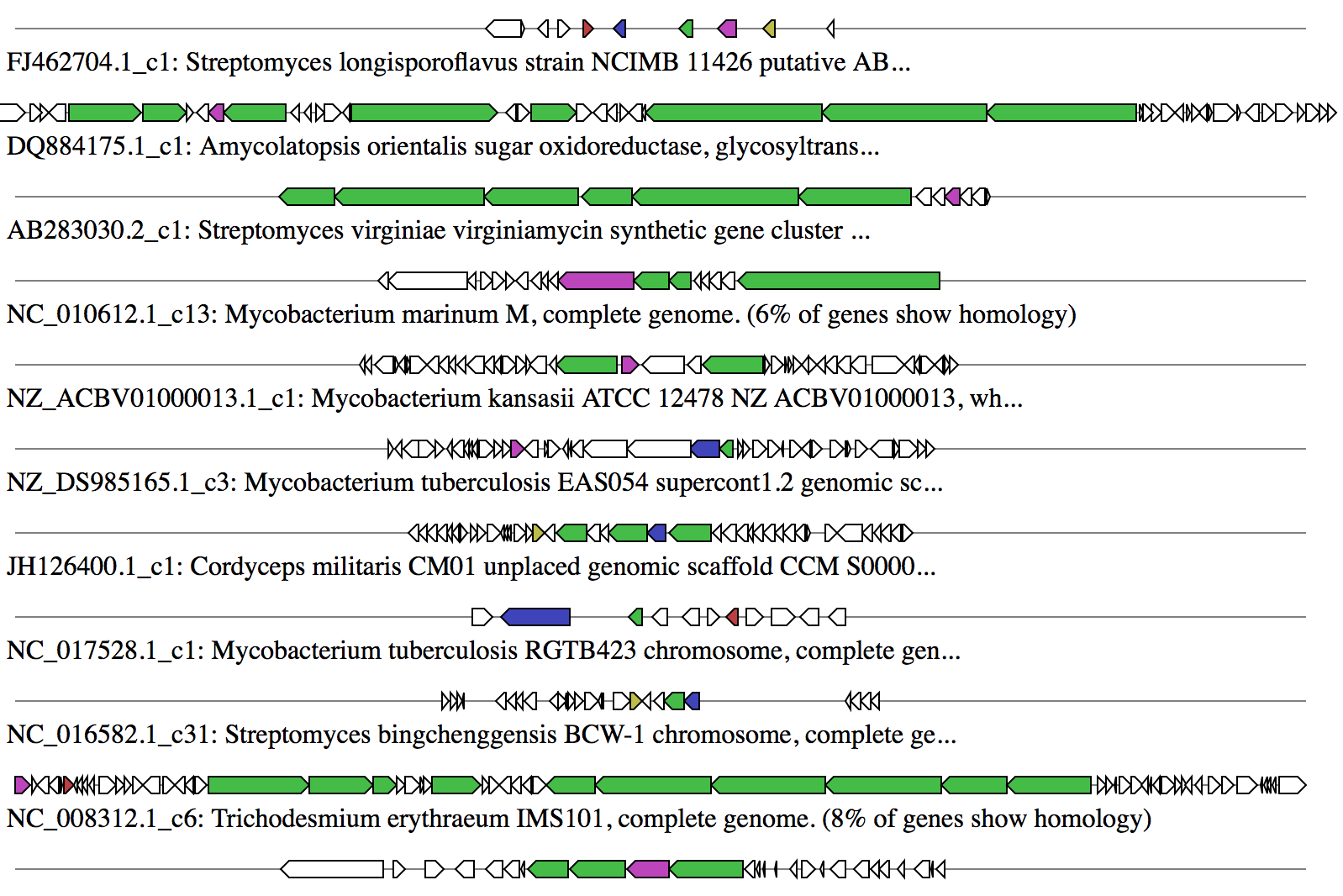
**

1. **mCaBGC47 - contig_427 – Nrps**

**
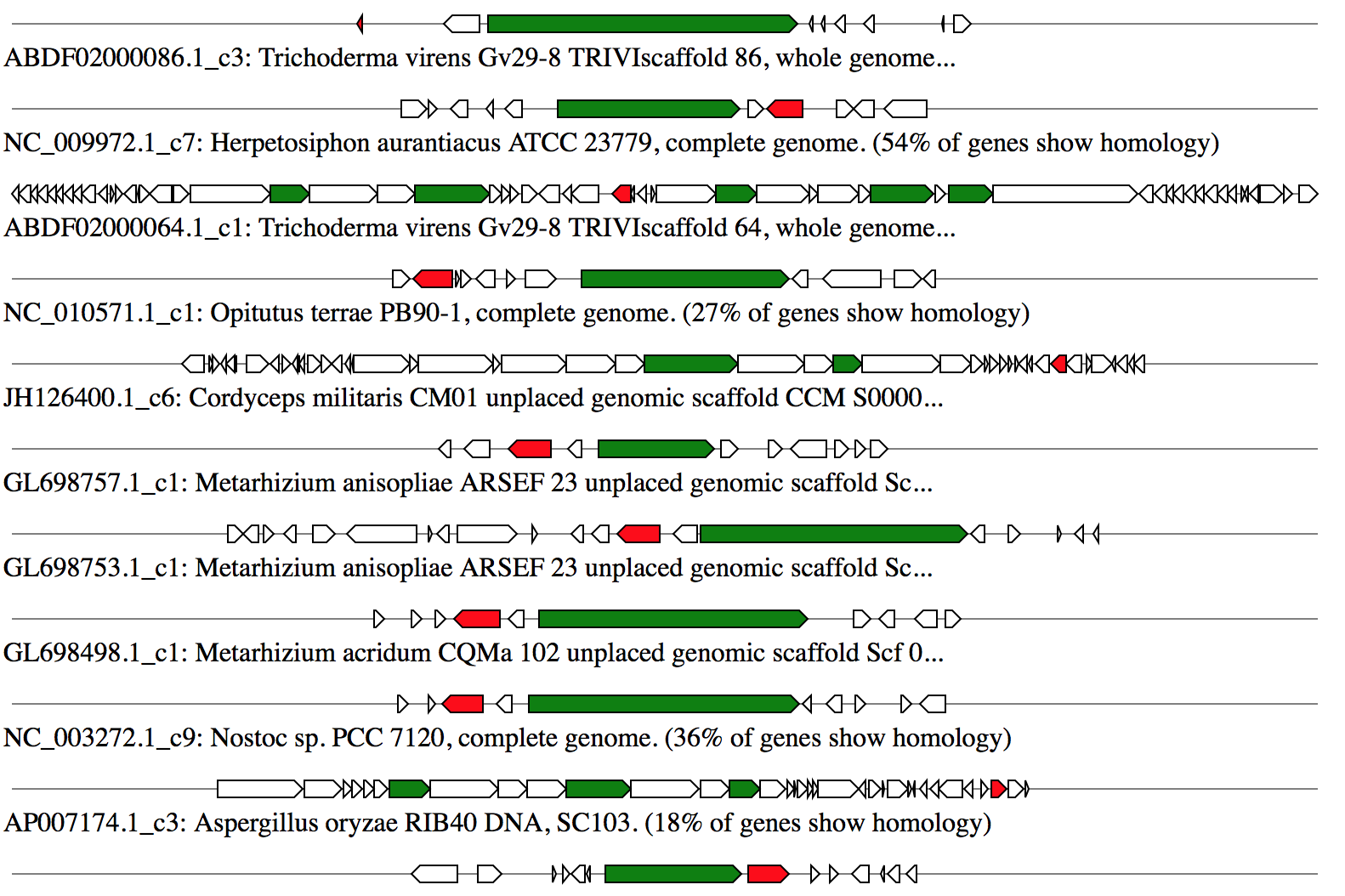
**

1. **mCaBGC48 - contig_437 - T1pks**

**
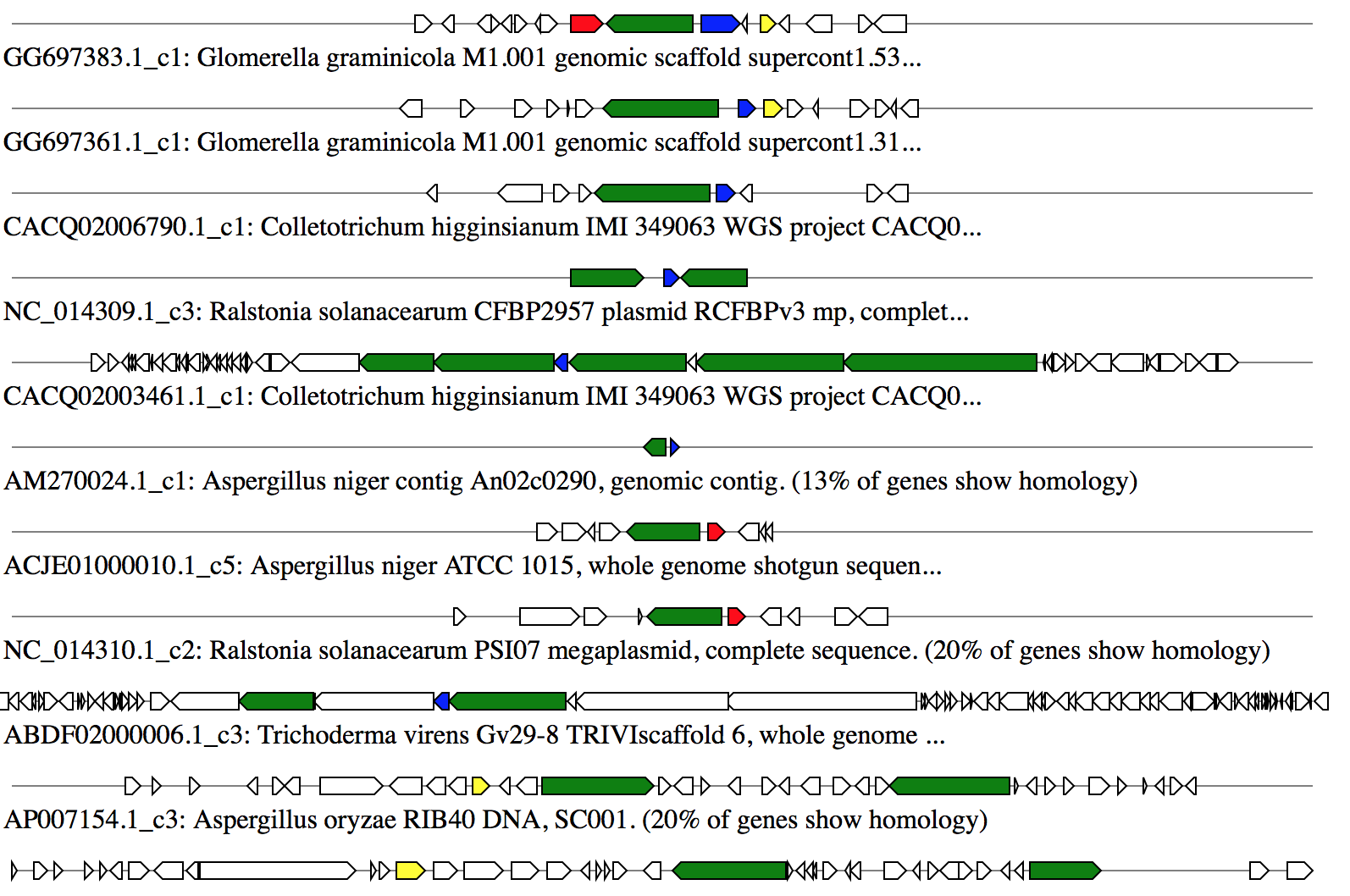
**

1. **mCaBGC49 - contig_441 – Nrps**

**
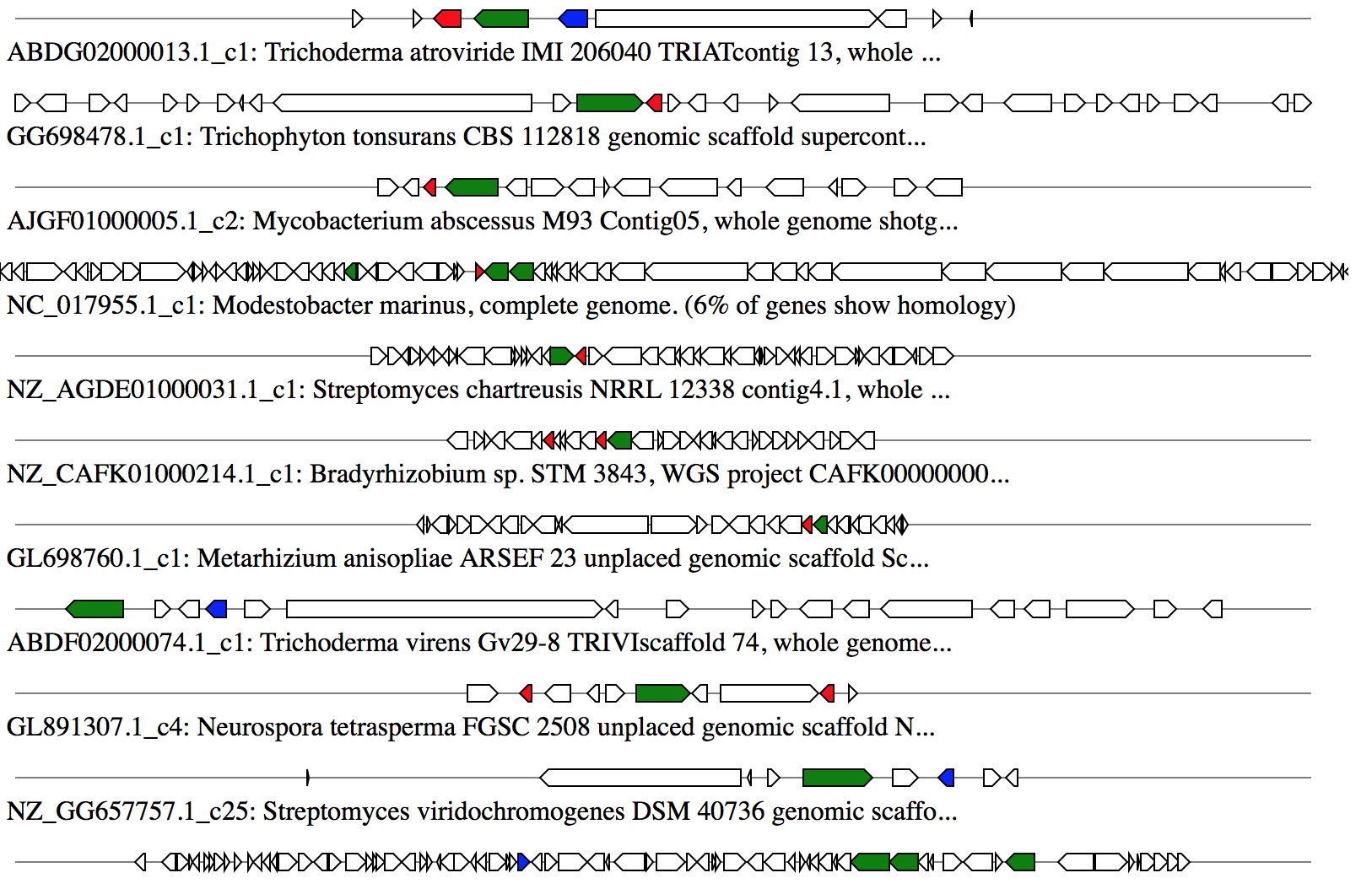
**

1. **mCaBGC50 - contig_451 – Terpene**

**
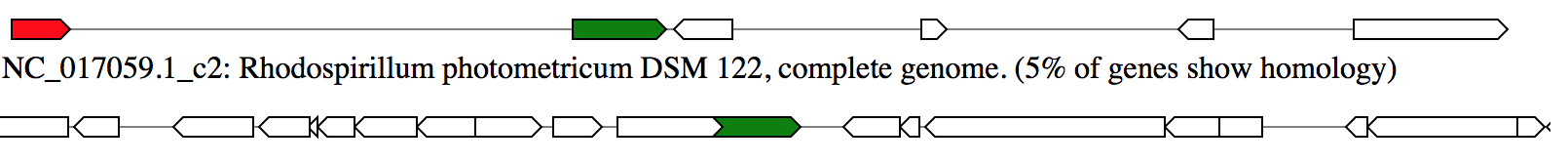
**

1. **mCaBGC51 - contig_461 – Nrps**

**
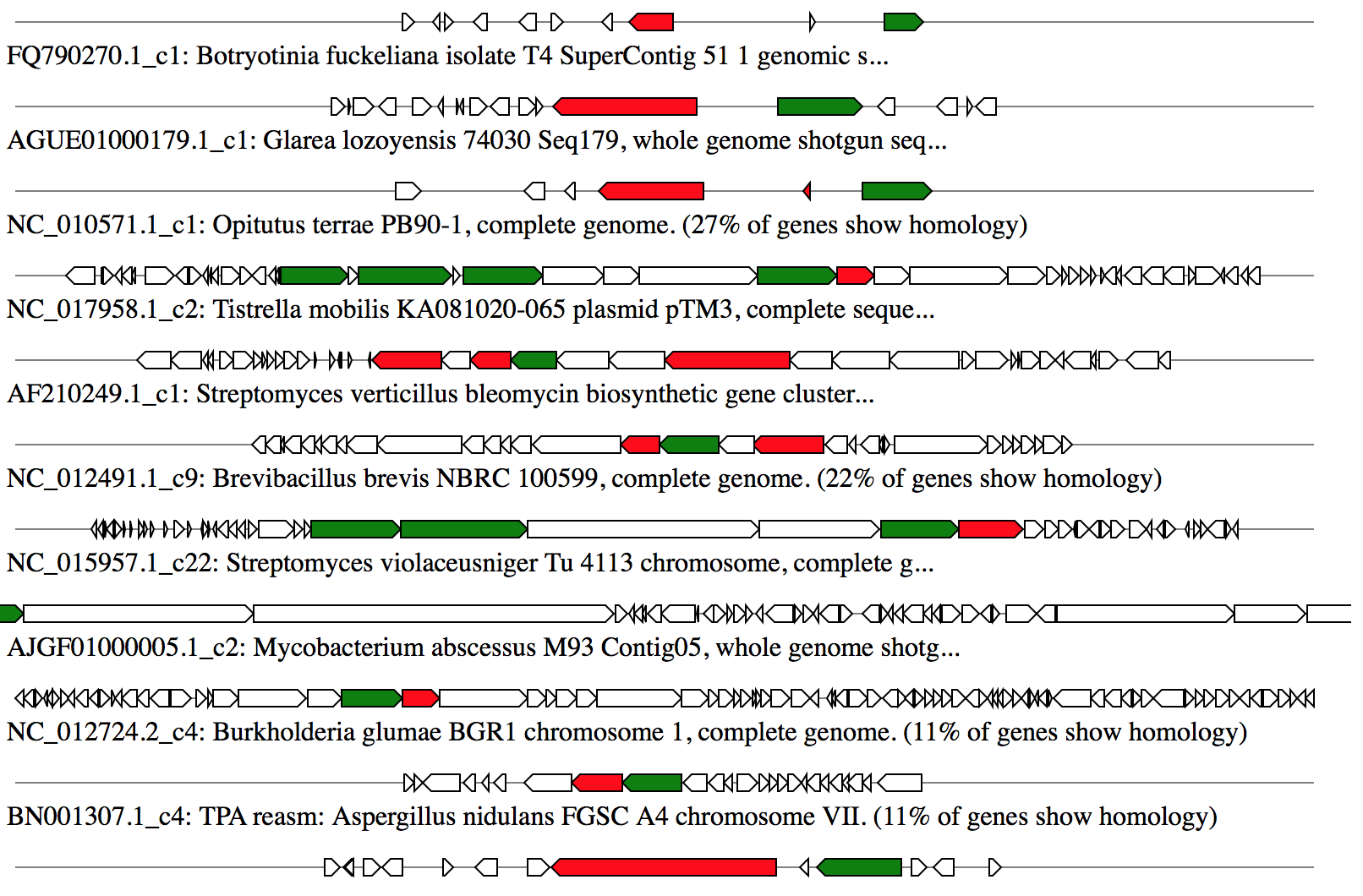
**

1. **mCaBGC52 - contig_584 - T1pks**

**
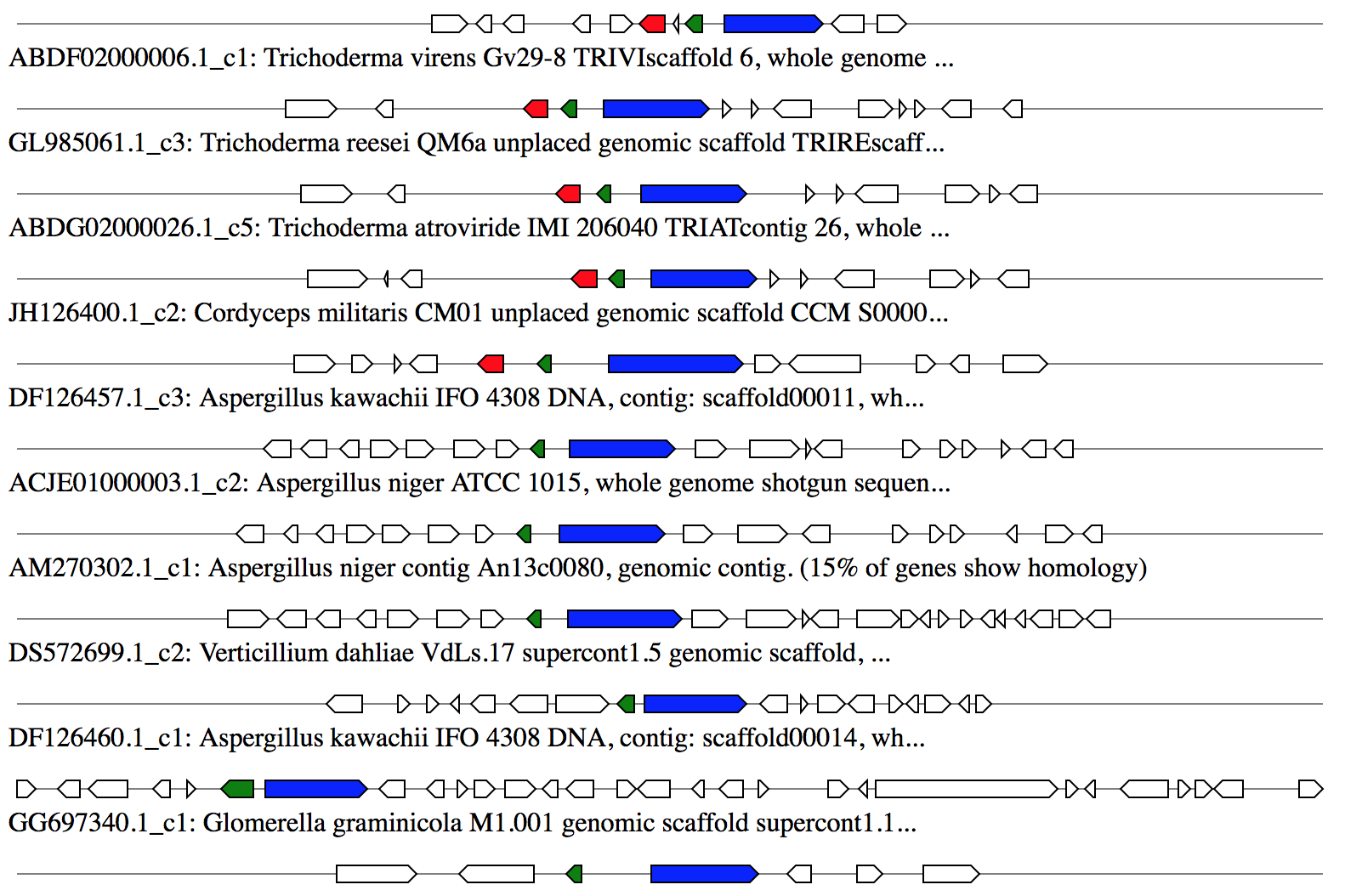
**

1. **mCaBGC53 - contig_711 – Other**

No Significant hit found.

1. **mCaBGC54 - contig_746 – Nrps**

**
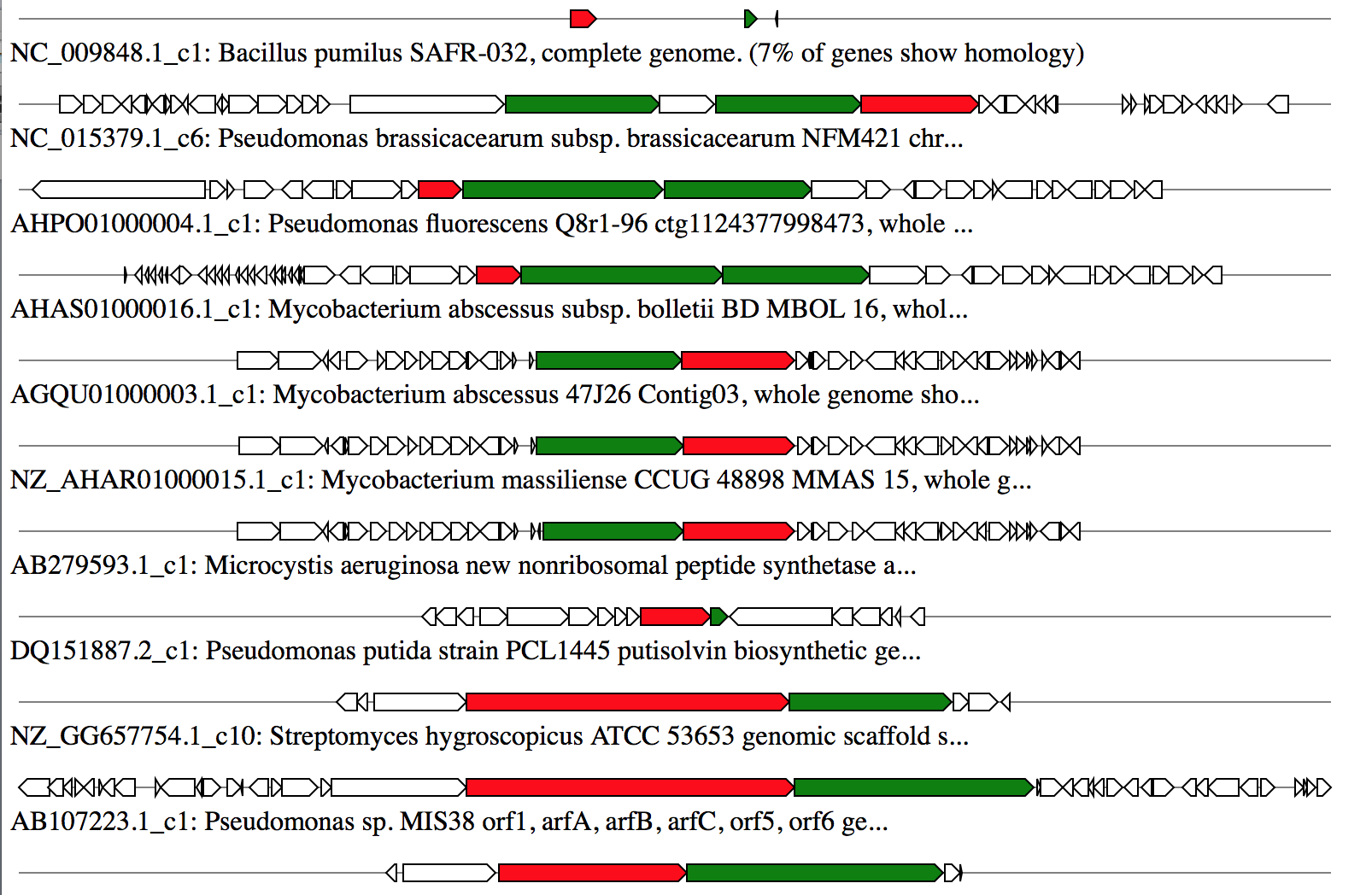
**

1. **mCaBGC55 - contig_858 – Terpene**

**
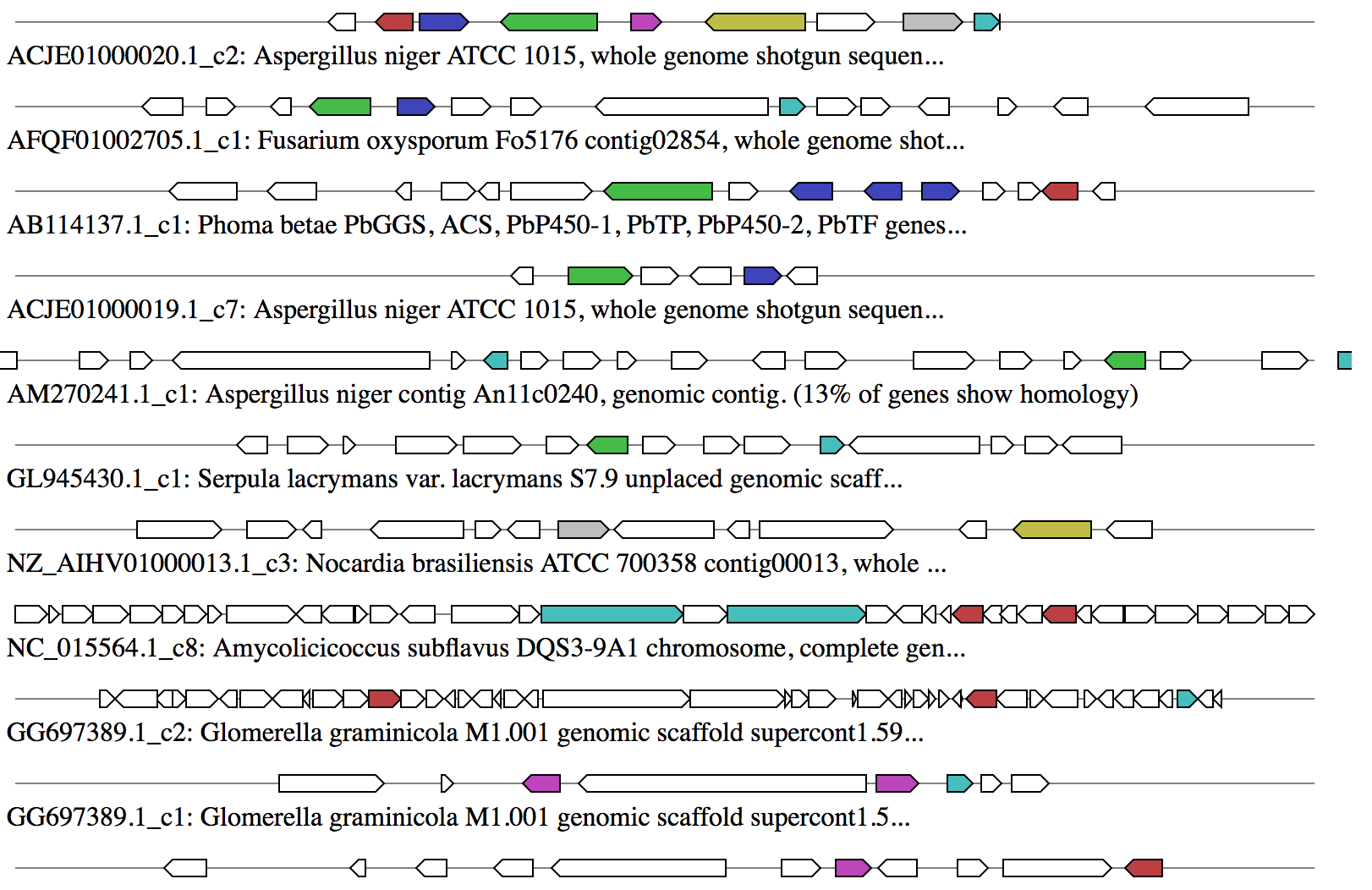
**

1. **mCaBGC56 - contig_865 – Nrps**

**
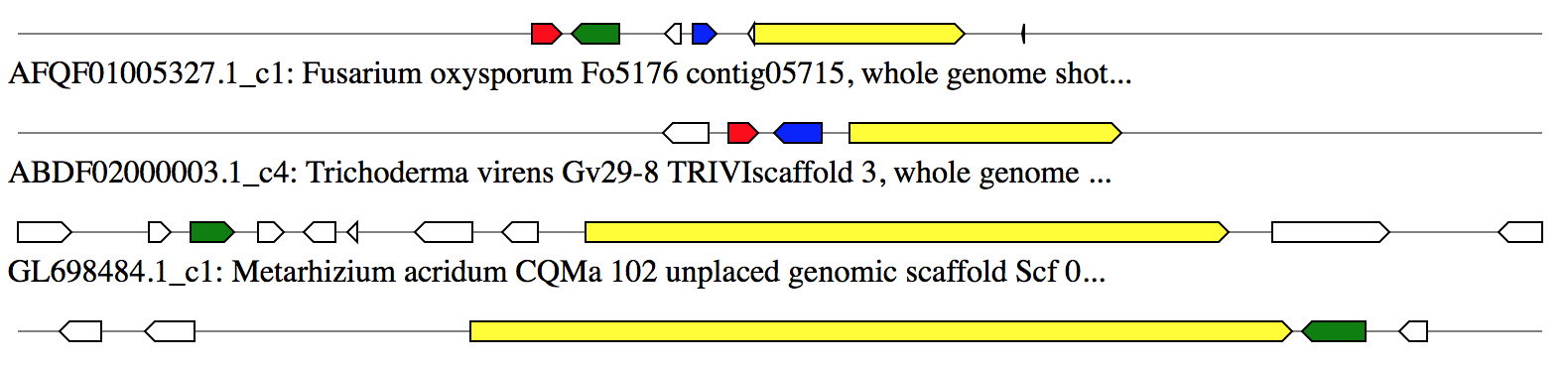
**

1. **mCaBGC57 - contig_907 - T1pks**

**
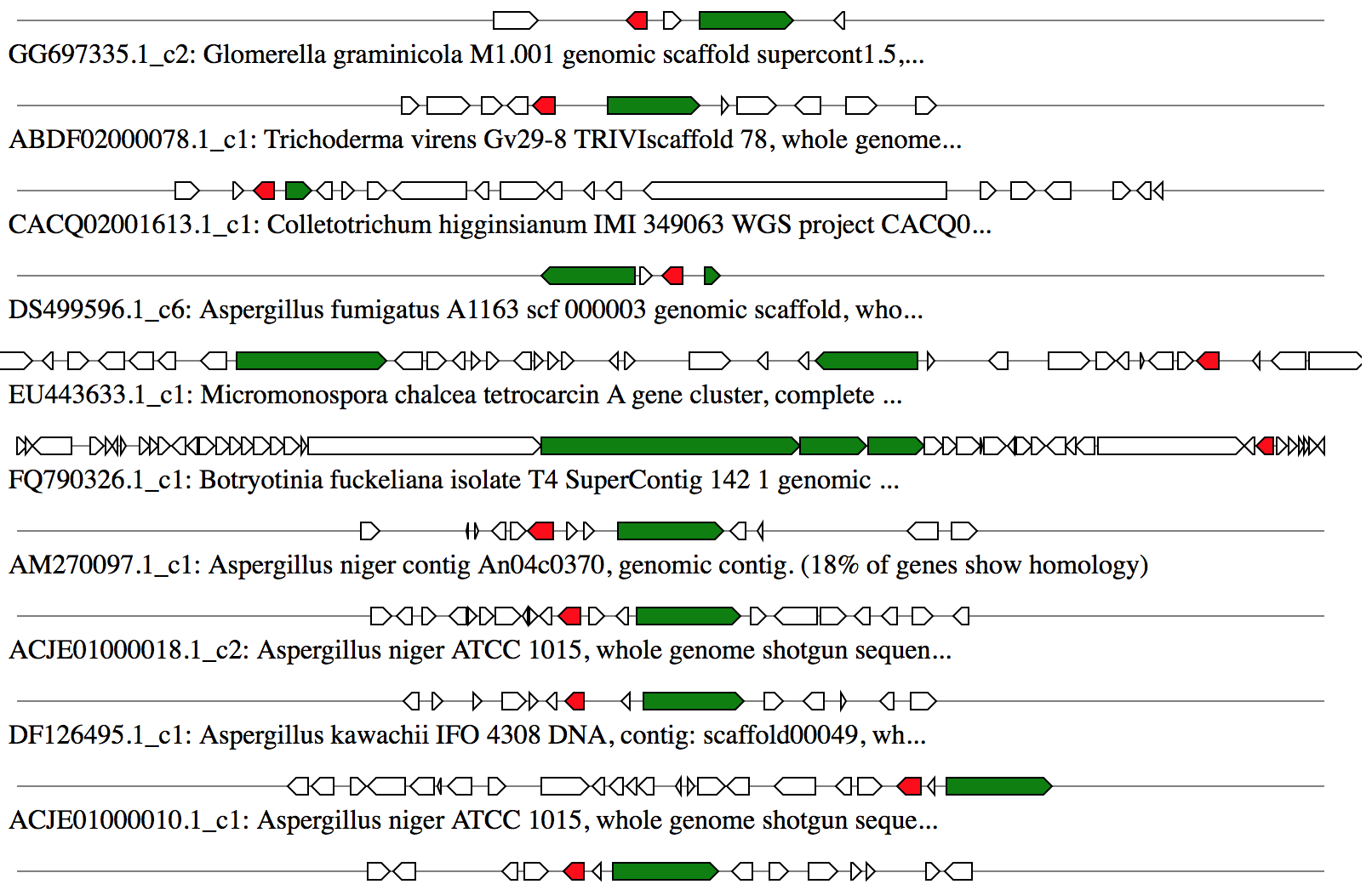
**

1. **mCaBGC58 - contig_964 - T1pks**

**
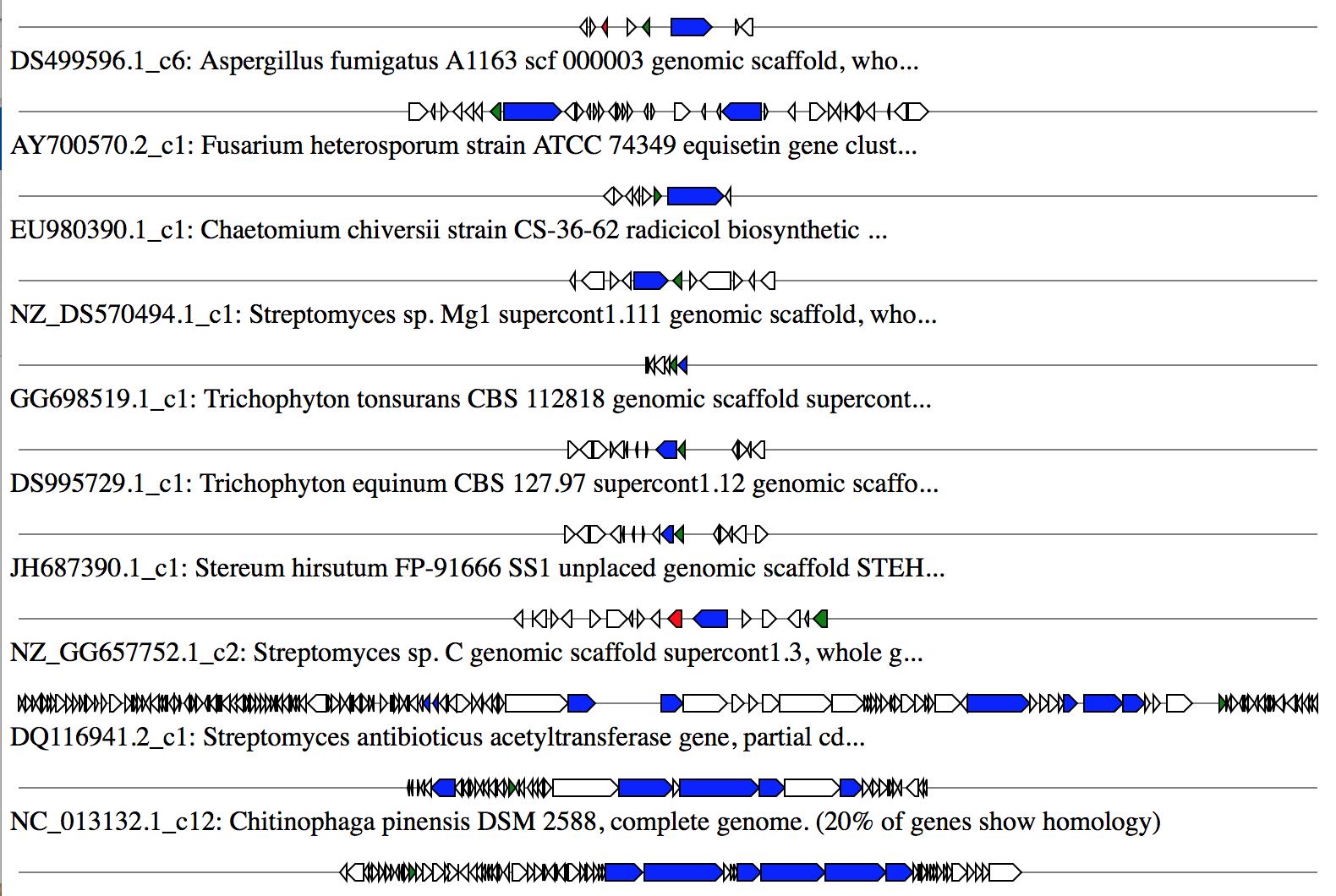
**

1. **mCaBGC59 - contig_965 - T1pks**

**
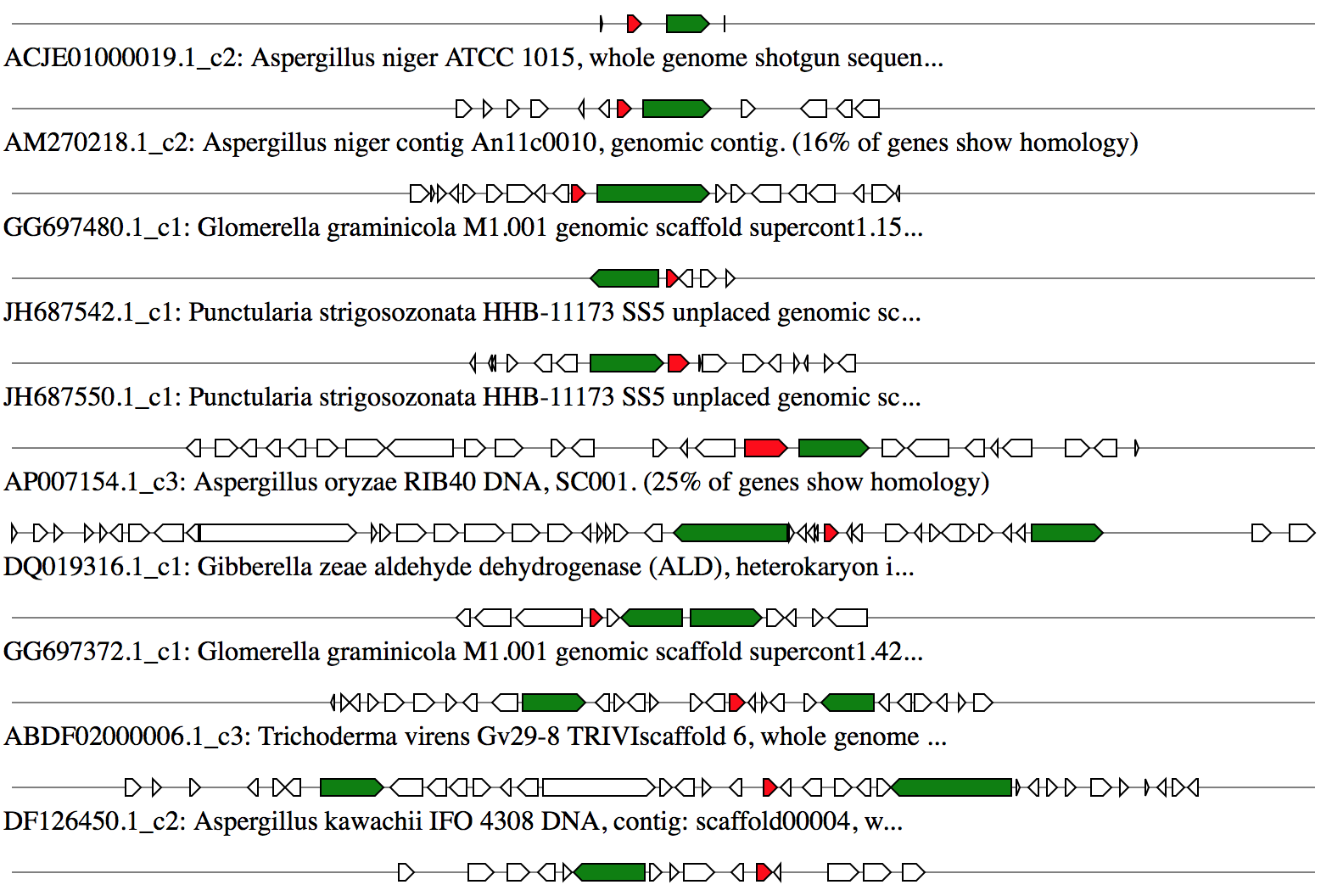
**

1. **mCaBGC60 - contig_1101 – Nrps**

No Significant hit found.

Fig. S4: Overview of 67 BGCs of *Pestalotiopsis* sp. KF079 genome and their homologous clusters in other species.

1. **mPeBGC1 Pesta-scaffold00001 T1pks-terpene**

**
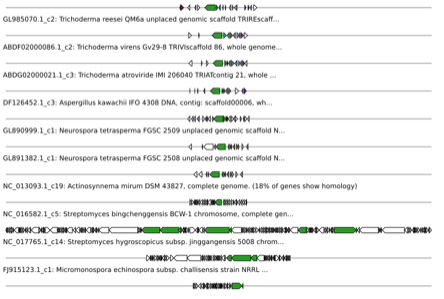
**

1. **mPeBGC2 Pesta-scaffold00001 T3pks**

**
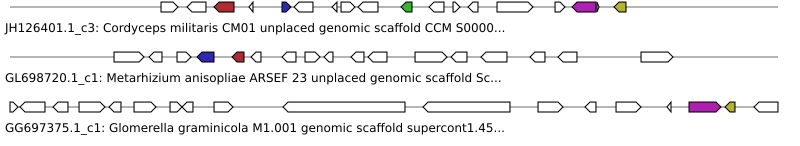
**

1. **mPeBGC3 Pesta-scaffold00001 Nrps**

**
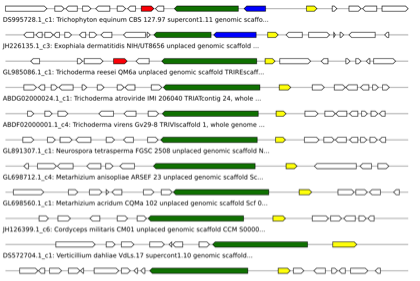
**

1. **mPeBGC4 Pesta-scaffold00002 T1pks**

**
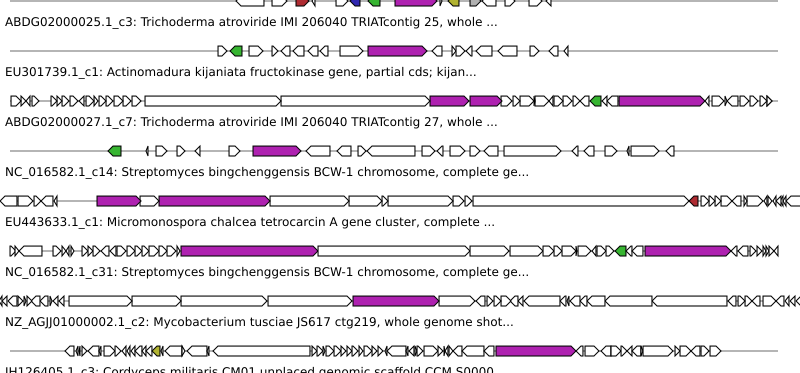
**

1. **mPeBGC5 Pesta-scaffold00002 Other**

**
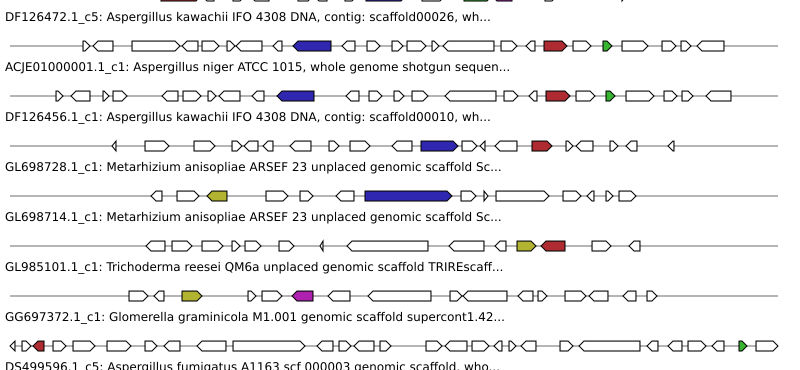
**

1. **mPeBGC6 Pesta-scaffold00004 T1pks**

**
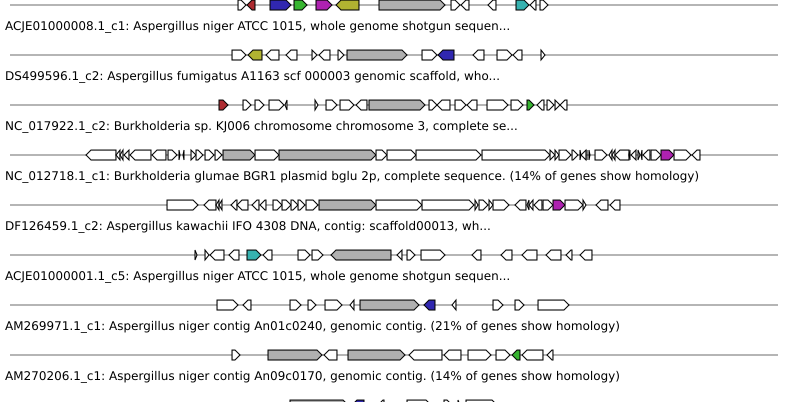
**

1. **mPeBGC7 Pesta-scaffold00005 Other**

**
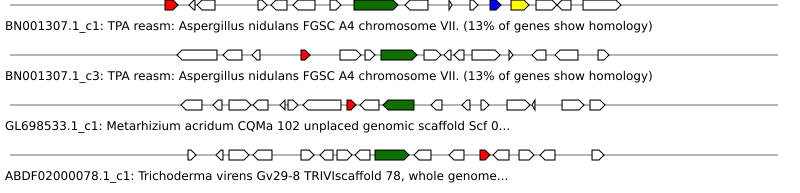
**

1. **mPeBGC8 Pesta-scaffold00007 T1pks**

**
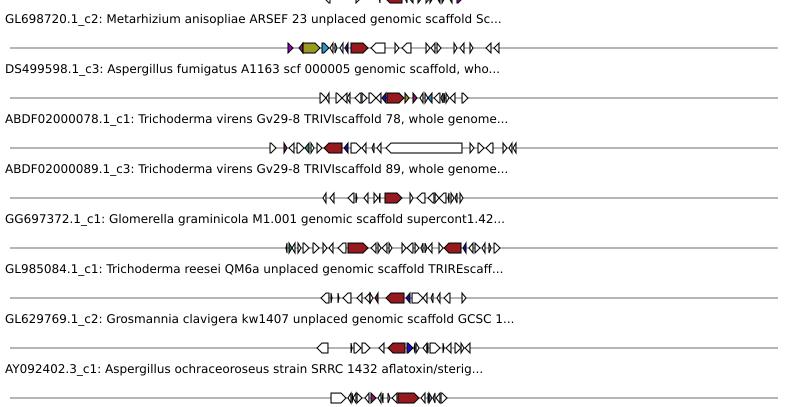
**

1. **mPeBGC9 Pesta-scaffold00007 Terpene**

**
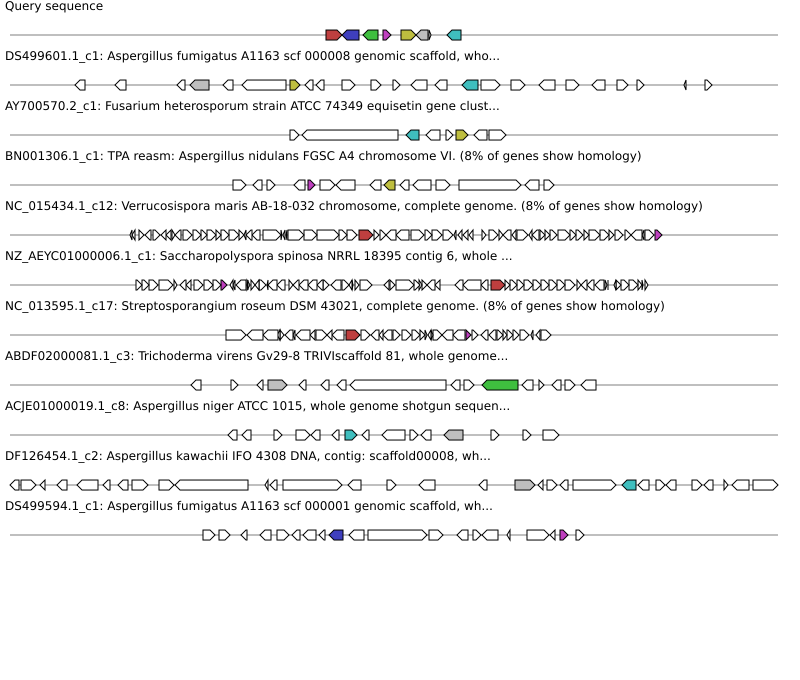
**

1. **mPeBGC10 Pesta-scaffold00008 Nrps**

**
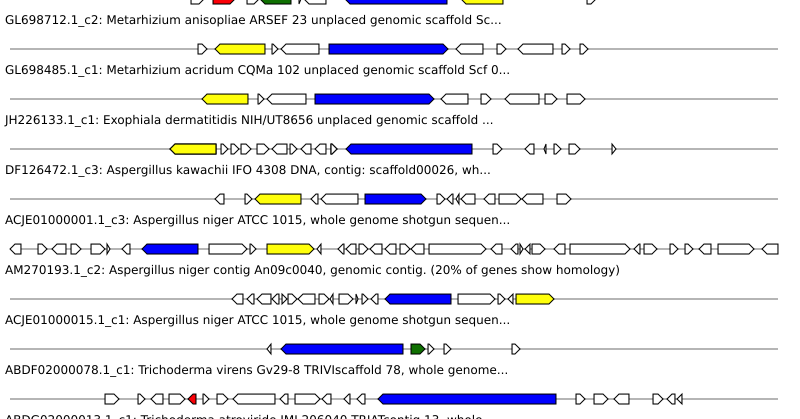
**

1. **mPeBGC11 Pesta-scaffold00009 Other**

**
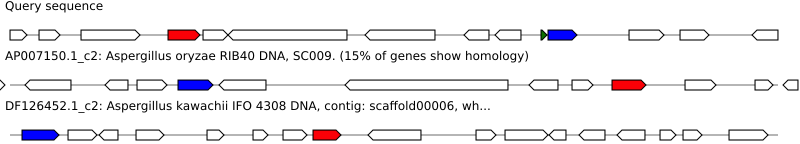
**

1. **mPeBGC12 Pesta-scaffold00009 T1pks**

**
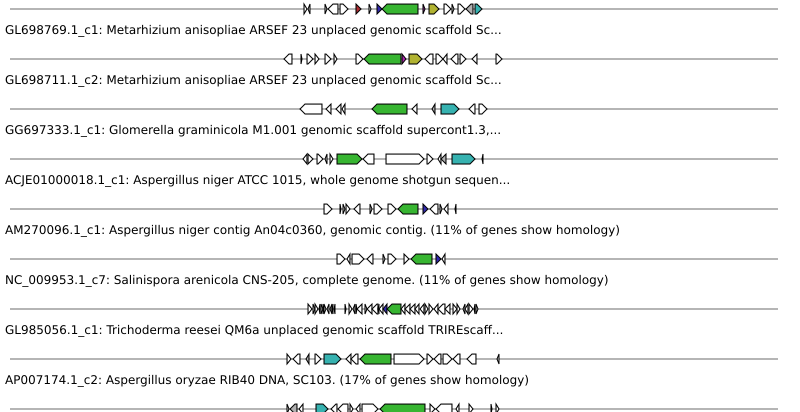
**

1. **mPeBGC13 Pesta-scaffold000010 T1pks**

**
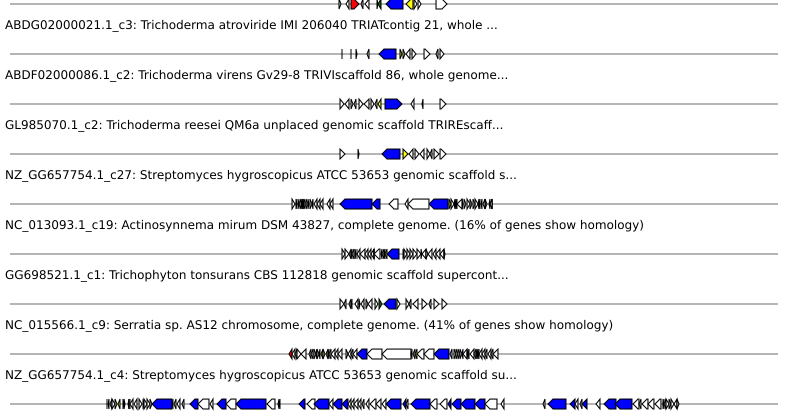
**

1. **mPeBGC14 Pesta-scaffold000013 Nrps-t1pks**

**
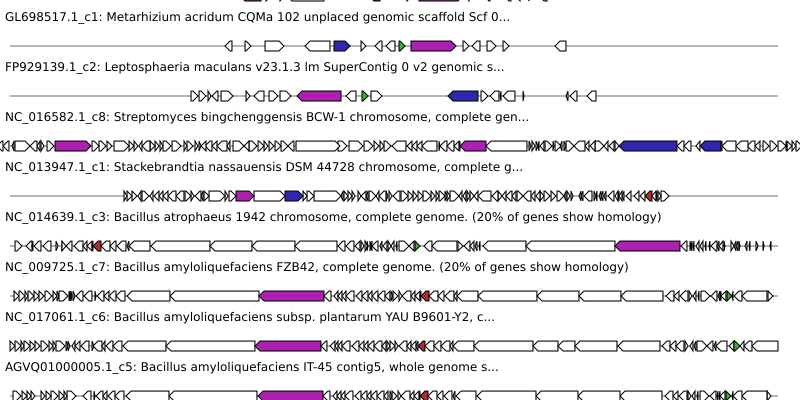
**

1. **mPeBGC15 Pesta-scaffold000014 Other**

**
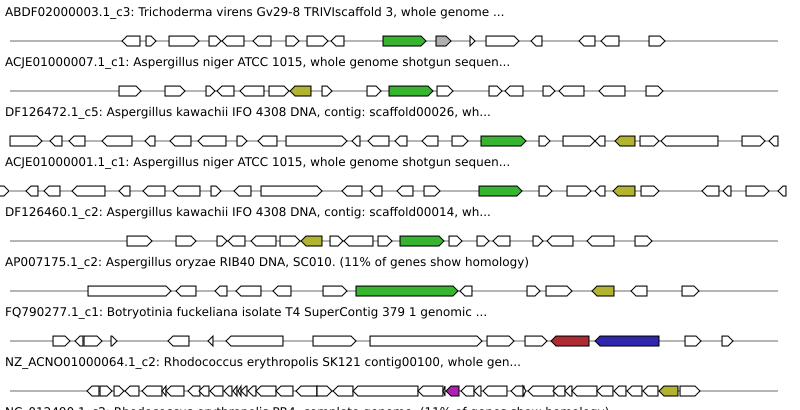
**

1. **mPeBGC16 Pesta-scaffold000014 T1pks**

**
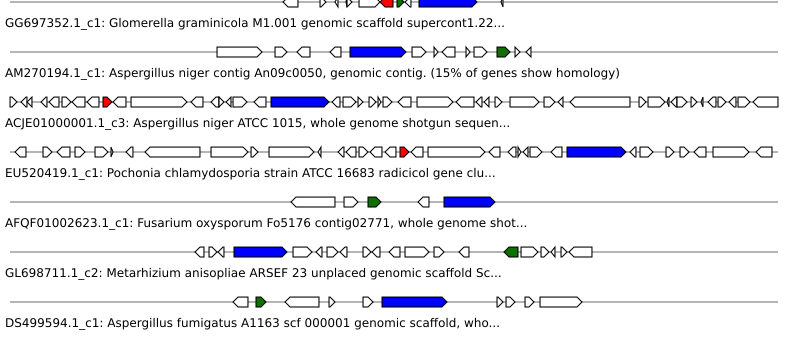
**

1. **mPeBGC17 Pesta-scaffold000016 Terpene**

**
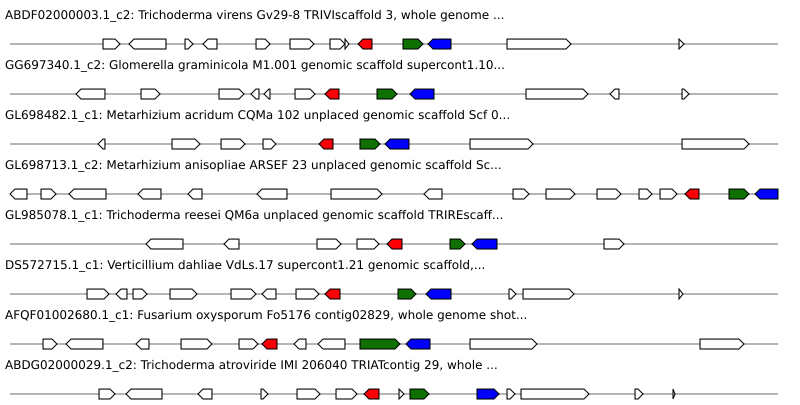
**

1. **mPeBGC18 Pesta-scaffold000016 Nrps**

**
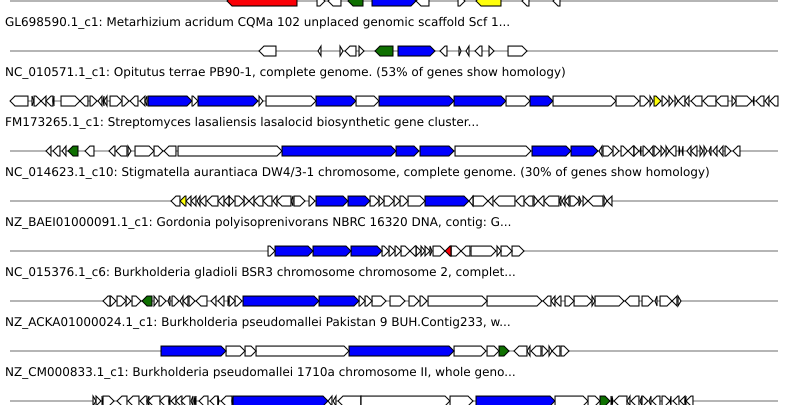
**

1. **mPeBGC19 Pesta-scaffold000016 T1pks**

**
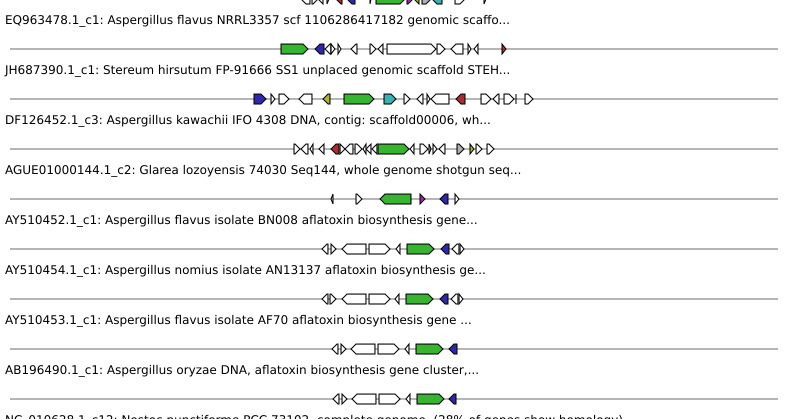
**

1. **mPeBGC20 Pesta-scaffold000018 Other**

**
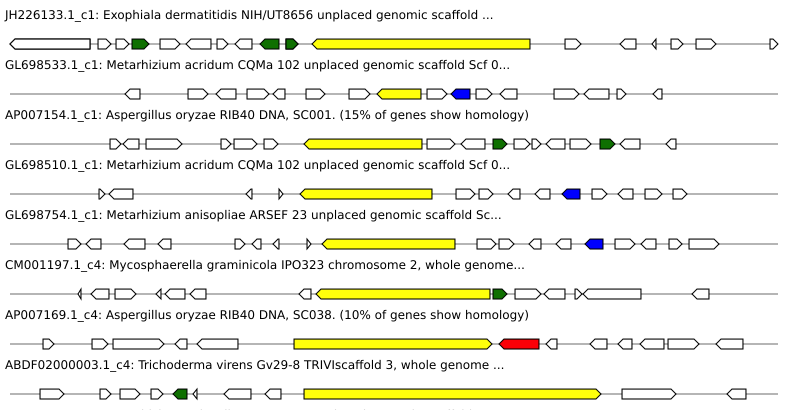
**

1. **mPeBGC21 Pesta-scaffold000019 Other**

**
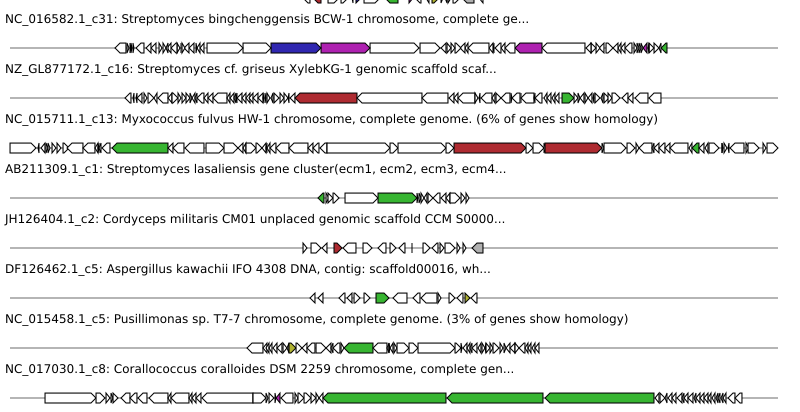
**

1. **mPeBGC22 Pesta-scaffold000023 Other**

**
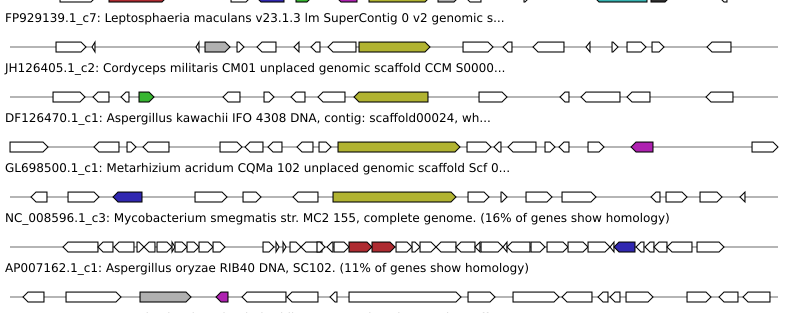
**

1. **mPeBGC23 Pesta-scaffold000023 Nrps**

**
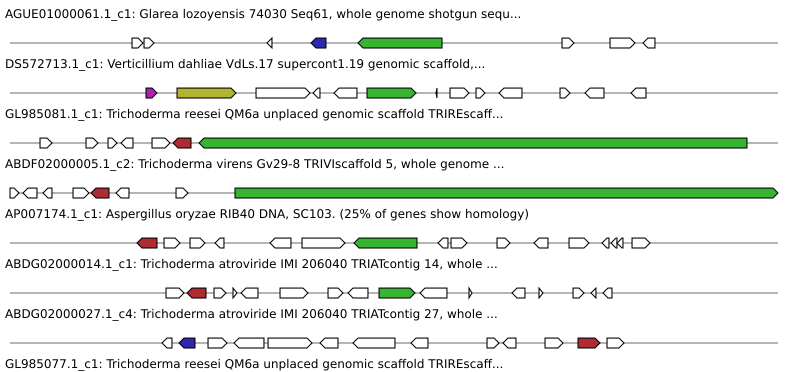
**

1. **mPeBGC24 Pesta-scaffold000025 Other**

**
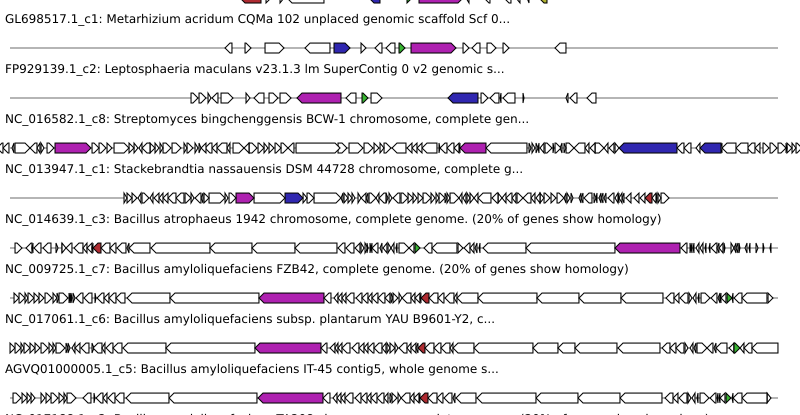
**

1. **mPeBGC25 Pesta-scaffold000025 Other**

**
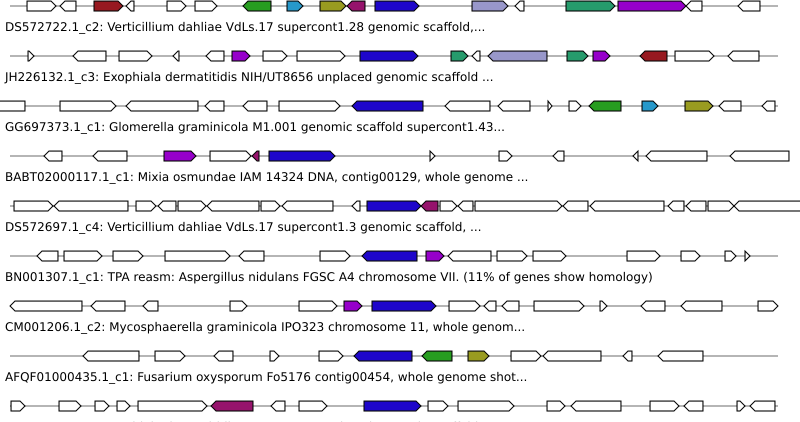
**

1. **mPeBGC26 Pesta-scaffold000027 T1pks**

**
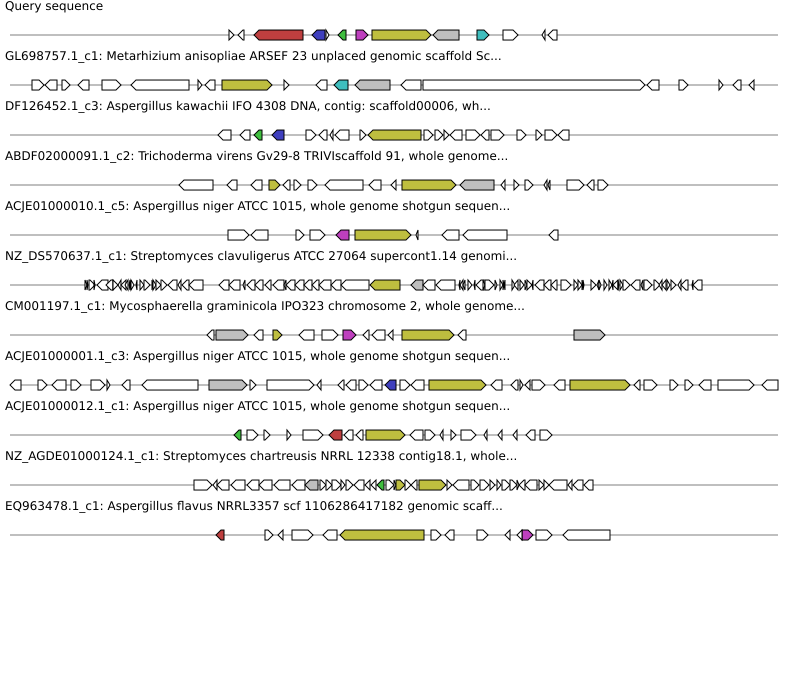
**

1. **mPeBGC27 Pesta-scaffold000027 Other**

**
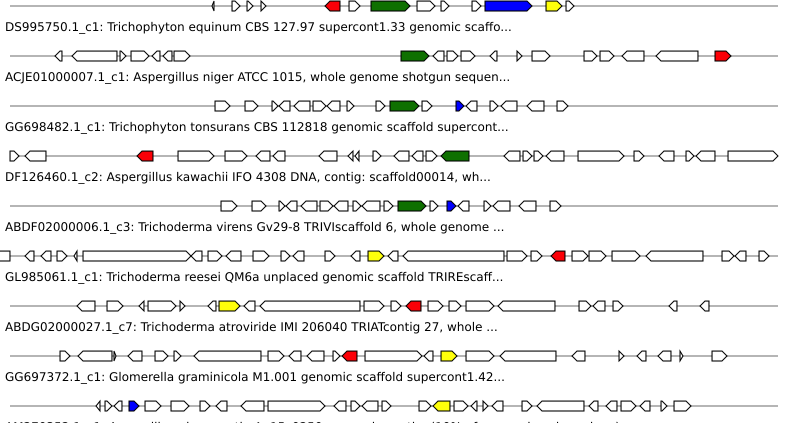
**

1. **mPeBGC28 Pesta-scaffold000029 Other**

**
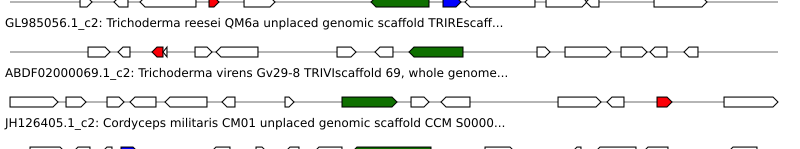
**

1. **mPeBGC29 Pesta-scaffold000031 Terpene**

**
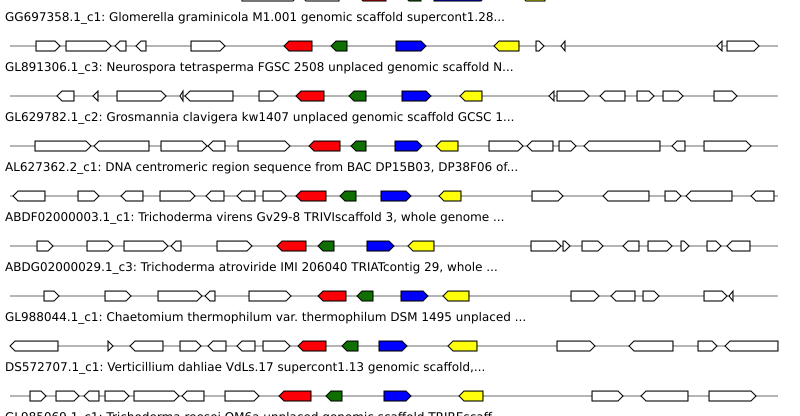
**

1. **mPeBGC30 Pesta-scaffold000033 Nrps**

**
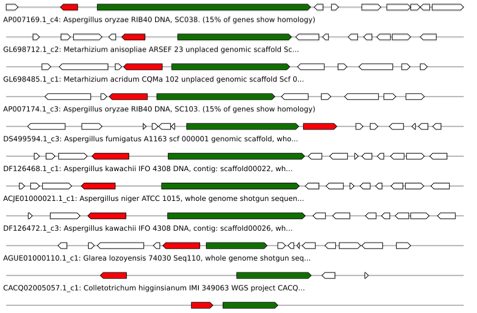
**

1. **mPeBGC31 Pesta-scaffold000036 Terpene**

**
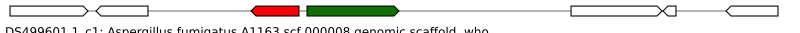
**

1. **mPeBGC32 Pesta-scaffold000036 Other**

**No homolog clusters found.**

1. **mPeBGC33 Pesta-scaffold000040 T1pks**

**
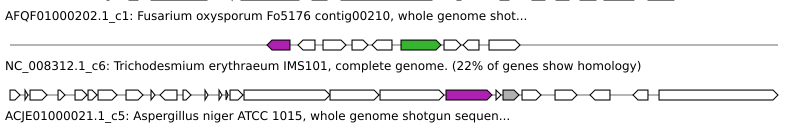
**

1. **mPeBGC34 Pesta-scaffold000041 T1pks**

**
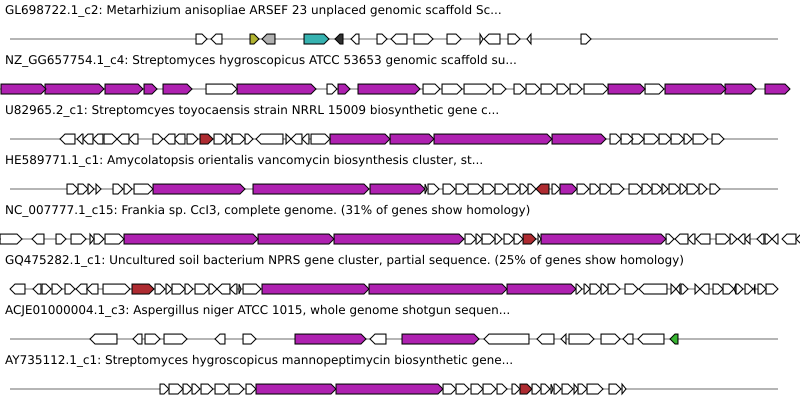
**

1. **mPeBGC35 Pesta-scaffold000042 Terpene**

**
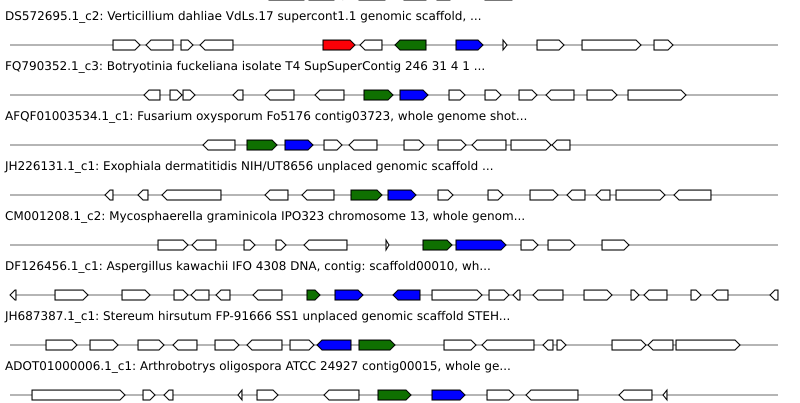
**

1. **mPeBGC36 Pesta-scaffold000043 Nrps**

**
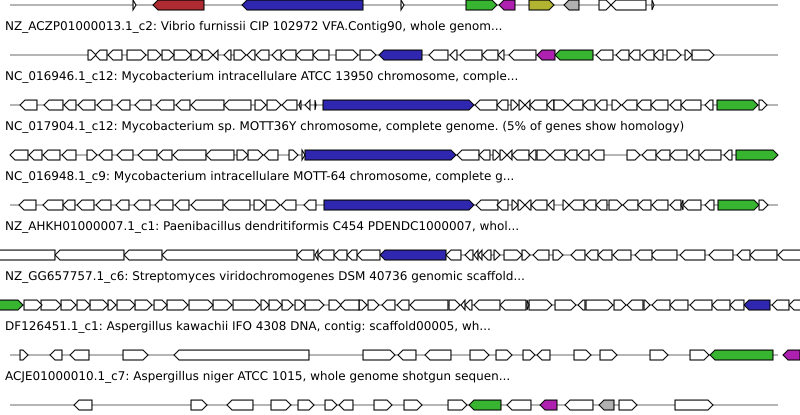
**

1. **mPeBGC37 Pesta-scaffold000043 Nrps**

**
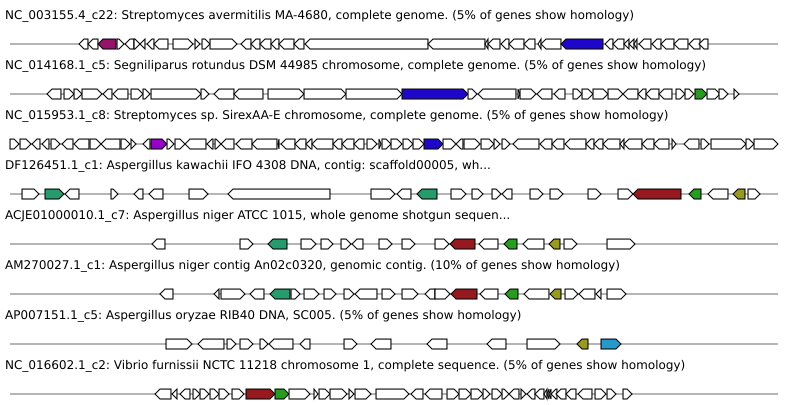
**

1. **mPeBGC38 Pesta-scaffold000048 T1pks**

**
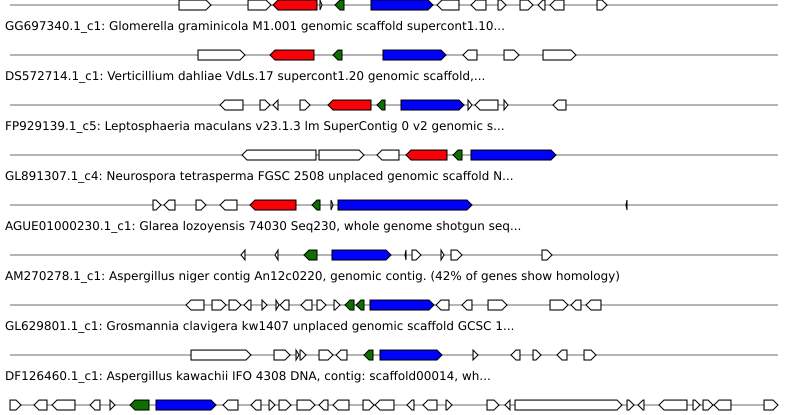
**

1. **mPeBGC39 Pesta-scaffold000049 Nrps-t1pks**

**
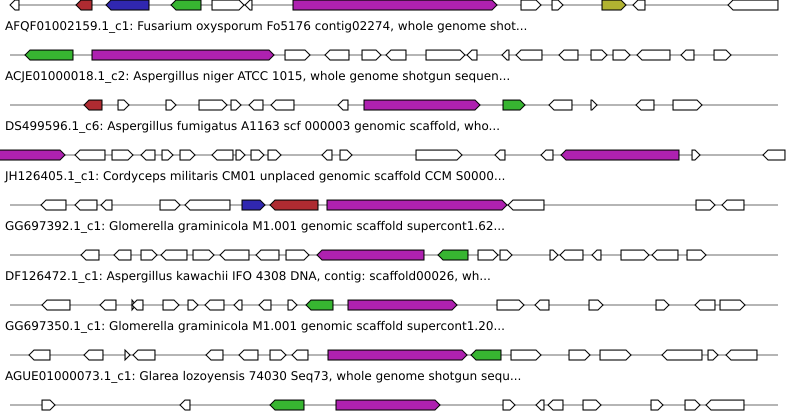
**

1. **mPeBGC40 Pesta-scaffold000051 Nrps-t1pks**

**
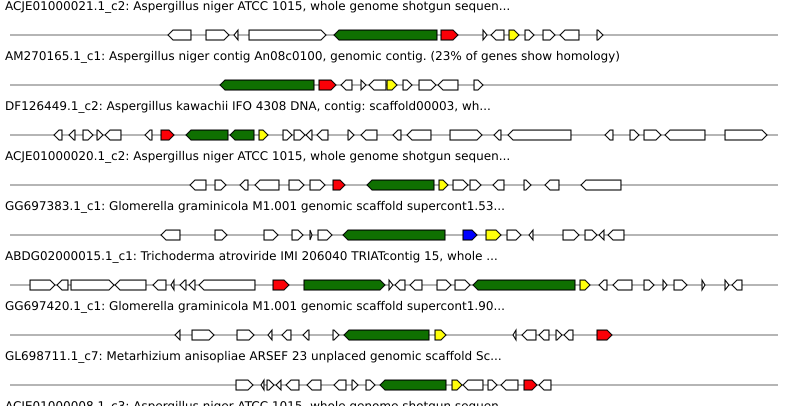
**

1. **mPeBGC41 Pesta-scaffold000052 Terpene**

**No homolog clusters found.**

1. **mPeBGC42 Pesta-scaffold000056 T1pks**

**
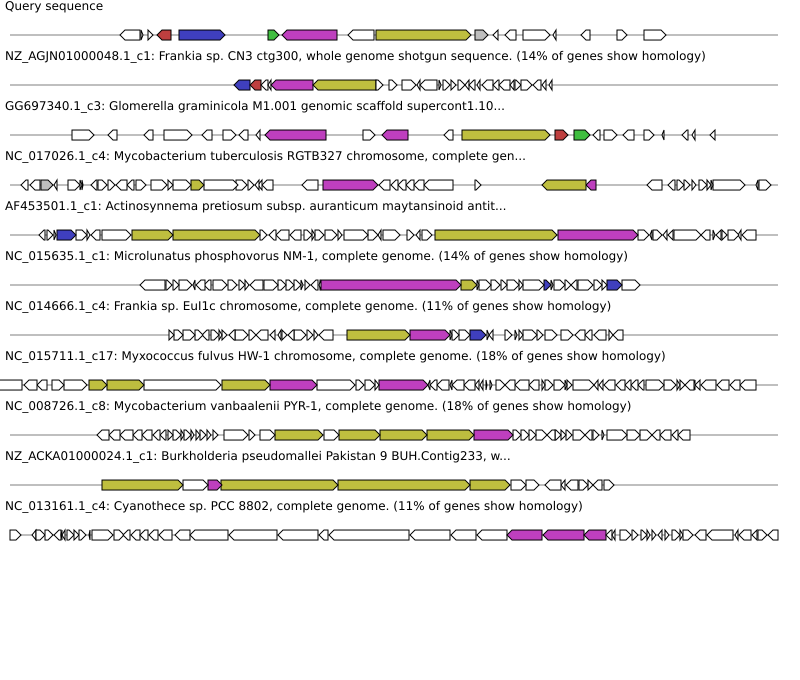
**

1. **mPeBGC43 Pesta-scaffold000057 Terpene**

**
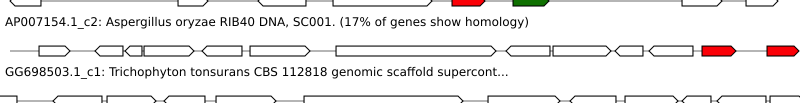
**

1. **mPeBGC44 Pesta-scaffold000062 T1pks**

**
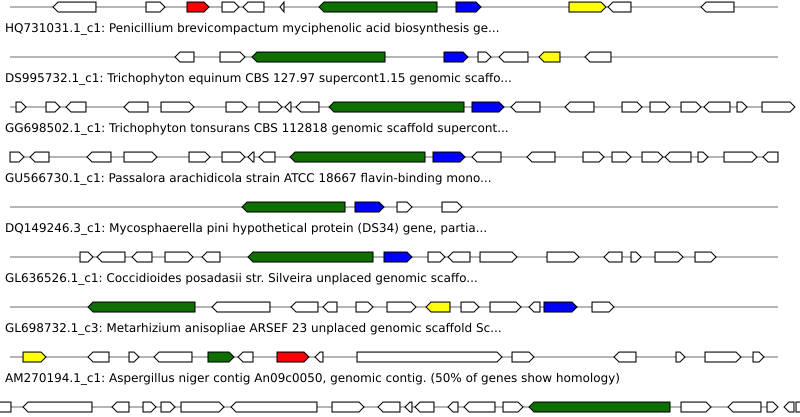
**

1. **mPeBGC45 Pesta-scaffold000062 T1pks**

1. **mPeBGC46 Pesta-scaffold000068 T1pks**

1. **mPeBGC47 Pesta-scaffold000069 Nrps**

1. **mPeBGC48 Pesta-scaffold000070 T1pks**

1. **mPeBGC49 Pesta-scaffold000071 Nrps**

1. **mPeBGC50 Pesta-scaffold000072 Terpene-t1pks**

1. **mPeBGC51 Pesta-scaffold000074 T1pks**

1. **mPeBGC52 Pesta-scaffold000077 T1pks**

1. **mPeBGC53 Pesta-scaffold000077 T1pks**

1. **mPeBGC54 Pesta-scaffold000078 T1pks**

1. **mPeBGC55 Pesta-scaffold000085 Lantipeptide**

1. **mPeBGC56 Pesta-scaffold000089 Other**

1. **mPeBGC57 Pesta-scaffold000091 Other**

1. **mPeBGC58 Pesta-scaffold000104 Other**

1. **mPeBGC59 Pesta-scaffold000105 Nrps**

1. **mPeBGC60 Pesta-scaffold000115 T1pks**

1. **mPeBGC61 Pesta-scaffold000116 Nrps**

1. **mPeBGC62 Pesta-scaffold000133 Nrps**

1. **mPeBGC63 Pesta-scaffold000156 Terpene**

1. **mPeBGC64 Pesta-scaffold000158 Hglks-t1pks**

1. **mPeBGC65 Pesta-scaffold000160 Other**

**No homolog clusters found.**

1. **mPeBGC66 Pesta-scaffold000173 Terpene**

**No homolog clusters found.**

1. **mPeBGC67 Pesta-scaffold000184 T1pks**

**Fig. S5: Multiple alignment of the HMG-domain mating-type protein MAT1-2-1 from *Calcarisporium sp.* KF525.** Clac (*Calcarisporium sp.*; contig_519.g12283.t1) with homologs from other ascomycetes. Abbreviations and accession numbers: Efes (*Epichloe festucae*; AEI72619.1), Macr (*Metarhizium acridum*; EFY88585.1), Osin (*Ophiocordyceps sinensis*; AGW27541.1), Tatr (*Trichoderma atroviride*; EHK42953.1), Tvir (*Trichoderma virens*; EHK23194.1), Cmil (*Cordyceps militaris*; BAC66500.1), Iten (*Isaria tenuipes*; BAC66503.1), Ffuj (*Fusarium fujikuroi*; AAC71056.1), Fnyg (*Fusarium nygamai*; AEP03823.1), Foxy (*Fusarium oxysporum*; ENH71076.1). The conserved HMG box is indicated by a grey bar.

**Fig. S6: Multiple alignment mating-type protein MAT1-2-3 from *Calcarisporium sp.* KF525.** Clac (*Calcarisporium sp.*; contig_519.g12284.t1) with homologs from other ascomycetes. Abbreviations and accession numbers: Fman (Fusarium mangiferae; AEP03839.1), Ffuj (*Fusarium fujikuroi*; AEP03799.1), Fsac (Fusarium sacchari, AEP03794.1), Fnyg (*Fusarium nygamai*; AEP03824.1), Foxy (*Fusarium oxysporum*; ENH71077.1), Tatr (*Trichoderma atroviride*; EHK42952.1), Tvir (*Trichoderma virens*; EHK22526.1), Macr (*Metarhizium acridum*; EFY88586.1), Osin (*Ophiocordyceps sinensis*; EQL04084.1).

Fig. S7: Phylogenetic tree of sugar transporters for *Calcarisporium sp.* KF525. A phylogenetic tree was created using protein sequences from *Calcarisporium* MFS-type transporters belonging to the Sugar Porter family (2.A.1.1) and transporters of the same group from *N. crassa*. The transporter sequences of *Calcarisporium* were submitted to Batch Conserved Domains search (NCBI) and domains unrelated to transporters were removed. The corresponding truncated sequences were flagged by the letter “P” (for “partial”). LacY from *E. coli* was used as an outgroup. *N. crassa* transporters were colored based on published functional data and sub-groups were created based on this information.

**Fig. S8:** **Phylogenetic tree of sugar** **transporters for *Pestalotiopsis* sp. KF079.** Further details were same as of Fig. S7.
