# Supplementary material for: Genome Sequencing and analyses of Two Marine Fungi from the North Sea Unraveled a Plethora of Novel Biosynthetic Gene Clusters
Source: Sci Rep. 2018 Jul 5;8:10187. doi: 10.1038/s41598-018-28473-z (PMC6033941; doi:10.1038/s41598-018-28473-z)
Supplement: Supplementary file 2 — TableS1 [file 41598_2018_28473_MOESM2_ESM.docx]

**Table S1**

**Genome Sequencing and analyses of Two Marine Fungi from the North Sea Unraveled a Plethora of Novel Biosynthetic Gene Clusters**

**Abhishek Kumar^1^, Jens Laurids Sørensen^2^, Frederik Teilfeldt Hansen^3^, Mikko Arvas^4^, Muhammad Fahad Syed^4, 5^, Lara Hassan^6^, J. Philipp Benz^6^, Eric Record^7,8^,**

**Bernard Henrissat^8,9,10^, Stefanie Pöggeler^11^, and Frank Kempken^1, *^**

^1^Department of Genetics & Molecular Biology in Botany, Institute of Botany,

Christian-Albrechts-University at Kiel, Kiel, Germany

^2^Department of Chemistry and Bioscience, Aalborg University, Niels Bohrs Vej 8, DK-6700 Esbjerg, Denmark

^3^Department of Biochemistry, McGill University, Francesco Bellini Life Sciences Building, 3649 Promenade Sir William Osler, Montreal, QC, H3G 0B1, Canada

1. ^4^VTT Technical Research Centre of Finland Ltd, Tietotie 2, FI-02044 VTT, Espoo, Finland

^5^Biocomputing Platforms Ltd, Tekniikantie 14, FI-02150 Espoo, Finland

^6^Holzforschung München, TUM School of Life Sciences Weihenstephan, Technische Universität München, Hans-Carl-von-Carlowitz-Platz 2, Freising, Germany

^7^ INRA, Aix-Marseille Université, UMR1163, Biodiversité et Biotechnologie Fongiques, Marseille, France

^8^Centre National de la Recherche Scientifique (CNRS), UMR7257, Université Aix-Marseille, Marseille, 13288, France

^9^INRA, USC 1408 AFMB, F-13288 Marseille, France

^10^Department of Biological Sciences, King Abdulaziz University, Jeddah, Saudi Arabia

^11^Institute of Microbiology and Genetics, Department of Genetics of Eukaryotic Microorganisms, Georg-August University, Göttingen, Germany

***Corresponding author:**

**Frank Kempken,** Department of Genetics & Molecular Biology in Botany, Institute of Botany, Christian-Albrechts-University at Kiel, Kiel, Germany, [fkempken@bot.uni-kiel.de](mailto:fkempken@bot.uni-kiel.de)

**Email of other authors:**

AK – [abhishek.abhishekkumar@gmail.com](mailto:abhishek.abhishekkumar@gmail.com);

BH – [bernard.henrissat@afmb.univ-mrs.fr](mailto:bernard.henrissat@afmb.univ-mrs.fr);

ER – [eric.record@inra.fr](mailto:eric.record@inra.fr);

JLS – [jls@bio.aau.dk](mailto:jls@bio.aau.dk);

JFTH - [jens.hansen@mail.mcgill.ca](mailto:jens.hansen@mail.mcgill.ca);

JPB - [benz@hfm.tum.de](mailto:benz@hfm.tum.de);

LH - hassan@hfm.tum.de;

MA – [mikko.arvas@veripalvelu.fi](mailto:mikko.arvas@veripalvelu.fi);

MFS – [s_m_fahad@yahoo.com](mailto:s_m_fahad@yahoo.com);

SP – [spoegge@gwdg.de](mailto:spoegge@gwdg.de);

**Keywords**

Biosynthetic gene clusters; Calcarisporium; CAZyome; Marine fungi; Marine fungal genomics; Pestalotiopsis; Secondary metabolites; Transporters; Transcriptomics

**Table S1. Repeat contents in assembled genomes of two marine fungal strains.**

| **Repeat type** | ***Calcarisporium* sp. KF525** | | | ***Pestalotiopsis* sp. K079** | | |
| --- | --- | --- | --- | --- | --- | --- |
|  | **No. of**  **elements*** | **Length (bp)** | **%Genome** | **No. of**  **elements*** | **Length (bp)** | **%Genome** |
| **Retroelements** | **170** | **69031** | **0.19** | **254** | **103427** | **0.22** |
| **LINEs** | **35** | **26402** | **0.07** | **6** | **299** | **0.00** |
| **R1/LOA/Jockey** | **3** | **288** | **0.00** | **---** | **---** | **---** |
| **LTR elements** | **135** | **42629** | **0.12** | **248** | **103128** | **0.22** |
| **Ty1/Copia** | **52** | **10915** | **0.03** | **27** | **2681** | **0.01** |
| **Gypsy/DIRS1** | **83** | **31714** | **0.09** | **221** | **100447** | **0.22** |
| **DNA transposons** | **56** | **16380** | **0.05** | **33** | **2302** | **0.00** |
| **hobo-Activator** | **4** | **236** | **0.00** | **3** | **254** | **0.00** |
| **Tc1-IS630-Pogo** | **31** | **10720** | **0.03** | **2** | **361** | **0.00** |
| **PiggyBac** | **1** | **77** | **0.00** | **---** | **---** | **---** |
| **Tourist/Harbinger** | **4** | **273** | **0.00** | **6** | **367** | **0.00** |
| **Unclassified** | **2** | **187** | **0.00** | **1** | **127** | **0.00** |
| **Total interspersed repeats** |  | **85598** | **0.24** |  | **105856** | **0.23** |
| **Small RNA** | **25** | **8425** | **0.02** | **59** | **11106** | **0.02** |
| **Simple repeats** | **7665** | **320027** | **0.89** | **7520** | **302527** | **0.65** |
| **Low complexity** | **1042** | **52138** | **0.14** | **723** | **33149** | **0.07** |

* Numbers are higher in some cases, as some elements are fragmented.
